# Conceptualizing and quantifying body condition using structural equation modelling: A user guide

**DOI:** 10.1111/1365-2656.13578

**Published:** 2021-09-06

**Authors:** Magali Frauendorf, Andrew M. Allen, Simon Verhulst, Eelke Jongejans, Bruno J. Ens, Henk‐Jan van der Kolk, Hans de Kroon, Jeroen Nienhuis, Martijn van de Pol

**Affiliations:** ^1^ Department of Animal Ecology Netherlands Institute of Ecology Wageningen The Netherlands; ^2^ Centre for Avian Population Studies Wageningen The Netherlands; ^3^ Department of Animal Ecology and Physiology & Experimental Plant Ecology Radboud University Nijmegen The Netherlands; ^4^ Groningen Institute for Evolutionary Life Sciences University of Groningen Groningen The Netherlands; ^5^ Sovon—Dutch Centre for Field Ornithology Nijmegen The Netherlands

**Keywords:** body condition index, composite variable, fitness component, latent variable, multiple regression, multiple‐indicator multiple‐cause model, path analysis, principal component analysis

## Abstract

1. Body condition is an important concept in behaviour, evolution and conservation, commonly used as a proxy of an individual's performance, for example in the assessment of environmental impacts. Although body condition potentially encompasses a wide range of health state dimensions (nutritional, immune or hormonal status), in practice most studies operationalize body condition using a single (univariate) measure, such as fat storage. One reason for excluding additional axes of variation may be that multivariate descriptors of body condition impose statistical and analytical challenges.
2. Structural equation modelling (SEM) is used in many fields to study questions relating multidimensional concepts, and we here explain how SEM is a useful analytical tool to describe the multivariate nature of body condition. In this ‘Research Methods Guide’ paper, we show how SEM can be used to resolve different challenges in analysing the multivariate nature of body condition, such as (a) variable reduction and conceptualization, (b) specifying the relationship of condition to performance metrics, (c) comparing competing causal hypothesis and (d) including many pathways in a single model to avoid stepwise modelling approaches. We illustrated the use of SEM on a real‐world case study and provided R‐code of worked examples as a learning tool.
3. We compared the predictive power of SEM with conventional statistical approaches that integrate multiple variables into one condition variable: multiple regression and principal component analyses. We show that model performance on our dataset is higher when using SEM and led to more accurate and precise estimates compared to conventional approaches.
4. We encourage researchers to consider SEM as a flexible framework to describe the multivariate nature of body condition and thus understand how it affects biological processes, thereby improving the value of body condition proxies for predicting organismal performance. Finally, we highlight that it can be useful for other multidimensional ecological concepts as well, such as immunocompetence, oxidative stress and environmental conditions.

## Graphical abstract

Structural equation modelling (SEM) is a powerful and flexible statistical tool that can lead to models of higher predictive power and with more accurate as well as precise estimates compared to conventional approaches. The authors encourage researchers to consider SEM as a flexible framework to quantify the multivariate nature of body condition and thus understand how it affects biological processes.

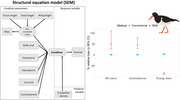

## 1 INTRODUCTION

Body condition is (by definition) a key determinant of an individual's fitness (Wilder et al., 2016). Understanding how best to quantify body condition therefore has implications in behavioural (Kirk & Gosler, 1994), evolutionary (Aubry et al., 2013) and conservation (Stirling & Derocher, 2012) studies, but how to analyse and estimate body condition has remained a subject of debate for decades (Wilder et al., 2016). Notably, there is currently no consensus on a strict definition of body condition, although there is conceptual agreement that the term describes the degree to which an animal's health state influences its performance (e.g. reproduction; Brown, 1996; Peig & Green, 2009). The concept of body condition can thus encompass a wide range of morphological and physiological metrics that describe the nutritional, immune and/or hormonal state of an individual. Which of such metrics are most relevant will depend on the study system and aims, and therefore also on the performance variable of interest (e.g. survival, reproduction, activity; Table 1).

**TABLE 1:** An overview highlighting the diversity of studies investigating body condition with different aspects of an individual's health state and performance measures. Note that this overview is not based on an exhaustive search, as it only aims to illustrate the diversity in health status metrics considered for different performance metrics

Performance\Health status		Nutritional state	Immune state	Hormonal state
Reproduction	Reproductive output (eggs, fledglings)	Abdominal profile index^1^ Residual mass^1^ Nutrients (protein content)^2^	Principal component of packed cell volume, haemoglobin, scaled mass, muscle score, fat score^24^ Carotenoid^25^	Corticosterone^26, 27, 28, 29, 30^
	Milk production	Body condition scoring^3, 4, 5^		
	Lay date	Abdominal profile index^1^		Corticosterone^31^
	Paternal investment (sexual selection, male attraction)	Residual mass^6, 7^ Fulton index (weight/length^3^)^7^ Nutrients (protein content)^2^	Lymphocyte^6^ Parasite load^7^	Testosterone^32^
Survival	Mortality risk through disease (e.g. obesities)	Subcutaneous and abdominal fat^8^ Body Mass Index (mass/length^2^)^9^ Scaled mass index^10^		
	Survival (adult, juveniles)	Principal component of haematocrit, buffy coat, residual mass^11^ Scaled mass index^12^	Principal component of haematocrit, buffy coat, residual mass^11^ Parasite load^12^ Carotenoid^25^	Testosterone^33^ Corticosterone^34^
Growth	Chick/nestling growth	Ratio (mass/length)^13^ Residual mass (mass ~ pc1(body size))^14^		Corticosterone^14, 35, 36^ Testosterone^37^
	Adult growth	Muscle index^15^ Liver enzyme activity^16^		
Foraging	Foraging efficiency/strategy	Visible fat scores^17^ Ratio (mass/length)^17^ Residual mass^19^		Corticosterone^35^
	Foraging condition	Subcutaneous fat^18^ Plasma parameters (e.g. protein, urea, cholesterol, carbohydrates)^20^ Nutrients (e.g. calcium, phosphorus, nitrogen)^21^ Liver enzyme activity^16^		Corticosterone^38, 39, 40, 41, 42^
Migration	Migratory behaviour	Subcutaneous or abdominal fat scoring^1, 17, 22^		
Response to environment	Response to ecotoxicologically polluted area	Scaled mass index^23^		

Notes

^1^Bêty et al. (2003), ^2^Barry and Wilder (2013), ^3^Russel et al. (1969), ^4^Edmonson et al. (1989), ^5^Roche et al. (2009), ^6^Gleeson et al. (2005), ^7^Neff and Cargnelli (2004), ^8^German et al. (2006), ^9^Nuttall (2015), ^10^MacCracken and Stebbings (2012), ^11^Verhulst et al. (2004), ^12^Souchay et al. (2013), ^13^Beintema (1994), ^14^Williams et al. (2008), ^15^Brown and Murphy (2004), ^16^Metón et al. (1999), ^17^Swanson et al. (1999), ^18^Bearhop et al. (2004), ^19^Durell et al. (2001), ^20^Alonso‐Alvarez et al. (2002), ^21^Nie et al. (2014), ^22^Wiersma and Piersma (1995), ^23^Tête et al. (2013), ^24^Milenkaya et al. (2015), ^25^Simons et al. (2012), ^26^Angelier et al. (2010), ^27^Ouyang et al. (2011), ^28^Kouwenberg et al. (2013), ^29^Perez et al. (2016), ^30^Montreuil‐Spencer et al. (2019), ^31^Schoech et al. (2004), ^32^Saino and Møller (1995), ^33^Dufty (1989), ^34^Rivers et al. (2012), ^35^Kitaysky et al. (2003), ^36^Sheriff et al. (2009), ^37^von Engelhardt et al. (2006), ^38^Jimeno, Briga, et al. (2017), ^39^Jimeno et al. (2018), ^40^Jimeno et al. (2017), ^41^Marra and Holberton (1998), ^42^Kitaysky et al. (1999).

Despite the multidimensional nature of body condition, most ecological studies operationalize body condition by quantifying a single (univariate) measurable variable (Wilder et al., 2016). In studies in which the energy stores are thought to be important for the individuals performance, often it is approximated by the mass of an individual relative to its size, as this is assumed to reflect internal fat content (Durell et al., 2001; Janssen et al., 2011; Schulte‐Hostedde et al., 2005; see van der Meer and Piersma (1994) for the distinction between energy reserves and energy stores). Well‐known examples include the body mass index in humans (BMI, mass/length^2^; Nuttall, 2015), or the residuals of body mass regressed to one or more variables of structural size (e.g. tarsus or head length; Jakob et al., 1996), which are widely used in avian studies (Peig & Green, 2009). However, two individuals with similar BMI may still differ in various other aspects of their health, quality or vigour (Stevenson & Woods, 2006). The BMI in humans, for instance, reflects different body composition in men and women (e.g. women have more fat and men more muscles; Jackson et al., 2002) and therefore appears to be a poor predictor of morbidity and mortality, as people with similar BMI can have different metabolic health (Roberson et al., 2014). In fact, it has now become clear that different metrics of body condition are also related to aspects other than the energetic state of an individual (Table 1). Such non‐energetic aspects can be an additional or even more appropriate predictor of fitness (Cox & Calsbeek, 2015; McGuire et al., 2018; with fitness measuring the genetic contribution to future generations). For example, an animal can have high lipid content but have low reproductive success if it is deficient in other nutrients (e.g. protein, vitamins, minerals) that it needs to reproduce (Nie et al., 2014).

These examples indicate that operationalizing body condition via univariate descriptors of body energy stores is unsatisfactory for two reasons. First, the underlying assumption that there is a (monotonic) positive association between energy stores and organismal performance has often been falsified. This suggests that it is important to also consider other measures of the health state beyond energy stores, such as physiological measures (Table 1; note that here our point is to emphasize that a variety of body condition measures can be and are used in ecological and (veterinary) health research and that no ‘perfect’ metric exists, i.e. universally suitable for all studies, although we appreciate that in specific studies some condition metrics can be more relevant than other metrics for biological reasons). Second, a univariate approach to body condition is unlikely to fully capture what is essentially a multivariate concept, at the expense of lower power to predict variation in performance. Indeed, efforts to combine several variables into one compositional measure have been able to better predict fitness variation than each of these condition variables separately (Sousa et al., 2007; Verhulst et al., 2004). A solution to these two problems may be to not operationalize condition as one or two major axes of variation among traits that are a priori assumed to be related to fitness (e.g. univariate metrics such as fat content), but rather operationalize condition as the axis of variation (i.e. a specific composite body condition metric) that best explains variance in individual fitness or performance. Such a view is quite similar to how Wilson and Nussey (2010) conceptualized ‘individual quality’, though body condition is always linked to a specific aspect of individual quality (i.e. health state) and is a much more dynamic concept as it fluctuates during lifetime while individual quality is a less flexible characteristic (e.g. social dominance).

Adopting a multivariate description of body condition and identifying which condition index best explains variation in performance or fitness impose statistical and analytical challenges that have thus far not been fully addressed. Studies that aim to describe the multivariate nature of body condition have used a variety of statistical methods (e.g. multiple regression, principal component analyses), which have limited flexibility and implicitly make specific assumptions that are not always recognized. Principal component analyses (PCA) identify axes of body condition based on the correlation patterns among condition variables (Abdi & Williams, 2010) while the weight of a variable in an estimate of condition instead ideally reflects how much it contributes to explaining variation in performance relative to other variables. Multiple regression (MR) models have low flexibility in that they cannot handle variables that are simultaneously response and explanatory variables, which is resolved using stepwise modelling approaches that can potentially cause bias in the parameter estimates (Darlington & Smulders, 2001; Freckleton, 2002). Thus, there is a need for an alternative analytical framework to integrate multiple variables in one condition estimate, with their relative weight depending on their power in explaining variation in fitness, or, more usually, fitness proxies such as survival or reproductive traits (e.g. lay date, egg volume, fledgling number).

To this end, we introduce structural equation modelling (SEM) and show how it can be used to overcome the above‐mentioned challenges in quantifying a body condition index. SEM is a flexible conceptual framework, where statistical and mathematical tools, along with analytical principles are used to learn about a system (Grace, 2006; Shipley, 2016). SEM builds on path analysis, which is a statistical regression analysis that allows one to investigate patterns of effect within a system of variables by examining the impact of a set of predictor variables on multiple, possibly interrelated, response variables. Moreover, SEM extends path analysis by including unmeasured variables. For example, ‘latent variables’ or ‘composite variables’ can be used to summarize multivariate concepts, like body condition, that are not directly measurable themselves. SEM can be used to evaluate competing multivariate models of body condition, and thus learn more about the study system, and quantify the relative importance of different pathways. Although SEM originates from the social sciences (Grace et al., 2010), the capacity for evaluating multivariate hypotheses has also attracted the interest of ecologists, with some aspects of SEM (e.g. latent variables, path analysis) being widely used in specific subfields of ecology (Grace, 2006; Shipley, 2016). Belovsky et al. (2004) suggested that the ecological sciences can be advanced by a better integration of theory and empirical evidence and by studying multiple causes simultaneously, both of which can be addressed with SEM.

We emphasize that the SEM techniques we describe are not novel from a statistical perspective. Instead, our aim is to demonstrate in a comprehensive and educational way its usefulness for the quantification of body condition indices as well as for testing biological hypotheses concerning condition through worked empirical examples on a shorebird species. Data are from a large‐scale study in which we measured a variety of morphological and physiological variables on Eurasian oystercatchers *Haematopus ostralegus*. We provide R‐code of all analyses with annotations to facilitate its usage and as a learning tool for those new to SEM.

## 2 INTRODUCING THE CASE STUDY USED TO EXPLAIN THE CONCEPTUAL FRAMEWORK

### 2.1 The case study system

The global Eurasian oystercatcher population has declined rapidly which can be caused either by reduced reproduction or by reduced survival, and thus our aim is to identify the impact of potential environmental drivers in winter affecting survival. However, survival probabilities of individuals are not directly observable, as we have to infer them from the stochastic realizations of individuals dying or not, leading to information loss (i.e. even more problematic when there is imperfect detection and we have to rely on capture–mark–recapture analysis for inference, which also requires many years of data collection). An additional challenge in our long‐lived study species is that mortality events are rare, with an average annual adult survival rate of ~0.9 (Allen et al., 2019), meaning that there is little variation in this performance metric in a single year. Both challenges limit the opportunity to detect associations between environmental drivers and survival probabilities. Creating a body condition index that is closely related to survival gives the opportunity to use a more informative fitness proxy in the future based on data that are less time‐consuming to collect in the field (compared to the long‐term survival data).

**FIGURE 1:**
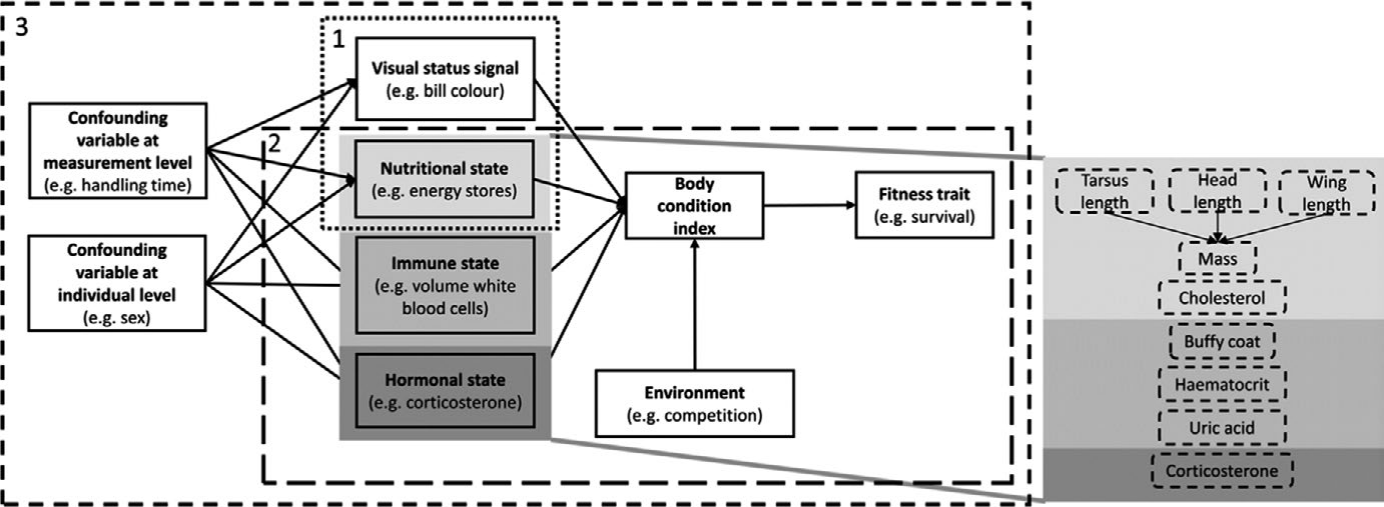
Conceptual framework of the case study and how we will develop the model in three sections. The numbers in the top left of each box with interrupted lines indicate the section where the analysis of those variables is discussed. Box 1 (dotted line): Summarizing the multidimensional nature of body condition by variable reduction; Box 2 (long dashed line): Quantify body condition based on a fitness trait; Box 3 (short‐dashed line): High flexibility, comparing competing hypotheses, and model performance. The grey shaded area in box 2 zooms in on the specific condition variables used and discussed in the oystercatcher case study (see Section 2) and the three axis of health status illustrated in Table 1

Therefore, the overall aim of the case study is to use health status measurements on captured birds to derive an integrative body condition index that maximizes the explained variation in a fitness component (survival) and helps to identify how environmental drivers (competitor density) affect body condition (Figure 1).

To quantify body condition, 774 oystercatchers were caught with nets at high‐tide roosts and colour‐banded in the Netherlands during 19 catching events spanning two winters (2016–2017: 196 birds; 2017–2018: 578 birds; Figure S1; Table S1). Several morphological and physiological variables were measured that could potentially reflect the immune, hormonal and nutritional state of an individual. In addition, we measured (carotenoid based) bill colour that may serve as a visual status signal of an individual's body condition to conspecifics (Figure 1). We considered these variables based on previous studies that have shown that they can be associated with either body mass or survival (Text S1). Some variables that we are not primarily interested in may influence multiple condition variables (Norte et al., 2009), and thereby could explain some of the intercorrelation between various condition variables. Accounting for such confounding variables may clarify to what extent these condition variables are different axes of body condition (Figure 1). By considering effects of handling time (proportion of time between capture and measuring) on condition variables, we aim to statistically account for time‐dependent mass loss and possible effects on other physiological variables that we are not interested in. Similarly, we want to consider confounding effects of individual characteristics such as age, sex and bill shape. The age of the birds was classified in 1st, 2nd, 3rd calendar year and adults (>3rd calendar year). We focused only on sub‐adults (2nd and 3rd calendar year) and adults (>3rd calendar year) in the analysis because the number of sampled juveniles (1st calendar year) was small (*n* = 28; Table S1). Adult oystercatchers cannot be accurately aged and therefore we do not consider any possible effect of senescence on body condition or survival. A long‐term study population in the Netherlands, in which there are many known‐age individuals (e.g. marked at birth), also did not reveal any evidence that survival or body condition varies systematically with age among adults. Bill tip height was used as a proxy for the type of individual's feeding specialization ranging from worm specialists (pointed bill characterized by a low bill tip height) to shellfish specialists (blunt bill characterized by a high bill tip height; van de Pol et al., 2009).

We relate the body condition index to survival in the year following capture. The survival estimates (mean [*SD*], min, max: 0.87 [0.06], 0.73, 0.92) used in the case study were generated from a multi‐state live and dead recoveries model and therefore are on an area level. See Text S1 and Table S2 for details on data collection and capture–mark–recapture modelling.

### 2.2 The conceptual framework applied to the case study

In the following three sections, we describe step by step how we quantify body condition with SEM in the oystercatcher population. We first describe how to summarize the multidimensional nature of body condition using a variable reduction approach (box 1 in Figure 1). We then describe how to quantify body condition based on a fitness trait (box 2 in Figure 1). The last section combines the first two steps but also adds confounding variables to the model (box 3 in Figure 1) to illustrate the high flexibility of SEM. For comparison, we start all sections by analysing the data with a conventional statistical approach before demonstrating the SEM approach. In the last section, we compare the model performance between SEM and a conventional approach.

## 3 SECTION 1: SUMMARIZING THE MULTIDIMENSIONAL NATURE OF BODY CONDITION: VARIABLE REDUCTION

In this section, we will illustrate how the multivariate concept of body condition can be summarized and compared to alternative models by considering two variables of health status, namely ‘energy stores’ and ‘bill colour’ (box 1 in Figure 1).

### 3.1 Conventional method: Principal component analysis (PCA)

A conventional method that combines several measures into one compositional measure is PCA, a multivariate statistical technique that is used in most scientific disciplines (Abdi & Williams, 2010). PCA has also been used to create condition indices (e.g. Bearhop et al., 2004; Milenkaya et al., 2015; Verhulst et al., 2004). PCA summarizes multiple variables using the shared correlation structure to derive the principal components. If the first principal component (PC1) explains the majority of the variation, the other principal components can be ignored without much information loss, and variable reduction is achieved through using PC1 as a single new condition variable. The use of PCA is suitable for the (adult) bill colour and energy stores examples because the different (adult) bill colour and energy stores metrics are strongly linearly interrelated (Figure S2; Table S3), and such intercorrelation is a requirement for PCA.

Concerning the energy stores, we considered two alternative variables in our analysis that reflect some of the diversity of measures used in the ecological literature (Peig & Green, 2009): (a) the residuals of mass regressed against structural size measures: head length, tarsus length and wing length and (b) the ratio of mass to size (tarsus length). Both variables are assumed to be positively related to fitness (Stevenson & Woods, 2006) and are highly intercorrelated (*r* = 0.91, Figure S2). We note that using ratios sometimes introduces underlying hidden assumptions and mathematical limitations that may need consideration; for a full discussion see Atchley and Anderson (1978). PC1 explained 96.6% of the variation (residuals: 0.98; ratio: 0.98; *n* = 804; Figure S3 and Table S4). We therefore can conclude that the PCA performs well in summarizing both correlated traits into a single variable.

For bill colour, we ran a PCA with the variables hue, chroma and luminance (components of a colour; Quesada & Senar, 2006) for both age classes separately, as we clearly see differences between the intercorrelation of the variables between adults and sub‐adults (Table S3; Figure S6). The PC1 of bill colour explained 88.8% and 69.4% of the variation for adults and sub‐adults, respectively, and loading was approximately equal for each of the three components, except for hue in sub‐adults (adults: hue = 0.99, chroma = −0.90, luminance = 0.95, *n* = 599; sub‐adults: hue = −0.49, chroma = 0.99, luminance = 0.92, *n* = 163; see Figures S4–S6 and Tables S5 and S6). For bill colour, we therefore also can conclude that the PCA performs well in summarizing all three correlated colour traits into a single variable.

### 3.2 SEM method: Confirmatory factor analysis using latent variables

**FIGURE 2:**
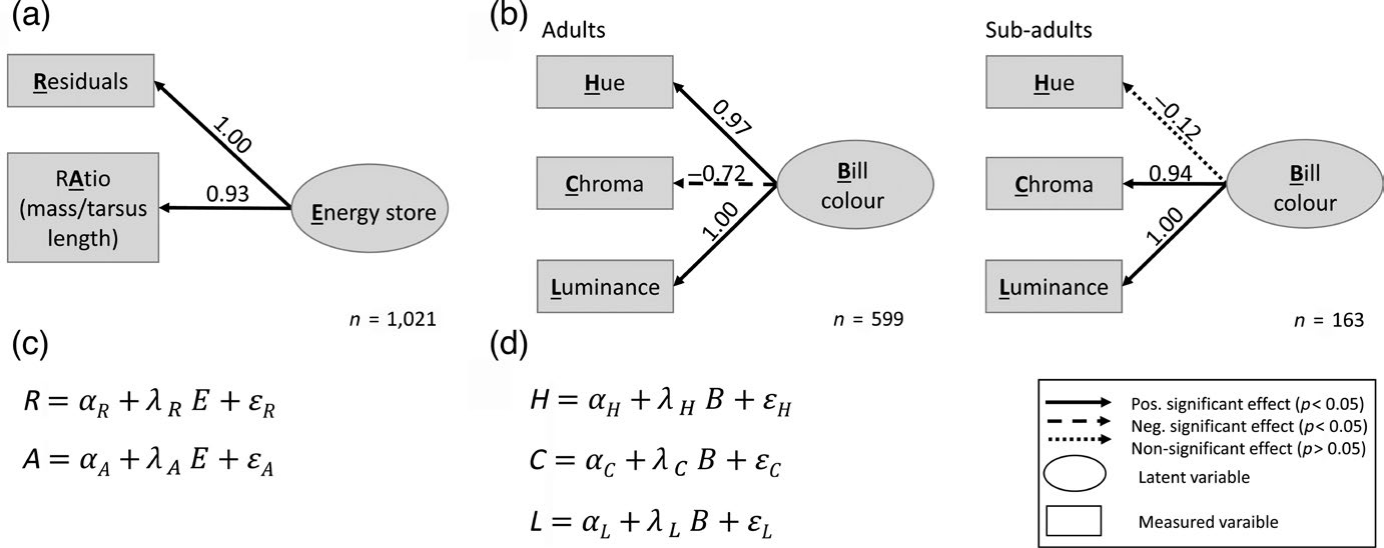
Path diagrams of the confirmatory factor analysis (CFA) for the latent variables: (a) energy stores (determined by mass corrected for size [equivalent to residuals on the mass axis] and by the ratio of mass/tarsus length) and (b) bill colour (determined by hue, chroma and luminance) for the two age classes. Boxes indicate measured variables; circles represent latent variables. Arrows indicate positive significant (solid line), negative significant (dashed line) or non‐significant (dotted line) path strengths with values of standardized coefficients shown next to each line. Standardized coefficients are estimates expressed in equivalent units to make it possible to compare different path strengths in the model. Standardized coefficients (scale standardization) are estimated by multiplying the slope of the ratio of the standard deviation of the predictor and response variable (Lefcheck, 2019). Latent variables are described by arrows going away from the latent variable indicating that they are measured by the observed variables. (c) and (d) show the equations belonging to models (a) and (b), respectively. *α*, *λ* and *ε* illustrate the intercept, slope coefficients and residual error, respectively. See Codes S1 and S2 for detailed model results and specifications

A closely related statistical approach to PCA that is available in the SEM framework is a confirmatory factor analysis (CFA). CFA includes two types of variables: observed variables and latent variables which are graphically represented, respectively, by boxes and circles (Figure 2). The ‘confirmatory’ part speaks to the knowledge one has of the theory behind the relationship between the latent and their observed variables. Latent variables are hypothetical or theoretical variables (constructs) that we assume to exist but for which we have no direct measurements, and are therefore referred to as ‘unmeasured variables’ (Grace et al., 2010). Latent variables are used in models to represent an underlying process while observed variables serve as indicators of the effects or manifestations of the latent factors (Grace & Bollen, 2008). Latent variables play an important role in SEM because they represent a bridge between observed data and theoretical generalization (Grace & Bollen, 2008), and a part of the concept of body condition is a prime example of a latent theoretical concept, as these are latent variables made up of typically correlated condition measures (e.g. ratio and residual body mass or BMI). CFA provides a conceptual advantage: we often choose a single indicator as a surrogate for a latent concept (e.g. hue representing the more general concept ‘colour'). Including more indicators (e.g. chroma, luminance) helps to generalize this phenomenon by testing that the result is not impacted by the choice of any single indicator. This emphasizes that CFA/SEM is a logical choice in the context of the multivariate nature of body condition. The purpose of CFA is to statistically test the ability of a hypothesized (factor) model to reproduce a set of sampled data (usually through a variance–covariance matrix; Nusair & Hua, 2010). In a CFA, the estimated values for all records can be extracted and used for instance for plotting in a similar way as is done with PCA.

The use of latent variables (as a variable reduction tool) is common in social sciences and psychology (Bollen, 2002; Grace et al., 2010) and there is a growing interest in ecological research also. Latent variables are, for instance, used to quantify species performance in plant research (Grace et al., 2016; Travis & Grace, 2010; Visser et al., 2018), or morphometrics in birds (Araya‐Ajoy et al., 2019; Cubaynes et al., 2012; Pugesek & Tomer, 1996) and mammals (MacKay et al., 2017; Nespolo et al., 2003), as well as to quantify environmental conditions (Guan et al., 2016; Souchay et al., 2018), behaviour in mammals (Dochtermann & Jenkins, 2007), fish (Sprenger et al., 2012) and birds (Araya‐Ajoy & Dingemanse, 2014; Dingemanse et al., 2010; Jablonszky et al., 2018; Krams et al., 2013; Moiron et al., 2019).

We created the variable ‘energy stores’ as a latent variable construct in the SEM framework using CFA (Figure 2a). The variable energy stores was defined by both (a) the ratio of mass to tarsus length and (b) the residual of mass regressed to three structural size measurements (head length, tarsus length and wing length). Model results (Code S1) indicate that the ratio and residual variables are positively related to the latent variable energy stores (Figure 2a).

Similarly, we can create a variable ‘bill colour’ as a latent variable in the SEM framework (Figure 2b; Code S2), which on a more abstract level can be seen as a common factor of several observed variables (hue, chroma and luminance) so that its associated biological consequence can be viewed to occur at the level of a bill colour factor, rather than at the level of each observed variable. Hue values indicate a scale from orange to yellow, whereas luminance and chroma give information on the lightness and saturation of the colour (see Figure S7 for colour component interpretation). Since we expect that the relationship among the colour variables differs between the two age classes, we conduct a multi‐group CFA. The structural equation model (Figure 2b, Code S2) shows that in adults, the hue and luminance are positively intercorrelated, whereas chroma is negatively intercorrelated. In accordance with the PCA results, the CFA for sub‐adults shows low intercorrelation of hue with chroma and luminance and hue contributes less to the estimate of ‘bill colour’ in this group.

To conclude, CFA is a technique within SEM that is conceptually related to the variable reduction technique PCA, and in our case study the values from the latent variable and PC1 are highly correlated for the energy store (*r* = 0.98) and the bill colour model (adults: *r* = 0.95; sub‐adults: *r* = 0.92), showing linear relationships over the entire range (Figure S8). Note that at this point ‘bill colour’ and ‘energy stores’ are latent variables which are not yet related in any way to condition. This will follow in the next two sections.

## 4 SECTION 2: QUANTIFY BODY CONDITION BASED ON A FITNESS TRAIT …

PCA and CFA identify the body condition axes of ‘bill colour’ or ‘energy stores’ based on the correlation patterns among observed variables (Abdi & Williams, 2010), but not on how well the different observed variables explain variation in fitness. It is not unlikely that different body condition variables explain different parts of the variation in fitness, as they may represent different aspects (‘axes’) of body condition. For example, energy stores may explain variation in survival due to starvation while immunological measures may explain variation in survival due to disease. Arguably, ecologists may often have no clear reason to assume a priori that the shared correlation patterns among condition variables necessarily explain most variation in fitness. Thus, different observed variables may have different weights in explaining variation in fitness, and rather than making a priori assumptions about the weights, SEM can be used to build a model where the weights are part of the outcome. Furthermore, when researchers use PCA/CFA, it is often implicitly assumed that a relationship between the measured condition variable (e.g. mass corrected for size) and fitness prospects exists. However, the assumption that there is a monotonic positive association between energy stores and fitness prospects has often been falsified (e.g. Barry & Wilder, 2013; Verhulst, 1998) and it is therefore important to check this key assumption.

We will illustrate the quantification of a body condition index based on a fitness trait with our case study, where our overall goal is to create a body condition index that maximizes the explained variation in annual survival (box 2 in Figure 1).

### 4.1 … with a conventional method—Multiple regression analysis (MR)

MR estimates the relationships between a response variable (e.g. fitness component) and one or more predictor variables (condition variables; Nusair & Hua, 2010). A key characteristic of MR analysis is that it aims to show how variation in one variable can be explained by others. A limitation of MR is that they cannot handle variables being both response and predictor at the same time and therefore are limited to the examination of single process at a time. The commonly used work around is to take a multi‐step approach of performing several MR analyses sequentially (e.g. Skarpaas et al., 2011). In our hypothesized conceptual model (box 2 and grey shaded area in Figure 1), body mass is seen as a predictor variable when used to explain variation in the body condition index, but simultaneously as a response variable when regressed to different structural size measurements (e.g. tarsus length; Figure 4a).

We aimed to quantify a body condition index that explains variation in survival and is based on six measured condition variables characterizing the nutritional state, immune state and hormonal state (box 2 in Figure 1; Figure 4a). With an MR approach, we would answer this question by conducting the following four sequential steps. We first need to regress mass on three structural sizes (tarsus length, head length and wing length) to account for individual variation in structural size that we are not interested in (step 1). Next, we extract the residuals of this model, and regress them together with the five other condition variables (buffy coat) on the survival variable (step 2). Then, we extract the predicted values of the survival model as a body condition index (step 3). Only now, in a final fourth step, can we tackle our main question by regressing the body condition index to the environmental variable ‘competitor density’ to determine the effect of density on ‘condition’, and thereby answer how competition (e.g. for food sources) affects survival through the condition of an individual. Conducting multiple steps using the residuals of a model as input for another model (also called residual analysis) can lead to biased parameter estimates, especially if independent variables are not totally uncorrelated (Darlington & Smulders, 2001; Freckleton, 2002).

### 4.2 … with SEM method—Composite variable and MIMIC

**FIGURE 3:**
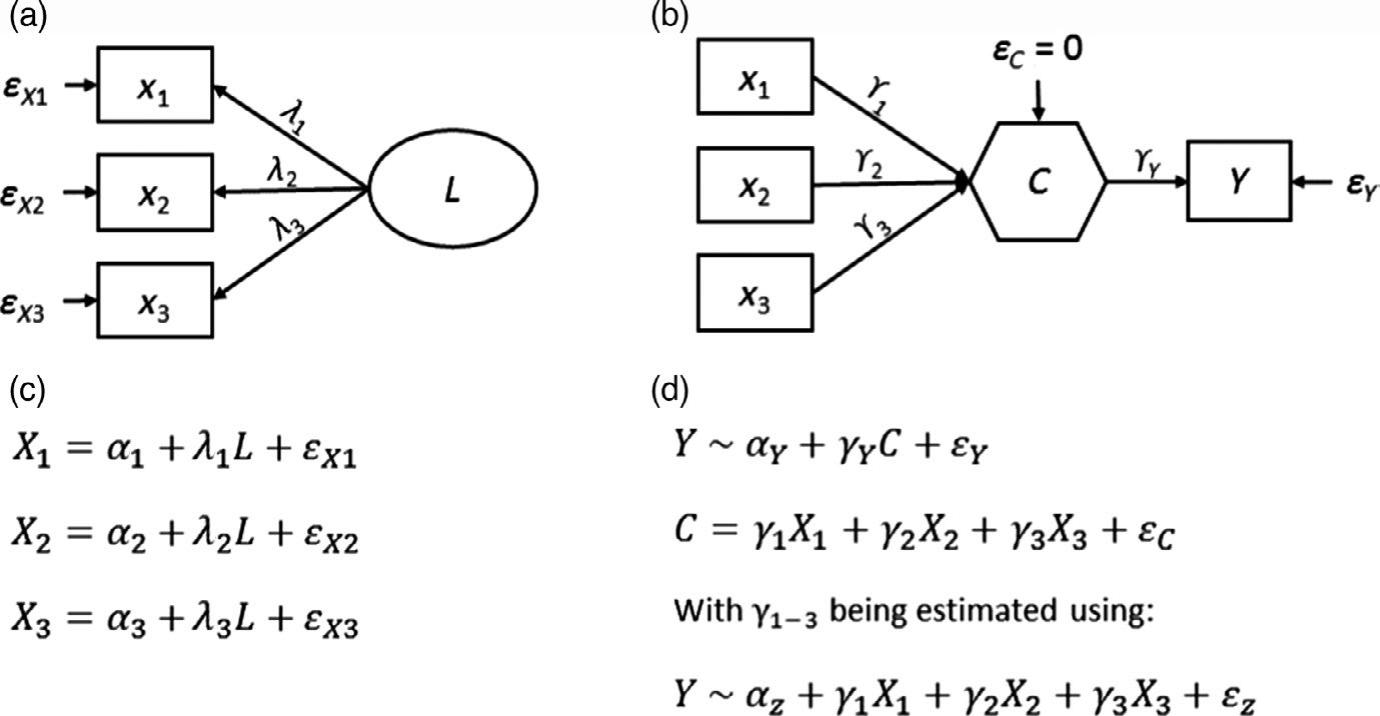
Graphical representation of a model using a latent (a) variable and a composite (b) variable. The path diagram shows the relationships between observed variables (*X*'s in boxes) and (a) latent (*L*; in circle) or (b) unknown‐weight composite (*C*; in hexagon) variable. Note that for the unknown‐weight composite in (b), a response variable *Y* is required, while for defining a latent variable in (a) this is not a necessity, but could be added if useful. The equations for the latent and composite variable are shown in (c) and (d), respectively. (c) *X* is the result of the influence of the latent variable (*L*), proportional to *λ* (=loading), plus the error *ε* and the intercept *α*. For details on how *L* and *λ* are derived from data, see chapter 4 in Grace (2006); (d) composite variable *C* is equal to the sum of the effects of *X*s plus the error which is fixed to 0. The response variable *Y* is a result of the intercept (*α_Y_*), the slope coefficient *γ*, *C* and the error *ε*

The hypothesized conceptual model (box 2 in Figure 1) can be fitted in a single model within the SEM framework using ‘composite variables’. Whereas ‘regular’ latent variables represent an unmeasured variable that leads to correlation between the observed variables (Figure 3a), composite variables represent the collective effect of a group of observed or latent variables (Figure 3b; Grace & Bollen, 2008). Composite variables can be seen as a special case of a latent variable and are represented by a latent variable with zero error, which signifies that it is completely specified by its causes (Grace, 2006). Thus, if condition variables are strongly correlated, we advise to use latent variables to integrate them, whereas in composite variables, the observed contributing condition variables do not necessarily need to be correlated, which is an important flexibility of its application (Nusair & Hua, 2010).

**FIGURE 4:**
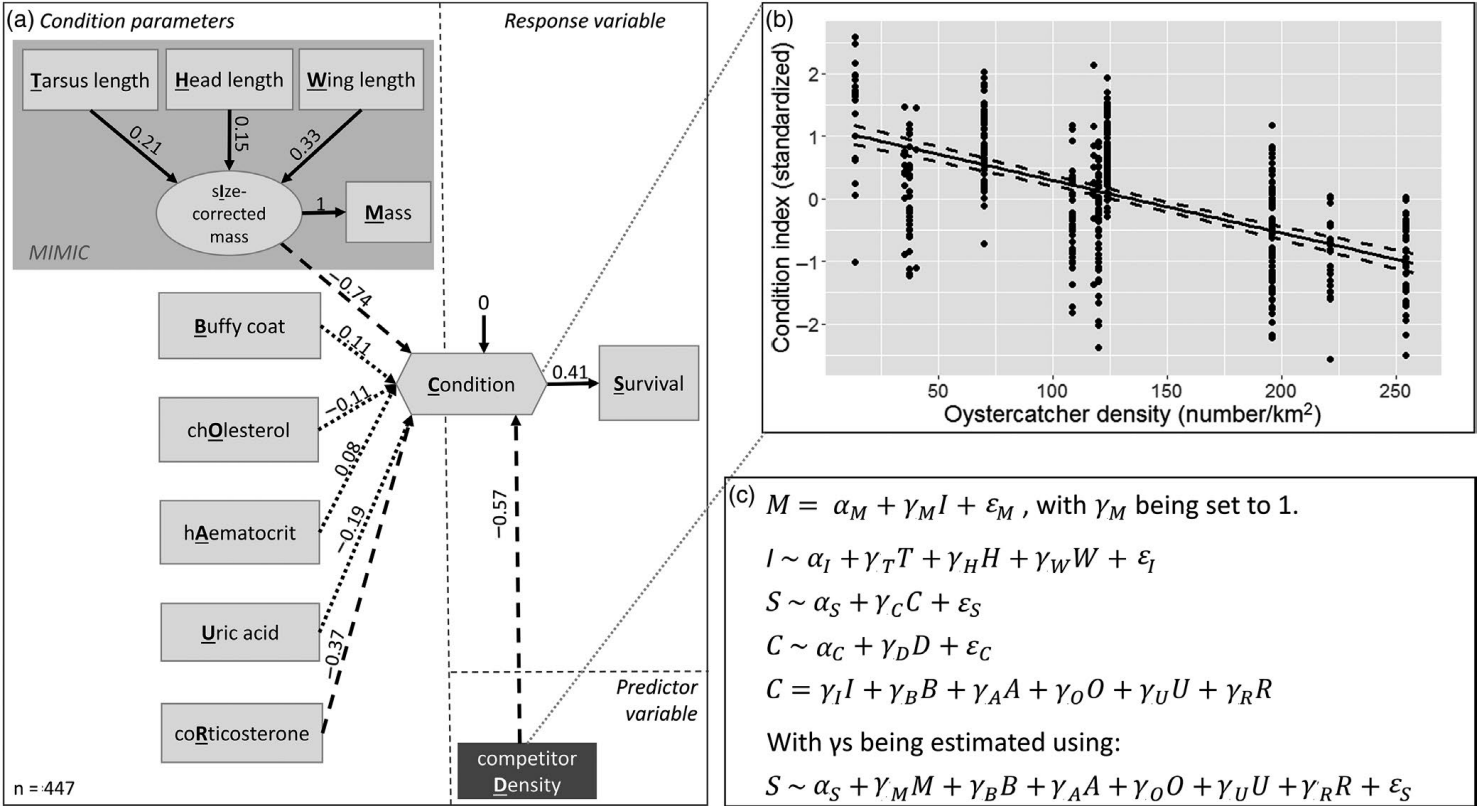
(a) SEM model of body condition based on a fitness trait for oystercatchers. Solid and dashed lines indicate a positive and negative significant (*p* < 0.05) path, respectively. Values indicate the standardized path strength (see Figure 2 for explanation). Dotted lines indicate non‐significant pathways (*p* > 0.05). (b) visualizes the relationship of density on the body condition index as estimated by the SEM. Dots indicate density data points and model estimate of body condition index for each individual, whereas the solid line shows fitted line of the model with the lower and upper 95% confidence interval as dashed lines. Note that individuals have the same density value when they were caught at the same location. (c) shows the equations of the SEM depicted in (a), emphasizing that oystercatcher density (dark grey box) does not shape the condition index, only the measured condition variables (light grey boxes) do (as can be seen from the two separate equations for *C*). The tilde‐signs denote the use of multiple regression within the SEM (e.g. mass as a function of size), while the ‘equal’‐signs denote the estimation of the composite and latent variables. The latent variable ‘size‐corrected mass’ was included as part of the MIMIC subpart of the SEM (shaded area in a) to make sure that condition was affected by size corrected for mass and not by mass alone. For further explanation, see the text. For R code, see Code S3

In contrast to the use of latent variables in ecology, composite variables have been long recognized as a potentially important element of SEM, but have received very limited use in ecology among others because of a lack of theoretical consideration (Grace & Bollen, 2008; Hopcraft et al., 2012; Pugesek & Tomer, 1996). Composite variables come in two types. One is a *fixed‐weight composite*, in which the loadings (*ϒ* in Figure 3d) from the causes are specified a priori (Grace, 2006). An example is the ‘importance value’ in plant ecology, which is defined as the unweighted sum of the relative density, relative abundance and relative frequency (usually for a species within a community). A second type of composite is the *unknown‐weight composite* (Grace & Bollen, 2008). The unknown weight composite is related to a multiple regression predictor, where some weighted combinations of causal influences maximize variance explanation in one or more response variables, and weights are thus estimated from the data. The concept of body condition (box 2 in Figure 1) is a prime example of an ‘unknown‐weight composite’ as first, the six condition variables (Figure 4a) show low intercorrelation (Figure S2) and second, we do not know a priori how important each condition measure is for our fitness proxy survival.

We created a condition index making use of a composite variable defined as a weighted sum of the condition variables, with the weights reflecting the dependency of survival on each of the condition variables (Figure 4a). This step is basically the same as in an MR. The values of the condition index variable for each record can be extracted from the SEM model (Figure 4a) so that the relationship between condition index and density can be plotted (Figure 4b). Furthermore, building a condition index as a composite variable can also be done by relating it to other fitness traits like reproduction (see Section 6), or the weights can be used to quantify the condition of birds captured in future studies, without the need for measuring their survival. We note that the six condition variables were at most weakly correlated (e.g. correlation between cholesterol and corticosterone was *r* = −0.02; Figure S2), and using a latent variable as part of a CFA approach would thus not be an alternative to the composite variable approach taken here.

In an MR, it is not possible to handle mass as a response variable and at the same time as an explanatory variable, forcing the use of other solutions such as using the residuals of mass (as illustrated in Figure 2a). An advantage of the SEM approach is that there is no need to first extract the residuals (of body mass on size) and then summarize the variables; instead, we can include the size, mass and size‐corrected‐mass variables in the same model (Figure 2; see also next paragraph). In addition, in the same SEM, we specify condition as a function of survival probability, and in a separate equation (but in the same model; Figure 4c) regress density on the condition index (see Code S3 for model specifications and R‐code). This has the additional advantage that an overall model fit can be assessed, to quantify to what extent the hypothesized model fits the sample data (Musil et al., 1998) or to compare it with potential alternative models, which will be discussed in detail in Section 3.

Since larger individuals may have a higher body weight, we want to account for the size when including mass as a condition measure in the model. This is often done by taking the residuals of mass regressed on structural size variables (Jakob et al., 1996; Peig & Green, 2010). SEM avoids using residuals (as in Figure 2a) when considering mass corrected for size as a condition variable using a technique called the multiple‐indicator, multiple‐cause model (MIMIC). MIMIC incorporates covariates of interest which are modelled under a latent variable framework (Chang et al., 2020). Specifically, in our case study, we create a new latent variable (‘Size‐corrected‐mass’; Figure 4) affecting only one observed variable ‘mass’ with a default factor loading of 1. The structural size covariates (tarsus length, head length and wing length) affect the latent variable ‘Size‐corrected‐mass’, rather than the observed variable mass. By incorporating the size variables as covariates in this way in SEM, the latent construct ‘Size‐corrected‐mass’ allows for the model to estimate the relationship of mass on condition while accounting for the effect of size‐variables on mass, similar to taking the residuals of mass regressed on size when using a stepwise MR approach. In addition, there may be relationships between the size variables and other condition variables (e.g. cholesterol) that could be considered in the model.

So far, we only considered linear relationships, but often relationships are curvilinear in biology. To illustrate how to model nonlinear relationships, we run the model from Figure 4 with an additional variable ‘squared mass’ (Code S4). By doing so, we can investigate a possible nonlinear relationship of mass on condition. However, in this case study, squared mass does not show a significant effect on condition (*p* = 0.07, Code S4), and we henceforth ignore it for reasons of simplicity.

The model results show that size‐corrected mass and corticosterone have a negative effect on the condition index, whereas buffy coat, haematocrit, uric acid and cholesterol show smaller (non‐significant) effects on condition (Figure 4a). The condition has a path strength of 0.41 on survival, explaining 17% of the spatial variation in survival within the Netherlands (Code S3; Figure S9). Oystercatcher density shows a strong (−0.57) negative association with the body condition index (Figure 4b; Figure S10).

## 5 SECTION 3: HIGH FLEXIBILITY, COMPARING COMPETING HYPOTHESES, AND MODEL PERFORMANCE OF SEM

In this section, we combine the two models from Sections 1 and 2 and include confounding variables related to measurement procedure (handling time) and individual characteristics (sex, feeding specialization and age class; box 3 in Figure 1). Next, we compare the model performance of a SEM approach and a conventional statistical approach.

### 5.1 Model results and interpretation

**FIGURE 5:**
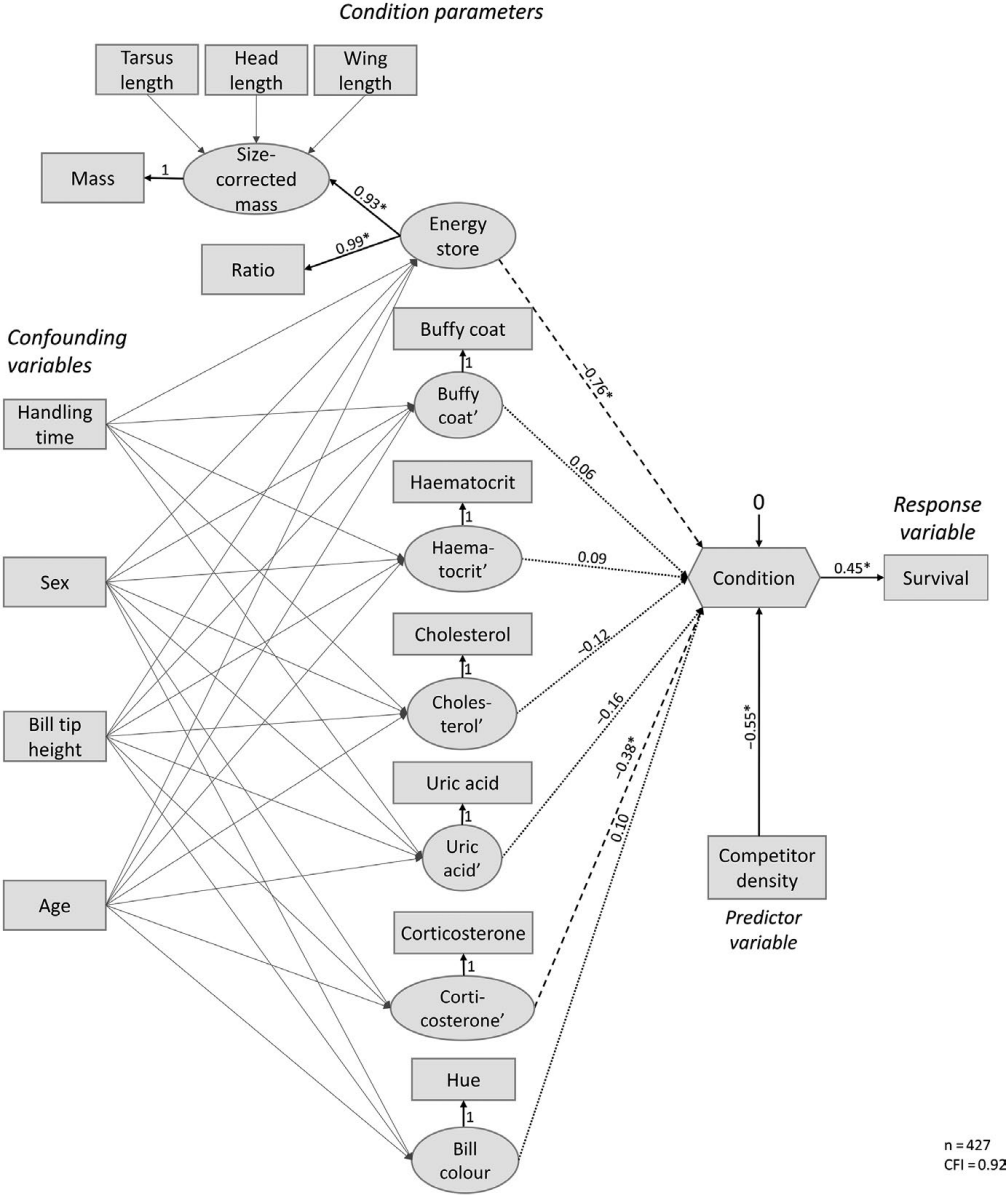
Full SEM model including latent (circles), composite (hexagon) and measured (squares) variables as well as confounding, predictor and response variables. Values indicate the standardized path strength (see Figure 2 for explanation). Solid and dashed lines indicate a positive and negative significant (*p* < 0.05) path and are indicated by an asterisk next to the standardized path strength, respectively. Dotted lines indicate non‐significant pathways (*p* > 0.05). For simplicity, we only documented the path strength of the condition, predictor and response variables. The MIMIC part of the condition variables (buffy coat, haematocrit, cholesterol, uric acid and corticosterone) contains latent variables of the observed condition variables (indicated by an apostrophe) that are corrected for confounding variables (e.g. sex, age). ‘Bill colour’ and ‘size‐corrected mass’ are also MIMIC parts (corrected for confounding variables) within the model, but have another biological meaningful name (rather than the name of the measured variable) and therefore are not noted without apostrophe

To construct the full model (Figure 5), we connect the latent variable ‘energy store’ (Figure 2a) with the composite variable body condition (Figure 4). In addition, by using MIMIC modelling, we correct for the effect of measurement procedure (handling time) and individual (sex, age)‐related confounding variables to account for its effect on the condition variables. The correlation structure of the three colour parameters (hue, chroma and luminance) differs strongly between the two age classes. The colour parameters are only highly correlated in adults and not in sub‐adults (Table S3) as illustrated in the multi‐group CFA (Figure 2b). Therefore, it is not useful to use a latent variable for bill colour in this example. Instead, for simplicity, we use only hue as a measure of bill colour (Figure 5), which we expect to have more variation giving information on the colour in contrast to luminance and chroma, which reflects lightness (Figure S7). For completeness, we illustrate alternative model structures to handle the different correlation structures between age classes in Figure S11.

Whereas MR assumes independence of error terms, which may bias the estimates (James et al., 2013), it is possible to specify non‐independence of error terms in SEM. For instance, in our example, we expect the error term of haematocrit and cholesterol to be correlated because cholesterol is quantified as a concentration in blood, which means that it is related to the proportion of haematocrit in the blood (Code S5). Similarly, in the example of modelling nonlinearity in mass using squared mass as additional variable, we can specify that the error terms of mass and its squared version are correlated.

The full model (Figure 5) shows that the latent variable energy stores is a result of the positive influence of mass (corrected for structural size variables and handling time) as well as the ratio of mass divided by tarsus length. We also find that individuals with a higher bill tip height (blunter shaped bill) show a more yellowish bill (standardized estimate = 0.22, *p* < 0.001; Code S5). In addition, males have a more orange than yellowish bill compared to females (standardized estimate = −0.25, *p* < 0.001; Code S5). Most importantly, we find that energy stores and corticosterone have a negative effect on the condition, whereas the other condition variables (buffy coat, haematocrit, cholesterol, uric acid and bill colour) show weaker (non‐significant) effects on the condition index.

These aforementioned results emphasize the point made in the introduction that we often assume positive relationships with performance or fitness (e.g. high energy stores benefit survival), but that those assumptions do not always hold true. We stress that the negative contribution of the latent construct ‘energy stores’ to body condition results from a negative correlation between mass (corrected for size) and survival (Figure S12), and is thus not a result of the (SEM) modelling procedure itself. A possible explanation for the negative relationship of energy stores with condition may be that birds with higher condition are mainly in areas with more food and therefore do not need to store that many resources, as the food source is more reliably available (Cuthill et al., 2000). Another possible reason may be that the number of predatory birds (e.g. peregrine falcon *Falco peregrinus*) in the oystercatcher wintering grounds increased during recent decades (Sovon Vogelonderzoek Nederland, 2018) which may increase the survival of lighter, more agile individuals by allowing them to escape predation. Lastly, it is important to keep in mind that we here analyse site‐level variation in mortality rather than individual‐level mortality, and between and within site (population) patterns need not be similar. Thus, the negative relationship between energy stores and condition does not exclude that energy stores in principle are important for starvation just that under conditions of high food stocks or predator abundance encountered in our study, energy stores may potentially be unrelated to starvation and instead reflect other aspects, such as foraging efficiency or agility.

We a priori hypothesized a positive relationship of body mass on the condition index, and therefore called the latent variable ‘energy stores’. We can learn from the outcome of this model that our data are not consistent with the theoretical ideas that led us to model the concept of energy stores in the way we did. However, our latent variable ‘energy stores’ could be consistent with interpreting this latent variable as the concept ‘mass‐related agility’ of an individual, but this hypothesis would require further testing. We note that the concept ‘mass‐related agility’ could still be thought of as a condition variable, as it reflects an aspect of health status (responsiveness to danger) and predicts performance (the fitness proxy survival).

Our results also confirmed the expected negative relationship between corticosterone and the condition index, meaning that birds at sites with higher survival had lower corticosterone levels. Corticosterone fulfils its main functions by mobilizing stored resources to facilitate upregulating metabolism for coping with increased energetic challenges (Jimeno et al., 2018). Thus, birds coping with harsh environmental conditions may show elevated corticosterone secretion (Angelier et al., 2010; Jimeno et al., 2017), and our results are consistent with this idea.

We included bill colour as a potential variable affecting individuals' condition, as we hypothesized a priori that such a carotenoid‐based trait is likely to be sensitive to nutritional state and may thus serve as a visual status signal of the individual condition to conspecifics meaning that individuals with a more reddish bill have higher condition. However, on the other hand, if bill colour would be an ‘honest’ signal for sexual selection, it may also be costly and could result in a negative relationship with survival. The pathway of bill colour on condition shows no significant effect on the condition index and survival. We can learn from the model that bill colour is not a useful condition variable that predicts variation in survival performance in this population.

Finally, we found that the body condition index shows a strong (−0.55) negative association with oystercatcher density, whereas body condition positively affected annual survival probability (0.45 standardized path coefficient; Figure 5). This result suggests that that competition may affect the survival through body condition, and that this mediating effect of condition is quite strong: a one standard deviation decrease oystercatcher density at a site is associated with a quarter standard deviation increase in annual survival (−0.55 × 0.45 = −0.25; rules of path tracing; e.g. Grace, 2006).

### 5.2 Competing hypothesis and model fit comparison

Researchers may want to compare competing multivariate descriptions of a conceptual model (based on a priori hypotheses), but formal ways to compare such models and identify whether they are sufficient descriptions in the first place have rarely been considered. In SEM, we obtain one overall model fit and can compare model fits between models, which makes it possible to learn about a system. Various metrics of model fit exist for SEM, but we focus here on the comparative fit index (CFI; Bentler, 1990) that is mostly reported in ecological studies (see Table S7 for other metrics). CFI is a chi‐square estimate using a maximum‐likelihood solution, where values ≥0.90 and ≥0.95 indicate an acceptable and reasonably good fit, respectively (Hooper et al., 2008; Hu & Bentler, 1999; Lefcheck, 2019). We constructed an alternative model to compare with the original one (Figure 5) to explore whether we can improve the model. In the original model, by design, all effects of body size go indirectly through condition, whereas by adding a direct pathway of a latent construct ‘body size’ on survival, we can learn the relative role of body size and body condition (which is often understood as being independent; Schulte‐Hostedde et al., 2005). Both models show good model fit with a CFI of 0.94 (Codes S6 and S7). The alternative model reveals no direct effect of body size on survival, but only an indirect effect through mass and condition (Figure S13). Comparing both models with an ANOVA confirms that the alternative model (with an additional direct effect of body size on survival) does not show a significant improvement (*p* = 0.4; Table S8) compared to the original model. We have thus learned that effects of body size primarily affect survival through its effect on body condition. This model comparison also illustrates that if no direct arrow connects two variables, independence between variables is assumed (e.g. between size or sex and survival; Figure 5). The decision on which pathways to include in a model or not, should be based on prior biological knowledge and the aim of the study, and thus requires careful thinking about hypotheses.

### 5.3 Model performance of SEM versus Conventional approach

**FIGURE 6:**
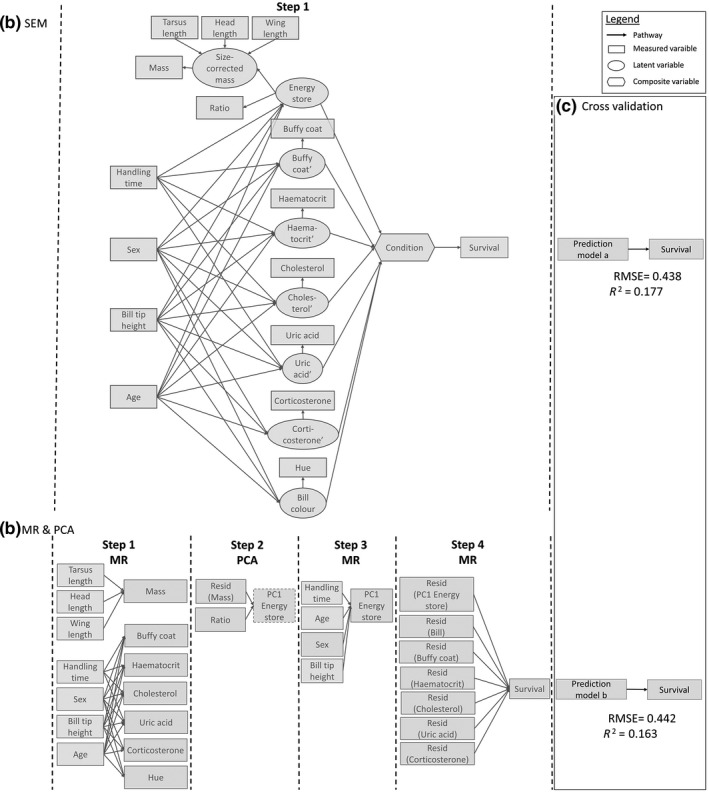
Comparison of two analysis approaches using (a) structural equation modelling (SEM) and (b) conventional methods: multiple regression (MR) and principal component analysis (PCA). Arrows point from predictor to response variable within the model. (a) SEM to quantify body condition in oystercatcher that is positively related to survival including latent and composite variables in one step. (b) MR&PCA approach to quantify body condition which needs 4 steps (compared to 1 step in SEM). (c) shows the root mean square error (RMSE) and *R* ^2^ for both models as a result from cross validation. Note that the variable ‘competitor density’ (from Figure 5) was not included in the SEM model to compare the model performance of both approaches (a and b) since the aim was to create a proxy of body condition that best explains the variation in survival and therefore predicts the survival well with a train and test dataset (cross validation). See Code S8 and Table S9 for model specifications and R code as well as the details of each step of the MR&PCA approach, respectively

In the conventional approach, we can imitate latent variables using PCA and composite variable using residuals from MR (Figure 6a vs. 6b). However, using conventional methods (MR and PCA) to create a condition index that predicts survival requires a large number of steps, especially when multiple condition variables are measured (Figure 6b). We expected the SEM to perform better than the conventional approach, as in SEM we can estimate the joint likelihood of all the relations simultaneously (Figure 6a), contrasting with the four steps in the conventional approach (Figure 6b). The conventional approach involves using the residuals from a previous model in input in the actual model, which may cause bias in parameter estimation (Darlington & Smulders, 2001; Freckleton, 2002).

We compared how well the body condition index could predict survival using either the SEM or the conventional method (Figure 6). Model performance was quantified using a measure of prediction error (root mean square error; lower values imply less deviations between model predictions and observed survival estimates) and a measure of explanatory power (*R* ^2^; higher values indicating more variation explained). We applied 10‐fold cross‐validation to the original dataset, with 80% of the dataset being used as training dataset to which the SEM (Figure 6a) and conventional model (Figure 6b) were fitted and 20% being used as test dataset to assess model performance of both methods. This process was repeated three times, meaning that in total 30 different training and test sets were used to estimate the performance (see Codes S8 and S9). The results of the cross validation show that the SEM approach has higher model performance which is indicated by a 1% reduction of the prediction error (RMSE) and an increase of the explained variation (*R* ^2^) by 9% compared to the conventional (MR/PCA) approach (SEM: RMSE = 0.438; *R* ^2^ = 0.177; MR&PCA approach: RMSE = 0.442; *R* ^2^ = 0.163; Figure 6c).

In addition, we compared the bias and precision of estimators from both approaches. To this end, we simulated 1,000 datasets with 1,000 observations each, based on the covariance patterns found in the case study population using the ‘simulateData’ function in lavaan (Rosseel, 2012). Next, we estimated the effect of each condition variable on condition index using either a SEM (Figure 6a; Figure S14) or conventional stepwise MR&PCA (Figure 6b; Figure S15) approach. For each condition variable of both approaches, we calculated the mean bias ($\hat{B}=\frac{1}{n_{\text{sim}}}\sum_{i=1}^{n_{\text{sim}}}\hat{\theta_{i}}‐\theta$) and the average empirical standard error ($\hat{E}=\sqrt{\frac{1}{n_{\text{sim}}‐1}\sum_{i=1}^{n_{\text{sim}}}\left(\hat{\theta_{i}}‐{\bar{\theta }}^{2}\right)}$) of the estimate, with *θ* being the parameter value used to simulate the data (‘true value’), ${\hat{\theta }}_{i}$ being the estimated parameter from the simulated *i*th repetition, $\bar{\theta }$ being the mean of the estimated parameters and *n*_sim_ being the number of simulated datasets.

**FIGURE 7:**
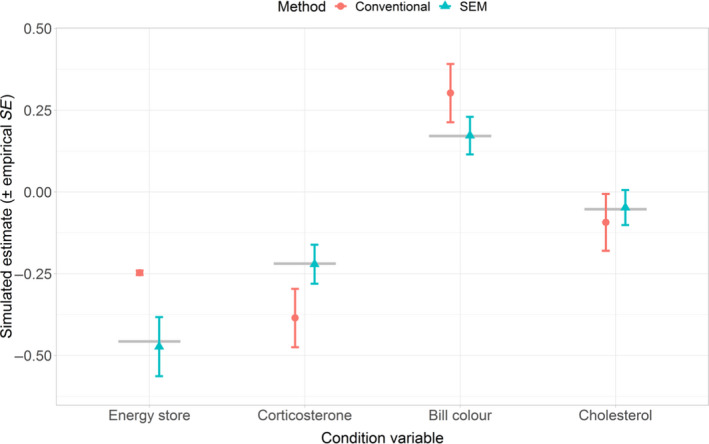
Results from the simulation comparing biases in the estimation of the slopes of the condition variables using the SEM and the conventional approach. Dots and triangles indicate the mean simulated estimate for the conventional method and SEM, respectively, with the empirical standard error. Grey horizontal line indicates the ‘true’ values used to generate the data. Note that standard error of the variable ‘energy store’ is really small and error bars are therefore hardly visible. Here we illustrate effect sizes estimates for the four condition variables that contributed most strongly to the condition index, see Figure S17 for the bias of all seven condition variables

Simulation results indicate that the SEM method is practically unbiased while the conventional method can have substantial bias and not consistently in the same direction (Figure 7). For condition variables having a ‘true’ value not close to 0, we calculated the relative bias (Morris et al., 2019) which ranged from −85% to 50% and 0.8% to 3.5% for the conventional approach and SEM, respectively (Figure S16). The bias of the conventional estimators is largest in the variables having the strongest (significant) effect on the condition index (Figure 7; Figure S17), ranging from −0.16 to +0.21 absolute bias (Figure S18). Overall, the estimator of the SEM approach was also more precise (with an empirical standard error around 0.056) than the conventional approach estimator (with an empirical standard error around 0.087), when considering estimators that were unbiased in both methods (e.g. uric acid; Figures S19 and S20).

## 6 DISCUSSION

We showed how to use structural equation modelling (SEM) to quantify body condition, comparing it with the conventional approaches of multiple regression and/or principal component analysis. We illustrated how latent variables can be used to summarize the multidimensional nature of body condition, yielding variable reduction. Next, we showed that composite variables make it possible to define the body condition index based on associations between observed condition variables and a fitness trait, even if the different condition variables contributing to body condition are uncorrelated (potentially reflecting different axes of condition). This is advantageous, because the direction of a relationship between a condition measure (e.g. body mass) and fitness is often assumed, but rarely known with certainty. Finally, we showed how it is possible in SEM to combine latent and composite variables in one model, which more realistically reflects complex natural situations, as it allows for summarizing both strongly correlated and less correlated measures of condition. A major advantage of SEM is that, in contrast to conventional methods, it can handle variables that are response and explanatory variables at the same time. As a consequence, SEM allows all the relationships to be estimated in a single model, resulting in one estimate for the model fit and generating the possibility to compare competing models, whereas in conventional methods several steps are required (e.g. extracting residuals of a model and use them as input for another model) that provide additional challenges (e.g. biased parameter estimates and carrying over the uncertainty from previous models). In addition, we showed how we can correct for the effect of confounding variables in SEM using MIMIC (multiple‐indicator, multiple‐cause) modelling. Overall, the high flexibility and integration of SEM make it a powerful tool that might increase model predictability and result in unbiased and precise estimates, as illustrated by our worked examples on a case study, and therefore may also provide novel insights in ecological processes.

While we used SEM to quantify body condition, there are numerous other contexts in which it can be used to describe complex concepts. For example, in determining an immune response or experienced stress level of an individual, because usually multiple immunological, physiological or hormonal metrics are measured, which may also reflect different aspects of the immune or stress response, and it is a challenge to derive an integrated immune estimate from these data. Similarly, climatic conditions are highly multivariate, and while most studies have, for example, detailed climatic data on various weather variables, studies tend to focus on a single weather variable such as temperature.

### 6.1 From a biological point of view: Body condition related to different performance metrics

A condition index based on predicting survival does not necessarily mean that a high body condition of an individual indicates high fitness, because fitness also depends on the association between condition and other fitness components, such as reproductive success. Quantifying condition based on survival data may result in a different condition index than when it is based on the reproductive success of an individual, or a fitness metric that integrates survival and reproduction, such as reproductive value. This difference highlights that definitions of condition can be context‐dependent and may vary during the life cycle, which may be particularly relevant when investigating species that alternate strategies (e.g. albatrosses prioritizing reproduction in one year and survival in another year; Froy et al., 2013). Our SEM approach provides a framework for identifying how different condition measures define a successful survivor or a successful breeder and how they may differ from each other.

### 6.2 From a statistical point of view: Additional techniques, challenges and limitations

We want to emphasize that in order to compare the two approaches, the data used to analyse the data and generating the data had the same model structure. However, in reality, this will rarely be the case. Different research questions require different statistical frameworks (model structures) and a thorough understanding of the definitions and components of the frameworks as well as of the study system is crucial for making biologically meaningful inferences (Benthem et al., 2017). Therefore, identifying a proper model structure (that fits the data well) and that addresses a biological question properly is by no means trivial and probably one of the largest challenges in the art of modelling.

We considered confounding variables (e.g. sex and age) to illustrate how it can be modelled in SEM in general, but there may be many more confounding variables, and most will not have been measured (e.g. social dominance on foraging grounds). The presence of unmeasured confounders is a general challenge in observational studies (VanderWeele & Arah, 2011), but sensitivity analysis and bias‐modelling techniques can help handling uncontrolled confounding variables (Lin et al., 1998; McCandless et al., 2007).

In this paper, we used latent variables to achieve variable reduction (similar to PCA). However, latent variables can also be used to account for measurement errors of observed variables. Conventional statistical approaches like MR assume that variables are measured without error (Pugesek & Tomer, 1995) even though it is known that it is usually impossible to measure variables without error, particularly in the field (Musil et al., 1998). Ignoring measurement errors typically leads to downward bias in parameters because an error in measuring *X* (explanatory variable) is assigned to the error in predicting *Y* (response variable), implying that the true effect of *X* on *Y* is typically underestimated (i.e. regression dilution/attenuation). Pugesek and Tomer (1995) showed that SEM, by including measuring errors, estimated parameter coefficients more accurately and with less bias compared to MR. Code S10 shows an example in which we account for imperfect measurements (through latent variables) of mass regressed on body size structures (tarsus, head and wing length).

SEM statistical packages are under continuous development, but particularly frequentist statistical approaches based on maximum likelihood estimation still have limitations. Random effects in combination with latent variables are currently challenging to model with one of the most widely used r‐packages, lavaan (Rosseel, 2012). In the r‐package piecewisesem (Lefcheck, 2016), it is possible to model random effects when conducting SEM to identify direct and indirect pathways, but it is currently impossible to model latent variables with this package.

For categorical predictor variables, a multi‐group analysis can be conducted. Multi‐group analysis can also be an alternative way to account for random factors with few levels. The question that drives a multi‐group analysis is whether two or more groups might differ in terms of the relationships among parameters and the whole model is run for each level (Grace, 2006). To illustrate the use of categorical variables, we used the age effect in the example of Figure 2b as a category (Code S2), which means that the model calculates the relationships (Figure 2d) for each age class separately. In addition, multi‐group analysis can also be conducted to test for interactions (with a categorical variable). However, a multi‐group analysis together with a binomial response variable is not supported yet in lavaan which raises practical challenges analysing 0/1 survival data using logistic regression in combination with categorical variables, such as area‐specific resighting probability which were relevant in our case study. An example of R‐code for modelling a binomial response variable but without multiple levels (only one area) is presented in Code S11.

Nonlinear relationships are common in ecology and there are different ways to address nonlinearity. The easiest way is through transformation, as we did in our case study, applying logit‐transformation for proportional response variables and squaring mass (Code S4). Multi‐group analysis can also be used to address a certain type of nonlinear relation (Grace, 2006). Continuous nonlinear relationships are a special challenge for SEM, both for observed but especially for latent variables (Grace, 2006). A useful introduction to this topic is chapter 12 in Kline (2011).

Bayesian approaches to SEM may offer solutions to limitations of frequentist software in dealing with random effects, non‐Gaussian data and multiple‐level categorical predictors, because Bayesian software uses more convenient numerical algorithms. Furthermore, a Bayesian approach allows for constraining estimates using informative priors, so as to include biological knowledge. Bayesian framework has also been used to combine a mark–recapture model with SEM. Cubaynes et al. (2012) show an example of how to model a latent variable (‘overall body size’) that affects survival estimated within a mark–recapture framework (all in one model). Possible r‐packages that can be used for such Bayesian approaches are rstan (Stan Development Team, 2020), jagsui (Kellner, 2019) and nimble (de Valpine et al., 2017).

To conclude, we show that SEM is a powerful and flexible statistical tool that can lead to models of higher predictive power and with more accurate as well as precise estimates compared to conventional approaches. Therefore, we encourage researchers to consider SEM as a flexible framework to describe the multivariate nature of body condition and thus understand how it affects biological processes, thereby improving the value of body condition proxies for predicting organismal performance. We emphasize that SEM can also be a useful tool for other multidimensional ecological concepts as well, such as immunocompetence, oxidative stress and environmental conditions.

## CONFLICT OF INTEREST

All authors declare no conflict of interest.

## AUTHORS' CONTRIBUTIONS

M.F. and M.v.d.P. conceptualized the research, with A.M.A., B.J.E., E.J., H.d.K., H.‐J.v.d.K., J.N. and S.V. contributed to its development; M.F. and H.‐J.v.d.K. managed the field data; M.F. performed the analysis and drafted the manuscript. All authors contributed to the final draft and approved the final manuscript.

## Supporting information

Supplementary Material

## Data Availability

Data files and R code to fit the models and simulations are available on GitHub and can be accessed via https://github.com/MagaliFr/QuantifyingBodyConditionWithSEM or https://doi.org/10.5281/zenodo.5153493 (Frauendorf et al., 2021).
