# Supplementary material for: Conceptualizing and quantifying body condition using structural equation modelling: A user guide
Source: J Anim Ecol. 2021 Sep 6;90(11):2478–96. doi: 10.1111/1365-2656.13578 (PMC9291099; doi:10.1111/1365-2656.13578)
Supplement: Supplementary file 1 — Supplementary Material [file JANE-90-2478-s001.docx]

Supporting Information to

**Conceptualizing and quantifying body condition
using structural equation modelling: A user guide**

Frauendorf et al.

25-08-2021

Contents

[Text 2](#_Toc81387588)

[Figures 5](#_Toc81387589)

[Tables 22](#_Toc81387590)

[Codes 29](#_Toc81387591)

[References 56](#_Toc81387592)

# Text

*Text S1: Choice, description and data collection of biometric and physiological measures included in the worked examples*

*Blood samples (sex, haematocrit, buffy coat, cholesterol, uric acid)*

Buffy coat, the fraction of white blood cells, is elevated if the body needs to fight against infections (Campbell et al., 2008). Haematocrit, the proportion of red blood cells with the main function of oxygen transportation, has been shown to be positively related to survival in Eurasian oystercatcher (*Haematopus ostralegus*) (Verhulst et al., 2004) and crimson fiches (*Neochmia phaeton*) (Milenkaya et al., 2015). Uric acid is a nitrogenous waste product of birds (Tsahar et al., 2006). The amount of nitrogenous waste produced by an individual can be linked to energy budget and diet (Campbell et al., 2008) and has been shown to be related to body mass loss in Yellow-legged gulls (*Larus cachinnans*) (Alonso-Alvarez et al., 2002). Cholesterol, a lipid, has been shown to best reflect body mass change in Yellow-legged gulls (*Larus cachinnans*) (Alonso-Alvarez et al., 2002). Griminger (1986) also suggested an influence of diet composition on cholesterol levels in birds.

Blood samples (approximately 0.35 ml per bird) were taken from the brachial vein. Sex was determined using molecular techniques from blood stored in cell lysis buffer at room temperature.

Two blood collection tubes of approximately 65 µL each were taken per individual bird and centrifuged 10 minutes at 9503g between 2 to 6 hours (mean$\pm$se=3.6$\pm$0.96hrs) after blood extraction. Since this time delay did not significantly affect buffy coat and haematocrit, it was not included as a variable in the analysis. Buffy coat and haematocrit were measured by taking standardized pictures of the blood collection tube in a specific holder constructed for this purpose (Fig. S21), and then measuring the length of the portion with red blood cells, white blood cells and plasma in pixels with the program Paint.NET. The haematocrit and buffy coat were calculated by taking the proportion of the length of red blood cells and white blood cells, respectively, to the total length (red blood cells + white blood cells + plasma). The reason for using pictures and measuring pixels instead of measuring proportions directly in the field was to minimize measuring bias due to more accurate measurements compared to a ruler/calliper. Repeatability (calculated by estimating the intra-class correlation coefficients (ICC) (and its 95% confidence interval) using the variance components from a one-way ANOVA with the r-package ICC (Wolak et al., 2012) between the two collection tubes of the same individual of both haematocrit and buffy coat samples was high. Haematocrit: ICC (lower & upper confidence interval) = 0.984 (0.982, 0.986), n=758 duplicate samples. Buffy coat: ICC (lower & upper confidence interval) = 0.943 (0.931, 0.953), n = 390 duplicate samples). Number of observations for the buffy coat was smaller because samples were removed when edges were not sharp and/or diagonal.

The blood of two capillaries was transferred to tubes with heparin buffer and centrifuged at 6082g for 8 minutes. After centrifugation, the plasma (at least 50 µL) was extracted and stored in a -80°C freezer. Cholesterol (mmol/L blood) and uric acid (µmol/L blood) was determined (cholesterol: enzymatic method on discrete automatic analyser; uric acid: automatic and colorimetric method using uricase) by the University Veterinary Diagnostic Laboratory (UVDL) in Utrecht, the Netherlands, with Olympus AU-680 from the Beckman Coulter company.

*Biometry*

Biometric parameters, namely length of tarsus to toe (mm), wing length (mm), head length (mm), bill tip height (mm) and mass, were measured following the standard techniques described in Durell et al. (1993). Bill tip height (measured 3 mm from the bill tip using a calliper) was used as a proxy for the type of individuals’ feeding specialization (individual characteristics; Fig. 1), ranging from worm specialists (indicated by a pointed bill and a low bill tip height) to shellfish specialists (indicated by a blunt bill and high bill tip height) (Van de Pol et al., 2009). Birds were aged on basis of their plumage, bill and leg characteristics (Cramp & Simmons, 1983) and the age was classified in 1^st^, 2^nd^, 3^rd^ calendar year and adults (>3^rd^ calendar year). We focused only on sub-adults (2^nd^ and 3^rd^ calendar year) and adults (>3^rd^ calendar year) in the case study because number of caught and sampled juveniles (1^st^ calendar year) was small (n=28; Table S1). Handling time (confounding variable; Fig. 1) was recorded to correct for time-dependent mass loss as well as possible effects on other physiological measures. Handling time (as a proportion of 24 hours) was defined as the time between capture and measuring (Fig. 1) to correct for seasonal effects on energy reserves and physiological parameters (Norte et al., 2009).

*Feather sample (corticosterone)*

Corticosterone, a steroid stress hormone, fulfils its main functions by mobilizing stored resources and up-regulating metabolism for coping with increased energetic challenges, resulting in higher corticosterone secretion in birds coping with harsh environments (Jimeno, Briga, et al., 2017; Jimeno et al., 2018; Jimeno, Hau, et al., 2017; Marra & Holberton, 1998).

When a bird was caught, we took the 5^th^ tail/flight feather from the left side of the bird and stored it in a paper envelope until it was sent to the laboratory of the Department of Evolutionary Ecology at the National Museum for natural Science (CSIS) in Spain for corticosterone extraction. We followed the methodology for steroid extraction from feathers described in Bortolotti *et al.* (2008). Feather samples were prepared by selecting the most proximal part of the feather, next to the calamus, which was least abraded, and cutting around 3.5 cm from there, taking white feather only. Feather samples were weighed to the nearest mg with an analytical scale (Sartorius). Average mass of feather material per sample was 32.4 mg (SD = 4.24). The vane plus rachis were cut in small pieces (<5 mm) with scissors. We added 6 ml of methanol to the tube with the feather particles, and left the tubes for 30 min in an ultrasound water bath. Then, tubes were capped and left overnight (around 19 hours) in a shaking water bath at 50°C. Samples were decanted in a clean tube and filtered using a nylon plug filter (0.45µ). Tubes were washed with 2 ml of methanol, which was added to the previous extract after a similar filtering. Samples were then placed in a heated tube rack (50°C) under a stream of nitrogen until evaporation (Techne, Germany). Dried extracts were suspended in 150 µl of steroid free buffer and vigorously vortexed for 10 min. Extracted samples were then assayed following kit inserts using a commercial corticosterone ELISAs (DRG, Germany), and optical density measured with a plate spectrophotometer (BioTek, USA).

*Bill colour*

Oystercatchers have orange bills, and presumably this is due to carotenoids. Carotenoids have been linked to antioxidant and immune status signalling (Perez-Rodriguez, 2009; Simons, Cohen, et al., 2012; Von Schantz et al., 1999) and may therefore be a signal of individuals’ phenotypic quality. In male zebra finches, for instance, bill redness reflects recent environmental (Eraud et al., 2007) and immunological challenges (Cote et al., 2009), and has been shown to be positively correlated to immune functioning (Birkhead et al., 2006) and survival and reproduction (Simons, Briga, et al., 2012).

Bill colour measurements were performed using digital photography (Panasonic Lumix GX8) from each individuals’ right and left side, which has been shown to be a valid method in ecology to study animal coloration (Simons, Briga, et al., 2012; Stevens et al., 2007; Villafuerte & Negro, 1998). The colour of an object is greatly influenced by the colour and brightness of the light source used to illuminate it. To minimise variation in light colour and brightness, photographs were taken in a purpose-built photo-box to ensure standardized light conditions (Fig. S22). To further compensate for variations in light source colour and brightness and allow captured colour and brightness information to be standardised in order to compare different pictures from different individuals, the colours white, grey and black were visible from a photo reference card on each picture (Fig. S22). This allowed post-production colour and exposure balancing of the images. Camera settings were also standardized for all pictures. We used the program, Pixel Grabber (Nienhuis, 2015) (Fig. S22) to quantify the colours in a continuous scale of the conventional colour model developed for humans, amenable for statistical analyses. Colour measurements may be defined according to different systems (e.g. Munsell, Lab, HCL) but one of the most widely used is the HCL colour system, which provides independent values of hue, chroma and luminance which are the parameters generally used to define a colour. Hue corresponds to wavelength of light, chroma refers to spectral variance and therefore to colour purity so that the more monochromatic a colour is, the higher its chroma value. Luminance is correlated with physical light intensity and refers to the position on a grey-scale between black and white (Quesada & Senar, 2006).

This gives for each part of the bill measured for each individual three axes of the colour: Hue, Chroma and Luminance, where a higher “hue” indicates a more yellowish colour compared to a more orange colour (Fig. S7). Pictures where the measuring points were damaged, dirty or blurry (3%) were excluded from analysis. Correlations of the right and left side of the bill as well as different parts of bill were correlated (Table S10), so that we focused on only one of the several bill measuring points for analysis.

For a random subset (n=86) of the total dataset (n=598), we repeated the measurement of the bill colour parameters (hue, chroma and luminance) to get an indication of the repeatability of the measurements. Repeatability was calculated by estimating the intra-class correlation coefficients (and its 95% confidence interval) using the variance components from a one-way ANOVA with the R-package ICC (Wolak et al., 2012). The results indicate high repeatability in the measurements (hue: 0.95 (0.92, 0.96); chroma: 0.94 (0.91, 0.96); luminance: 0.95 (0.92, 0.97)).

*Survival estimates*

We applied a multi-state live and dead recoveries model (MSLD) using RMark (Laake, 2013) with some adjustments to the model as described in Allen et al. (2019). An age effect was included in order to calculate survival estimates for different age classes: juveniles (1 year-old individuals), sub-adults (2 -and 3-year-old individuals) and adults (being older than 3 years; Fig. S23). Separating 2 -and 3-year-old birds in the analysis was not possible because of sample size issues. To improve parameter identifiability, we expanded the sampling period to include all oystercatchers ringed and observed between 2016 and 2019. This enabled estimates of seasonal survival for the different geographical states defined for our study area. We used seasonal (winter to summer) survival estimates, for the sampling period (of condition variables) between 2016 and 2018 (Fig. S23) for all analyses. The reason we use over-winter survival estimates is that the condition of the birds was measured in winter. The state (geographical location) of the individual was coded according to one of the nine geographical locations (Fig. S1): R = Rottum, S = Schiermonnikoog, T = Terschelling/Ameland, V = Texel/Vlieland, B = Balgzand/Texel, D = Delta, N = Inland North and P = Inland South. A state of A (Abroad) captured all observations outside the Netherlands (Allen *et al.*, 2019).

*Competition*

As environmental variable we used the density of oystercatchers in their wintering ground as a proxy for competition. Since 1975, high tide roost counts by volunteers are organized by the Dutch Centre for Field Ornithology (Sovon) in order to count waterbird species in the Dutch Wadden Sea and the Delta area (Hornman et al., 2012). These counts are done on a monthly basis throughout the winter.

We used the counts from the month November and January, since those were most complete for all catching locations. Within a buffer of 7km-radius around each catching location, we summed the numbers of counted oystercatchers for the month November and January separately, weighted for the counted area, because not the whole buffer area was counted. This resulted in oystercatcher density in number of birds per km^2^ for each month (November and January). Finally, we averaged the two densities to get a proxy of overall winter density. The 7km-radius buffer was chosen based on information of GPS-tagged oystercatcher movement around several high tide roosts in the Netherlands (Bakker et al., 2021).

# Figures


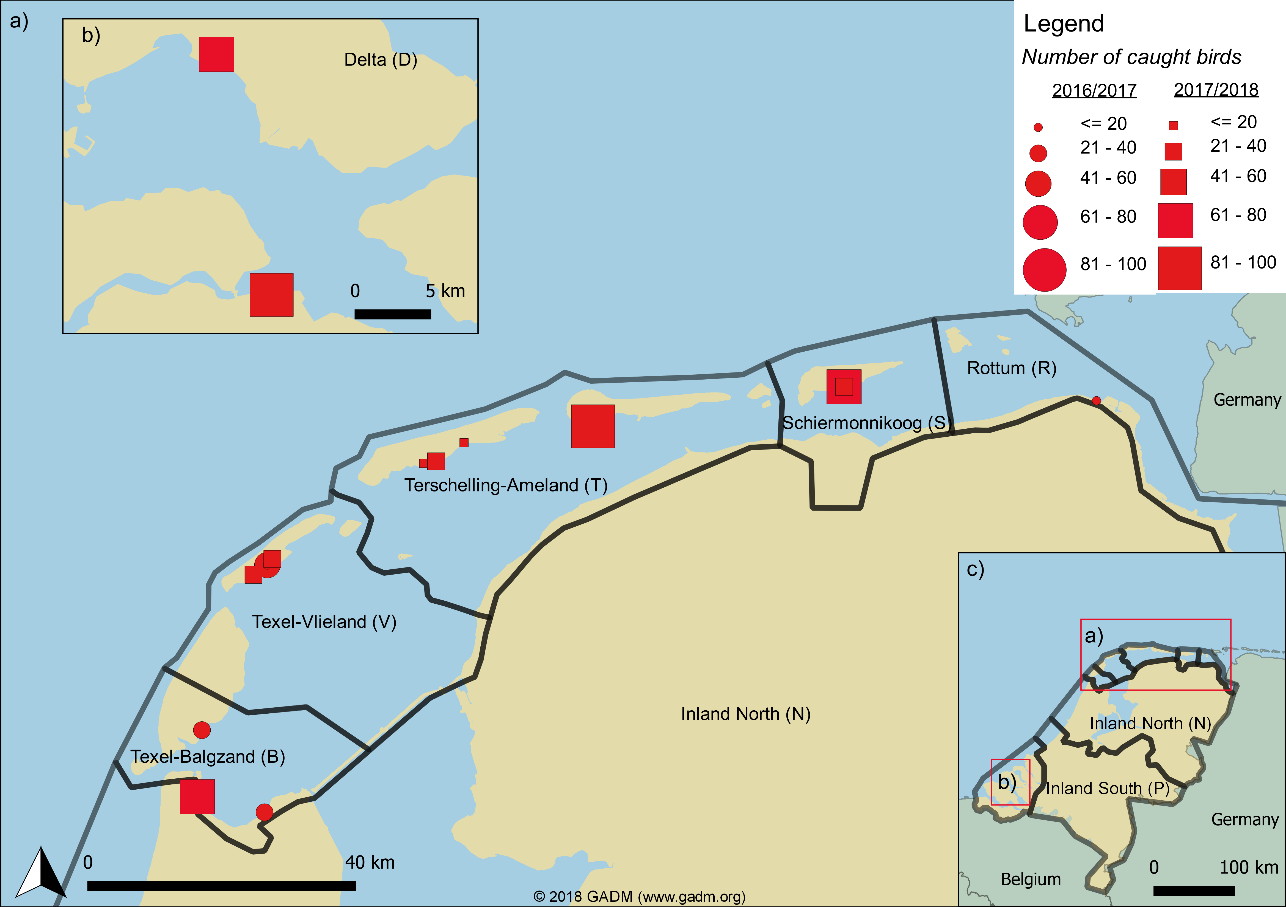


Figure S1: Study area in the Netherlands showing an overview of the catching locations across the Wadden Sea (a) and the Delta area (b) as well as the area-division used in the survival model. (c) indicates an overview map. Dots indicate catching events in the two winter periods (2016/2017 and 2017/2018). The size of the dots indicates the number of caught birds per catching event.


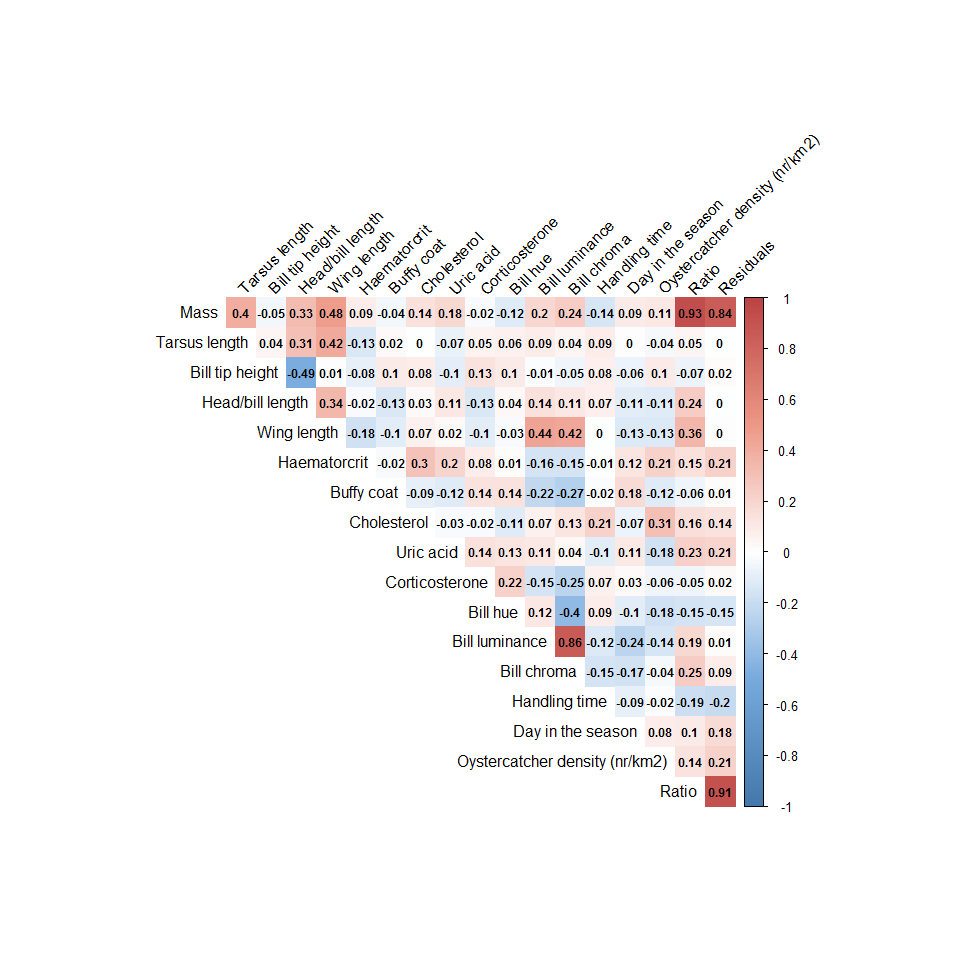


Figure S2: Correlation matrix of all variables in the dataset. N=430


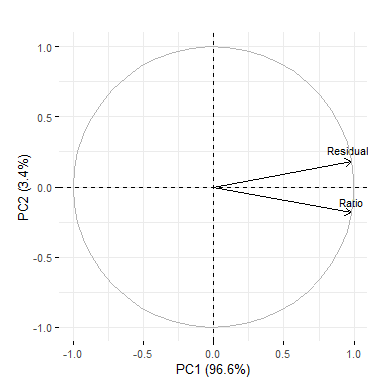


Figure S3: Visualization of the energy-reserve-PCA. PCA visualization was conducted with the R-package factoextra (Kassambara & Mundt, 2020).


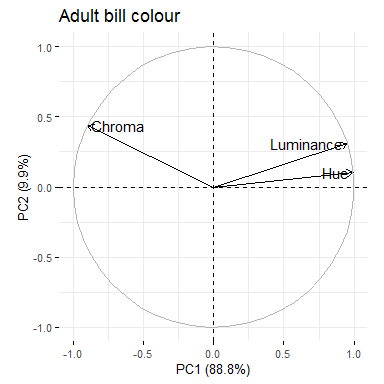


Figure S4: Bill colour PCA for adults. PCA visualization was conducted with the R-package factoextra (Kassambara & Mundt, 2020).


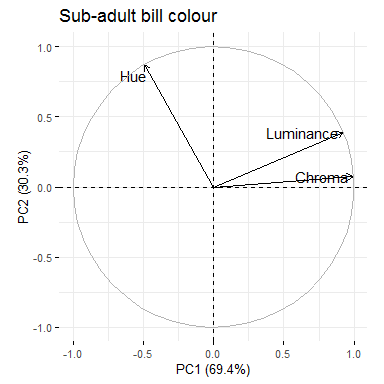


Figure S5: Bill colour PCA for sub-adults. PCA visualization was conducted with the R-package factoextra (Kassambara & Mundt, 2020).


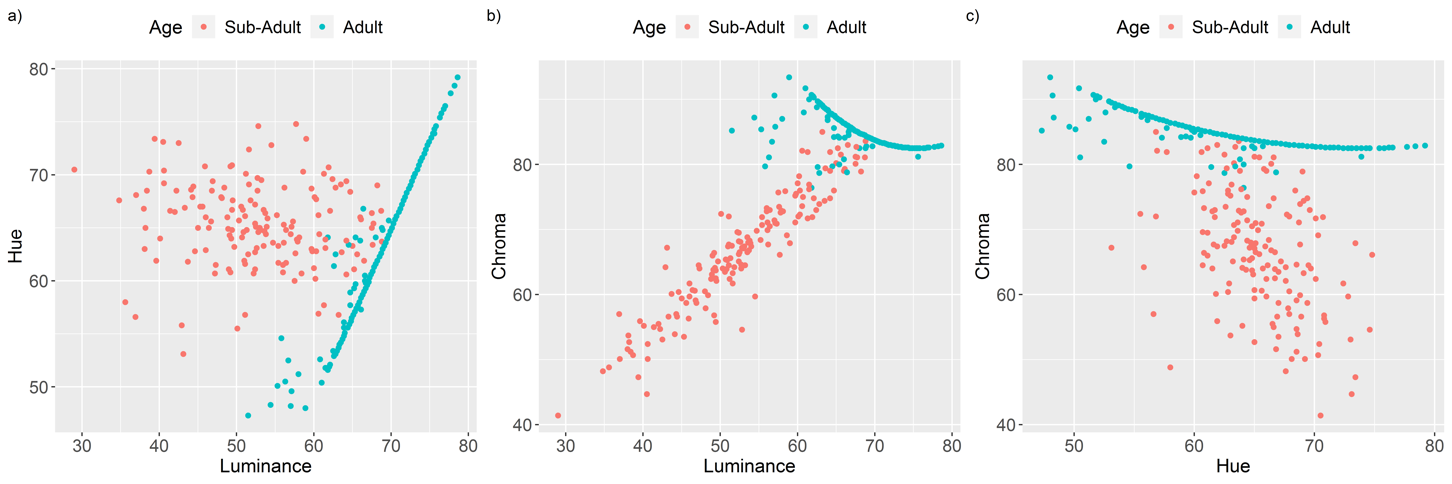


Figure S6: Relationships of the three colour parameters (hue, chroma and luminance) per age class. The relatively straight lines in all three panels in adults can be explained by the structure of the colour parameter chroma. Plotting the parameters in 3D results in a cylinder when using saturation (Fig. 26a), whereas, when plotting chroma instead of saturation it results in a biconical shape (Fig. 26b), which is also visible when plotting it two-dimensionally (Fig. S7).


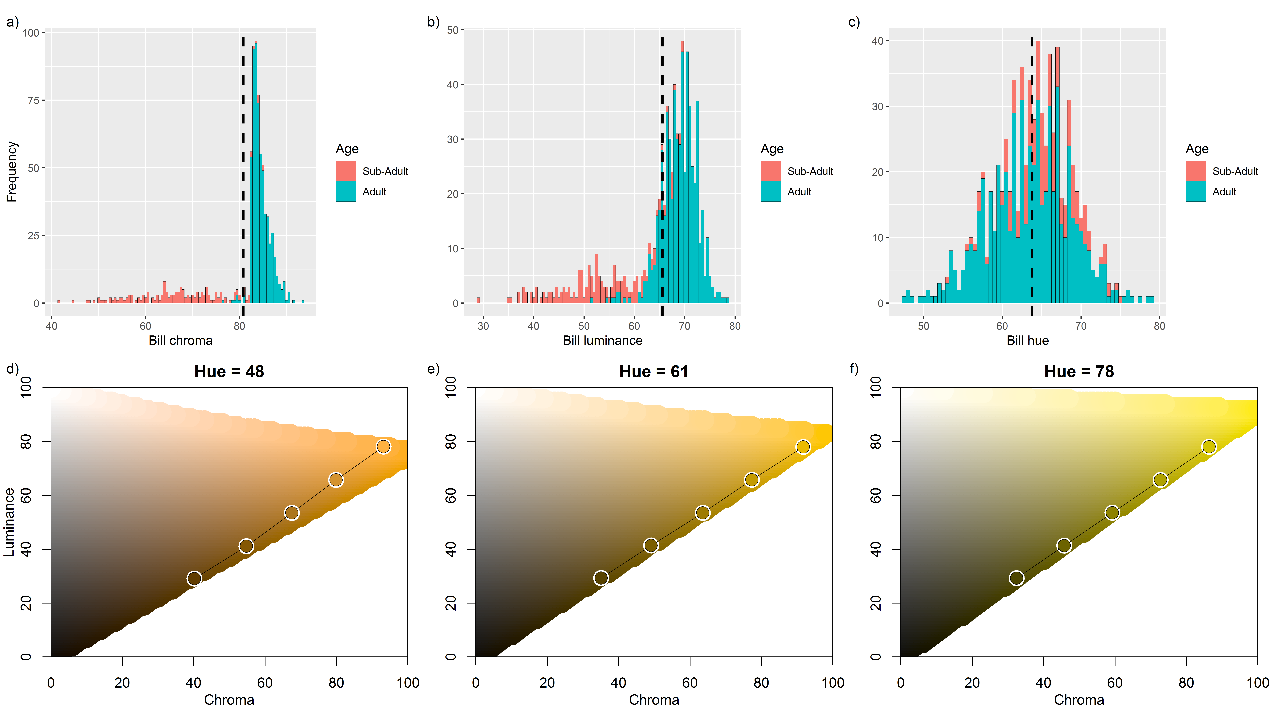


Figure S7: Distribution of bill chroma (a), luminance (b) and hue (c). d)-f) show bill colour range (circles) at changing luminance and chroma for minimum (d), mean (e) and maximum (f) hue values.


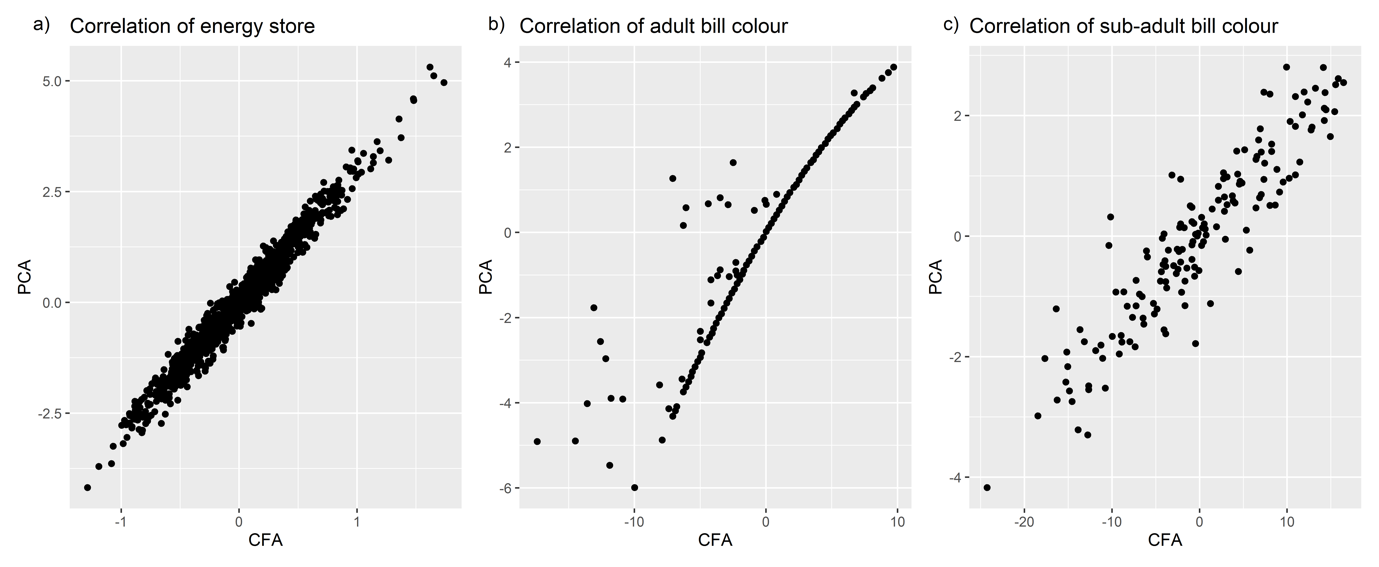


Figure S8: Correlation of extracted values from PCA (PC1) and CFA for the energy reserve model (a) and the bill colour model for adults (b) and sub-adults (c). For explanation of the straight line in b, see figure description S6.


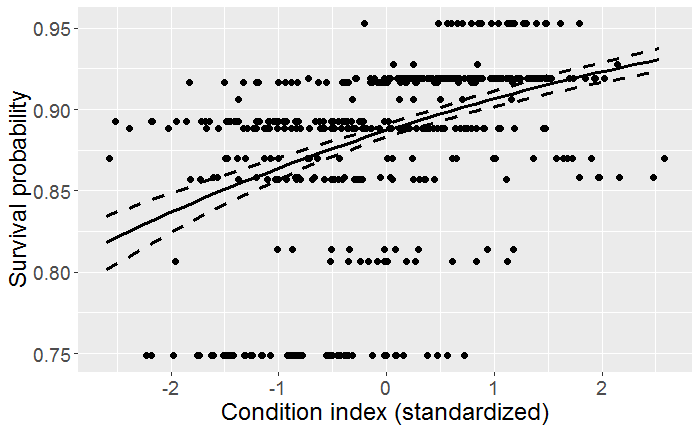


Figure S9: The relationship between the condition index and the survival probability from the SEM (Fig. 5). Condition is explaining 18% of the variation in survival. Note that several individuals have the same condition value because multiple individuals were caught at the same location and year resulting in the same survival probability from the multistate live and dead recoveries model.


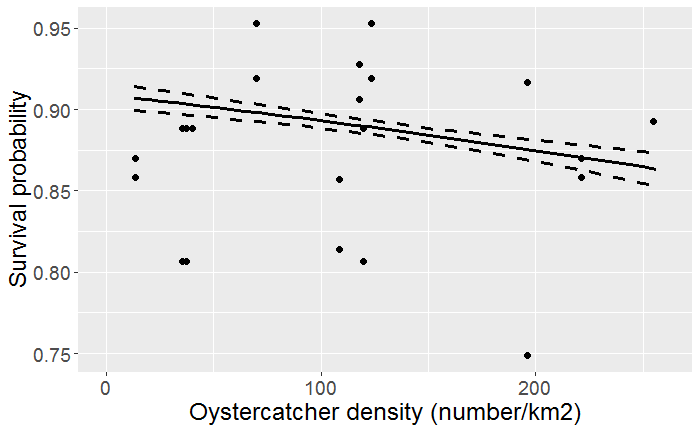


Figure S10: The effect of oystercatcher density on the survival probability. Each dot represents one wintering area and year combination.


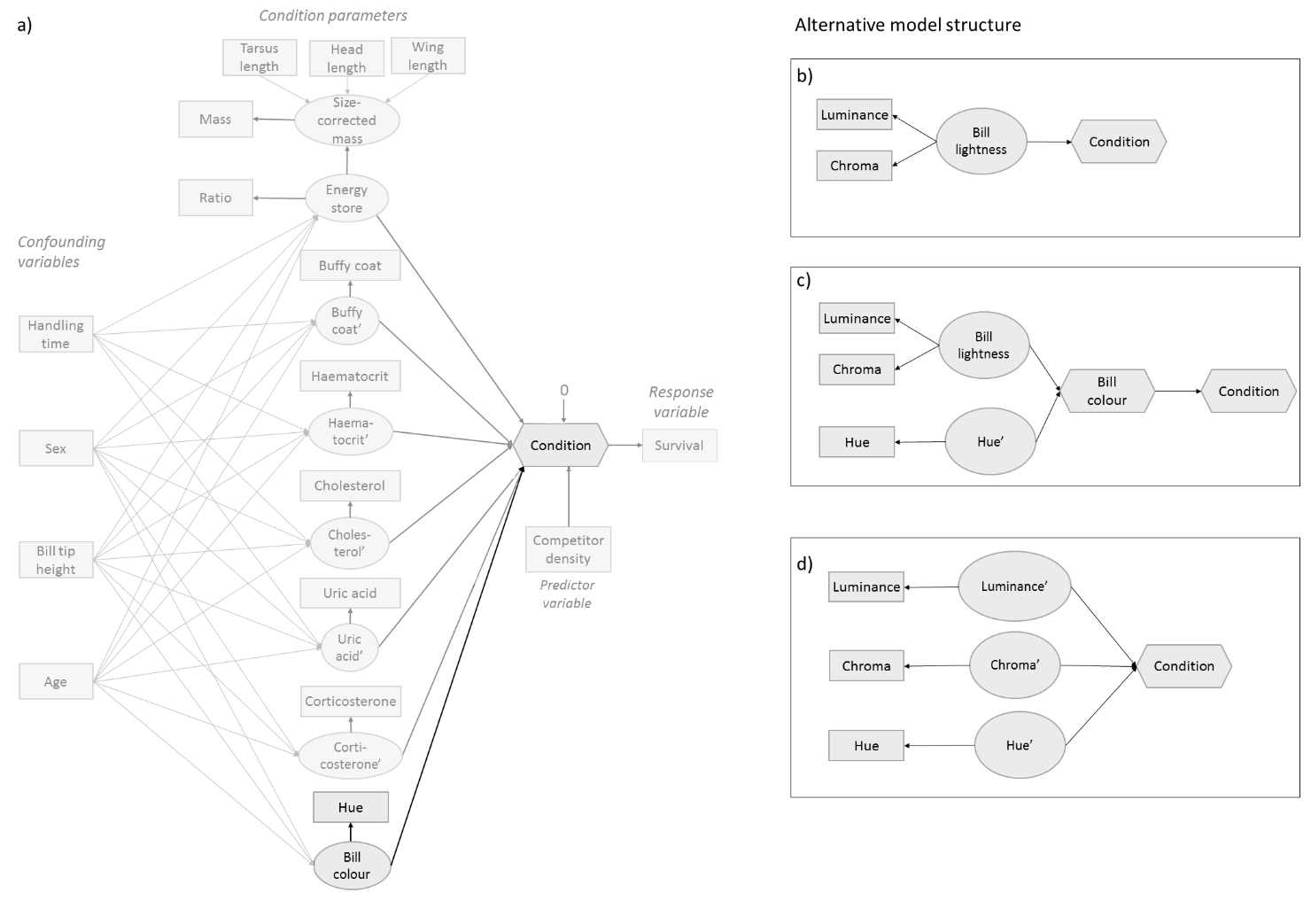


Figure S11: Illustration of the a) full model (Fig. 5) indicating how bill colour was modelled and alternative structures to model bill colour (b-d). b) shows a model structure where only bill lightness is modelled as latent variable by luminance and chroma, which are highly correlated in both age classes. c) shows an example to integrate luminance and chroma as latent variable (bill lightness) and hue as a separate variable (observed and latent) describing the composite (bill colour). d) shows an alternative structure where all three variables are modelled separately describing the composite condition. Note that we use latent variables (e.g. hue’) for hue to allow correction by confounding variables (e.g. sex) with the MIMIC method.


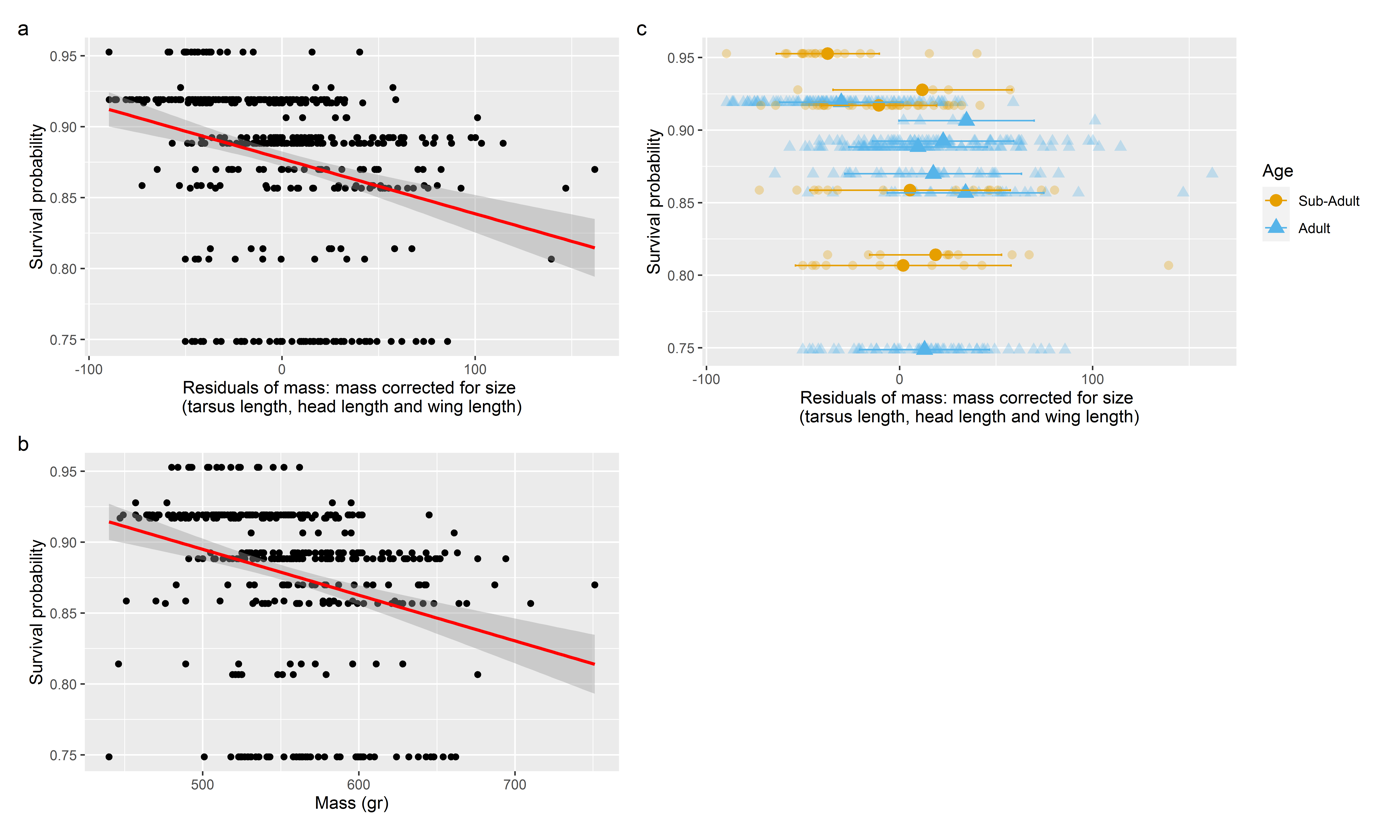


Figure S12: The effect of a) the residuals of mass (corrected for size) and b) mass (in gram) on the survival probability. Dots show the raw data points, the red line indicate the fitted line and the grey shaded area around it is the confidence interval. c) shows the effect of residuals of mass on survival per age class (blue triangle=adults, orange dot=sub-adults). The large symbol indicates the mean with the standard error (horizontal bars) and the raw data with more transparent fill in the background. Summary statistics for panel a) are: t-value=-7.62, DF=425, p<0.001.


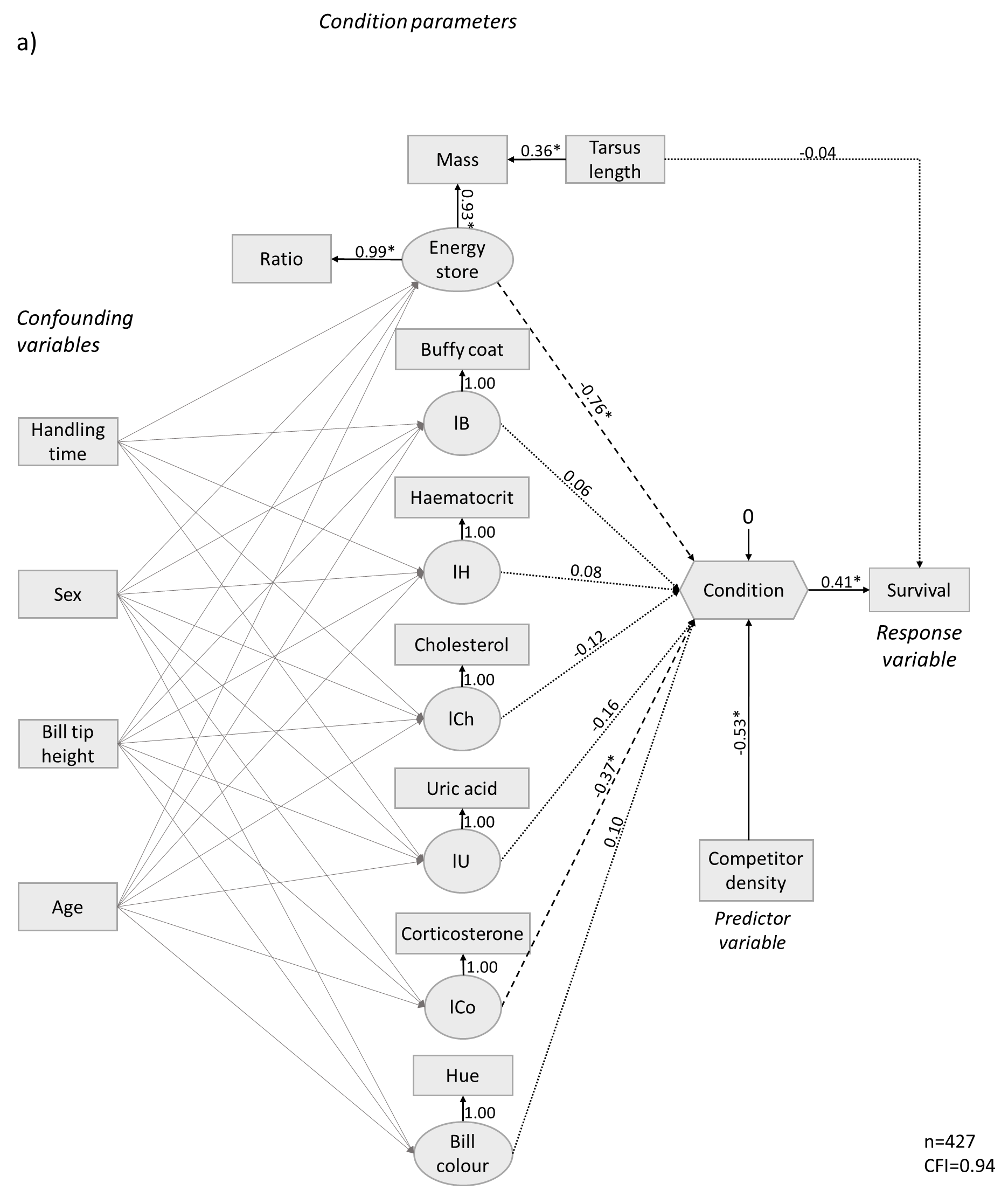

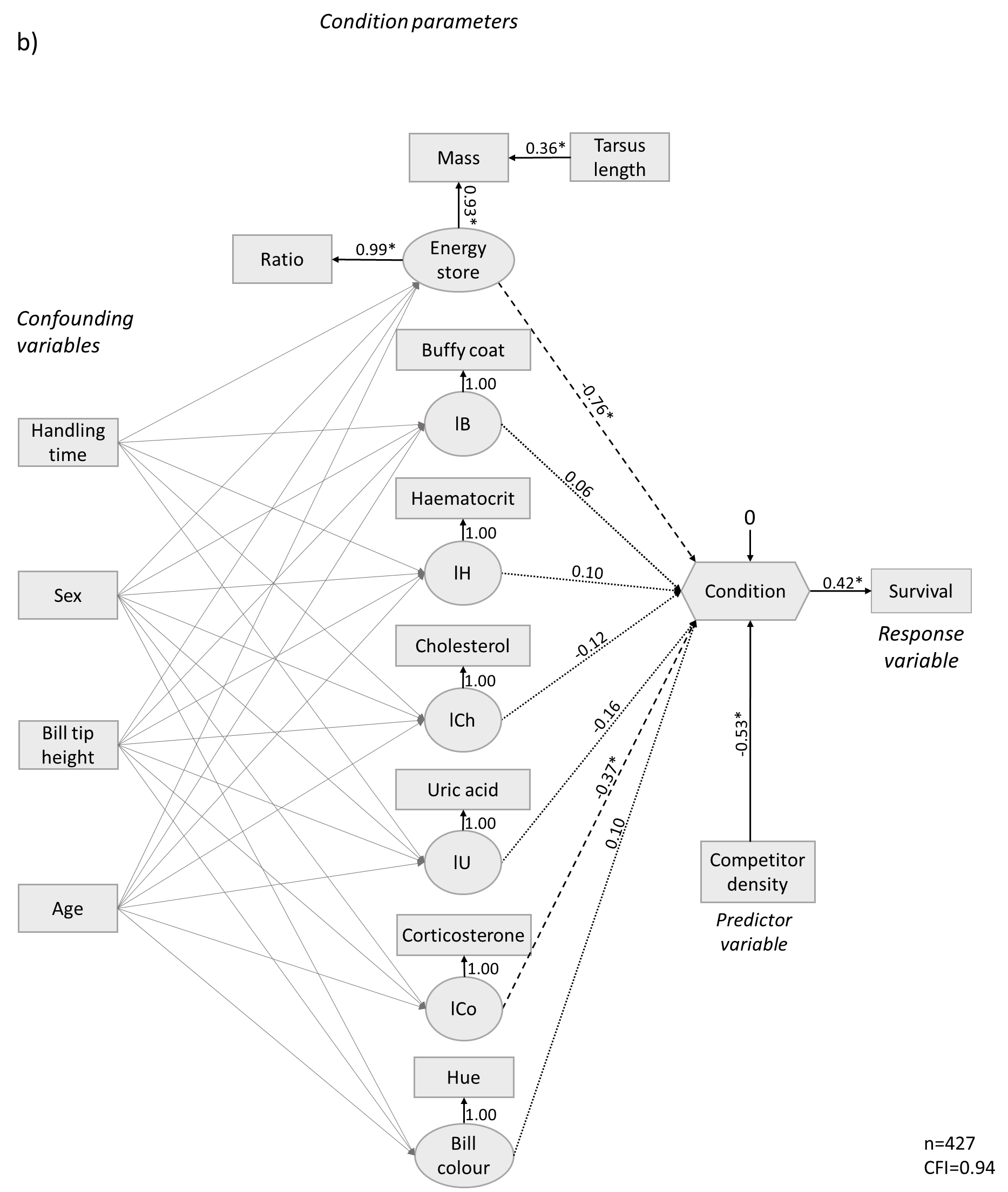


*Figure S13: SEM model including latent (circles), composite (hexagon) and measured (squares) variables (comparable to model of Fig. 5) with (a) and without (b) a direct effect of body size on survival which appears to be non-significant. Values indicate the standardized path strength (see Fig. 2 for explanation). Solid and dashed lines indicate a positive and negative significant (p<0.05) path and are indicated by a star next to the standardized path strength, respectively. Dotted lines indicate non-significant pathways (p>0.05). For simplicity we only documented the path strength of the condition, predictor and response variables. For the code of the models see Code S6-S7. Note that as body size measure we only used tarsus length because the three structural sizes (tarsus length, wing length, head length) are not highly correlated so that it is not useful combining them in a latent variable. Combining the variables in a composite variable is not an option because there is no observed response variable. We can compare the effect of the direct vs. the indirect pathway (through mass) of tarsus length on condition by taking the product of all pathways. This results in a total (standardized) effect of -0.04 of tarsus on survival directly vs. a total effect of -0.1 through mass, energy stores and condition.*


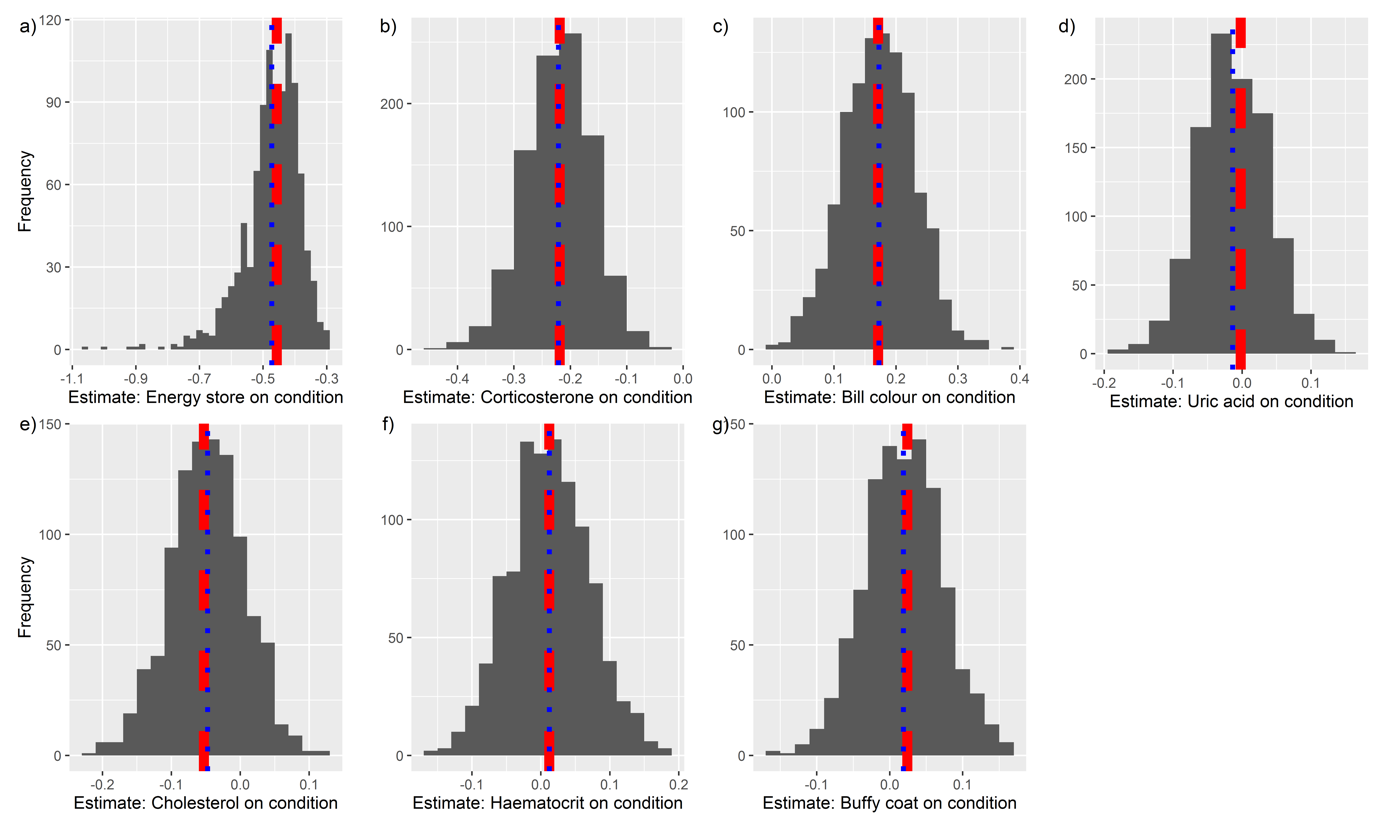


Figure 14: Histogram of the effect of each condition variable on condition (estimate) of the simulated datasets for the SEM-approach. Red dashed line indicates the ‘true’ mean, whereas the blue dotted line is the mean of the simulated data.


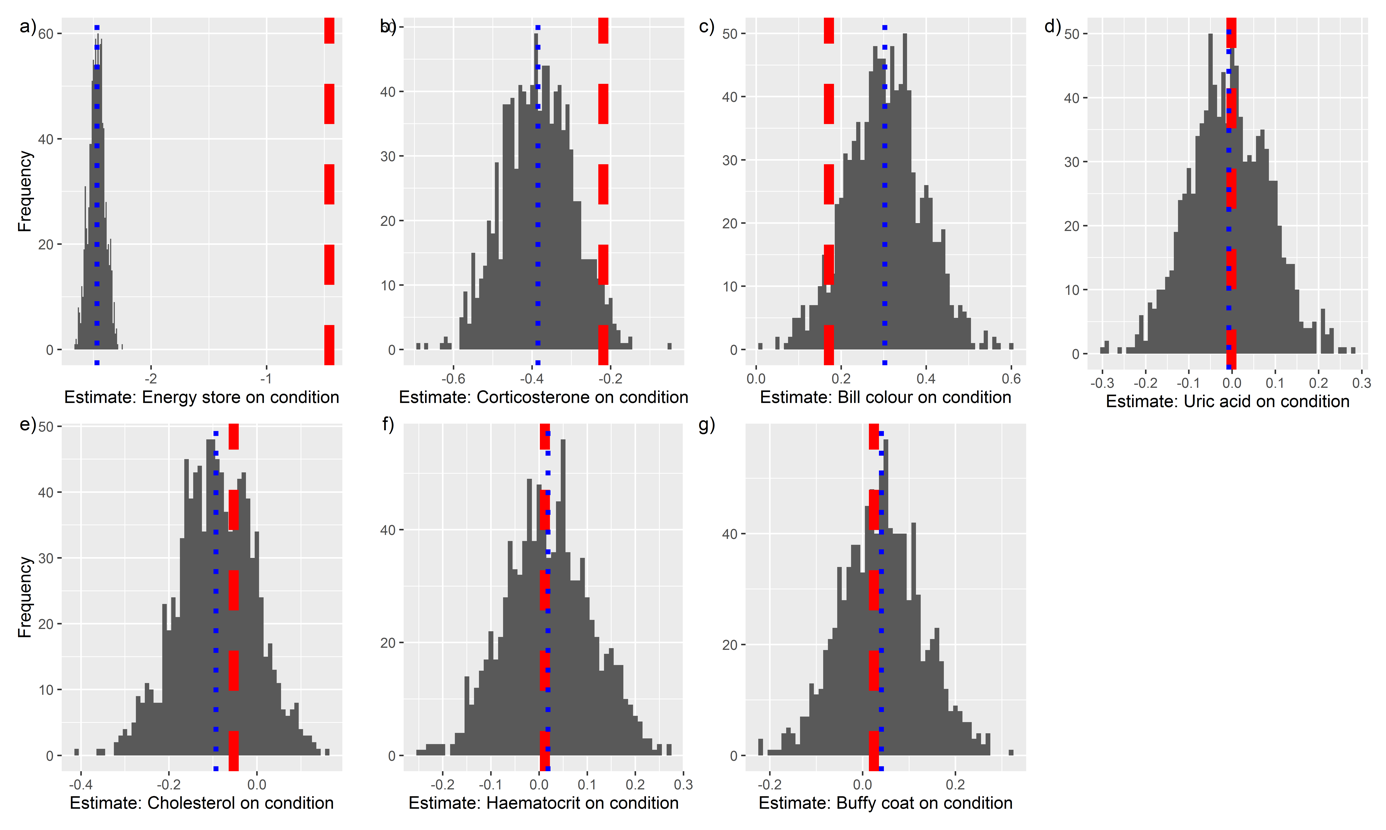


Figure 15: Histogram of the effect of each condition variable on condition (estimate) of the simulated datasets for the conventional approach. Red dashed line indicates the ‘true’ mean, whereas the blue dotted line is the mean of the simulated data.

*
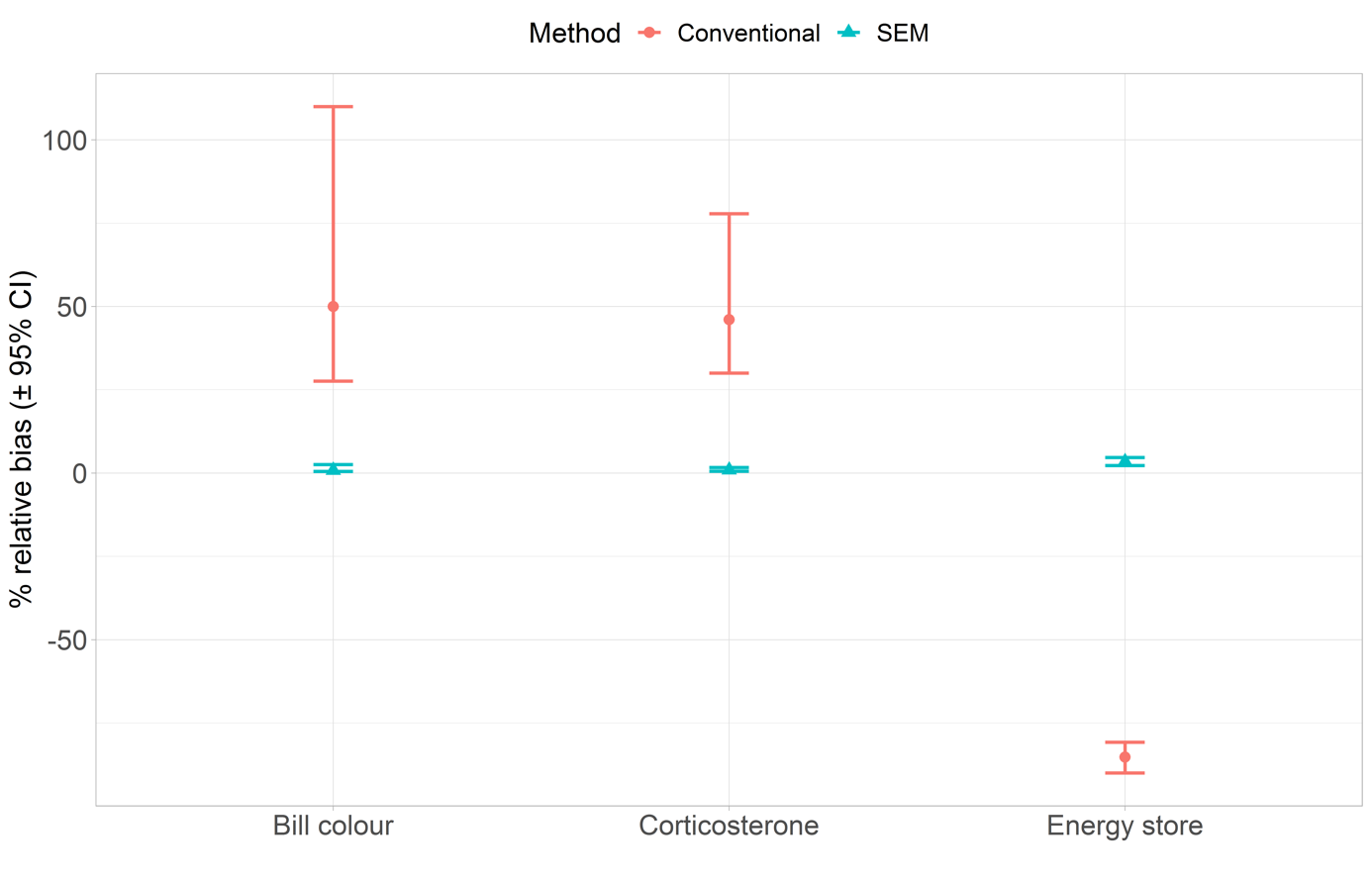
*

*Figure S16: Relative bias (simulated estimate-true estimate divided by simulated estimate multiplied by 100) for three condition variables with strong effect on the condition index. SEM approach appears to be more unbiased than the conventional approach.*

*
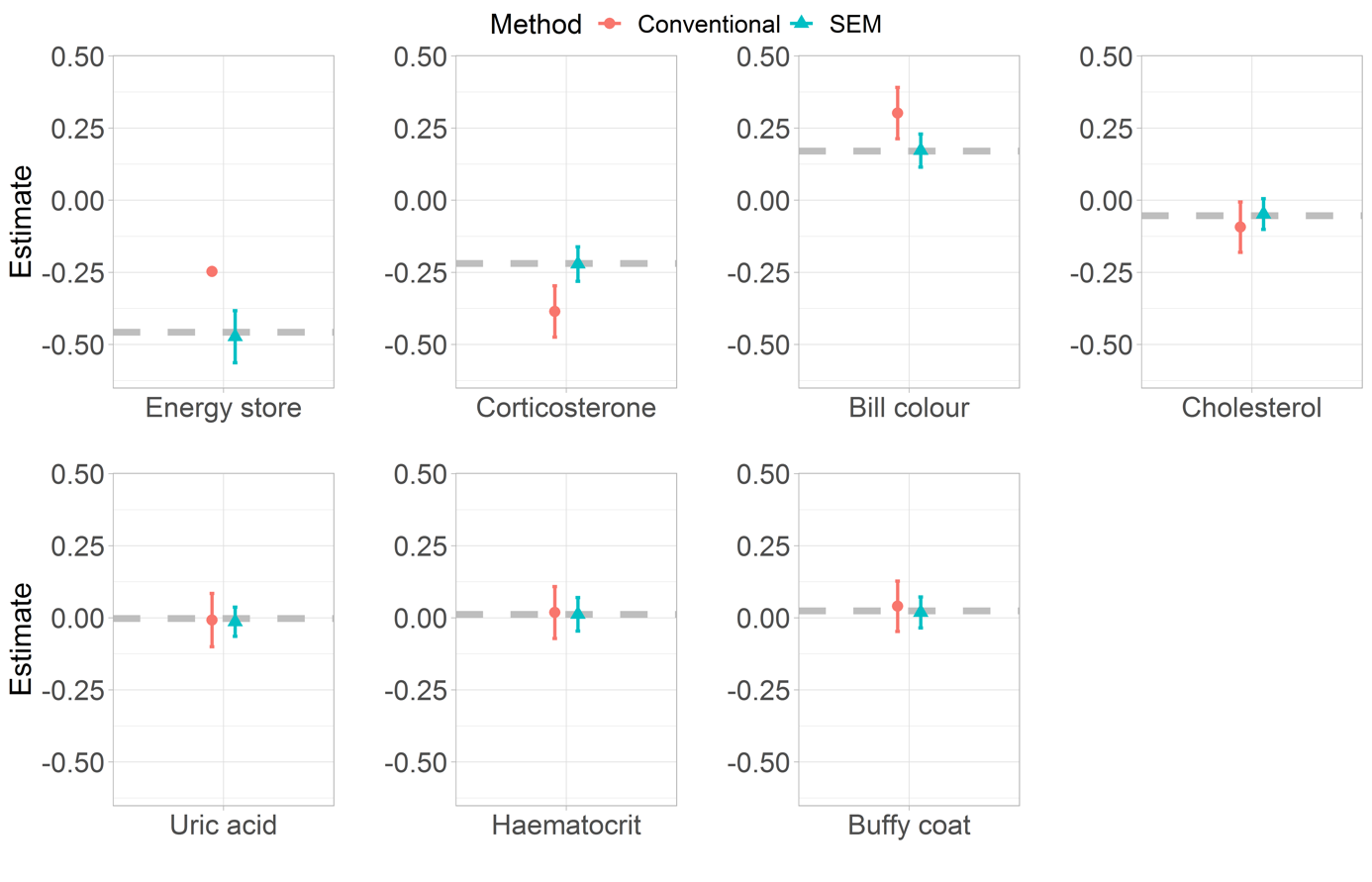
*

Figure S17: Results from the simulation comparing biases in the estimation of the slopes of the condition variables using the SEM and the conventional approach. Simulation (with 1000 datasets of 1000 observations) was based on the covariance patterns found in the case study population (Fig. 6a) using the ‘simulateData’ function from lavaan (Rosseel, 2012). Dots and triangles indicate the mean simulated estimate for the conventional method and SEM, respectively, with the empirical standard error. Dashed grey horizontal line indicates the ‘true’ estimates. Note that standard error of the variable ‘energy store’ is really small and error bars are therefore hardly visible.

*
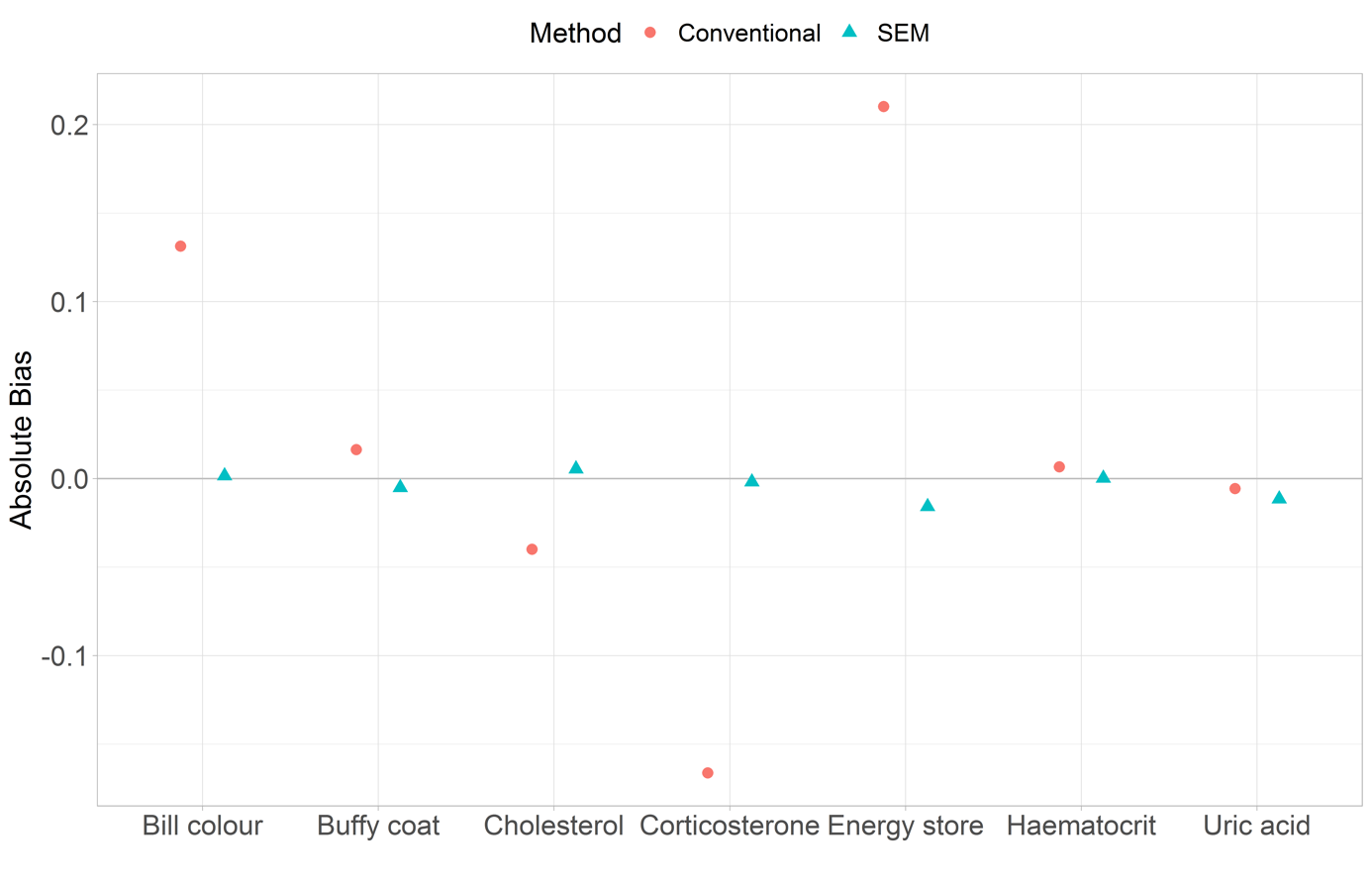
*

*Figure S18: Absolute bias for each condition variable for each approach. SEM being indicated by triangles and conventional approach by dots.*

*
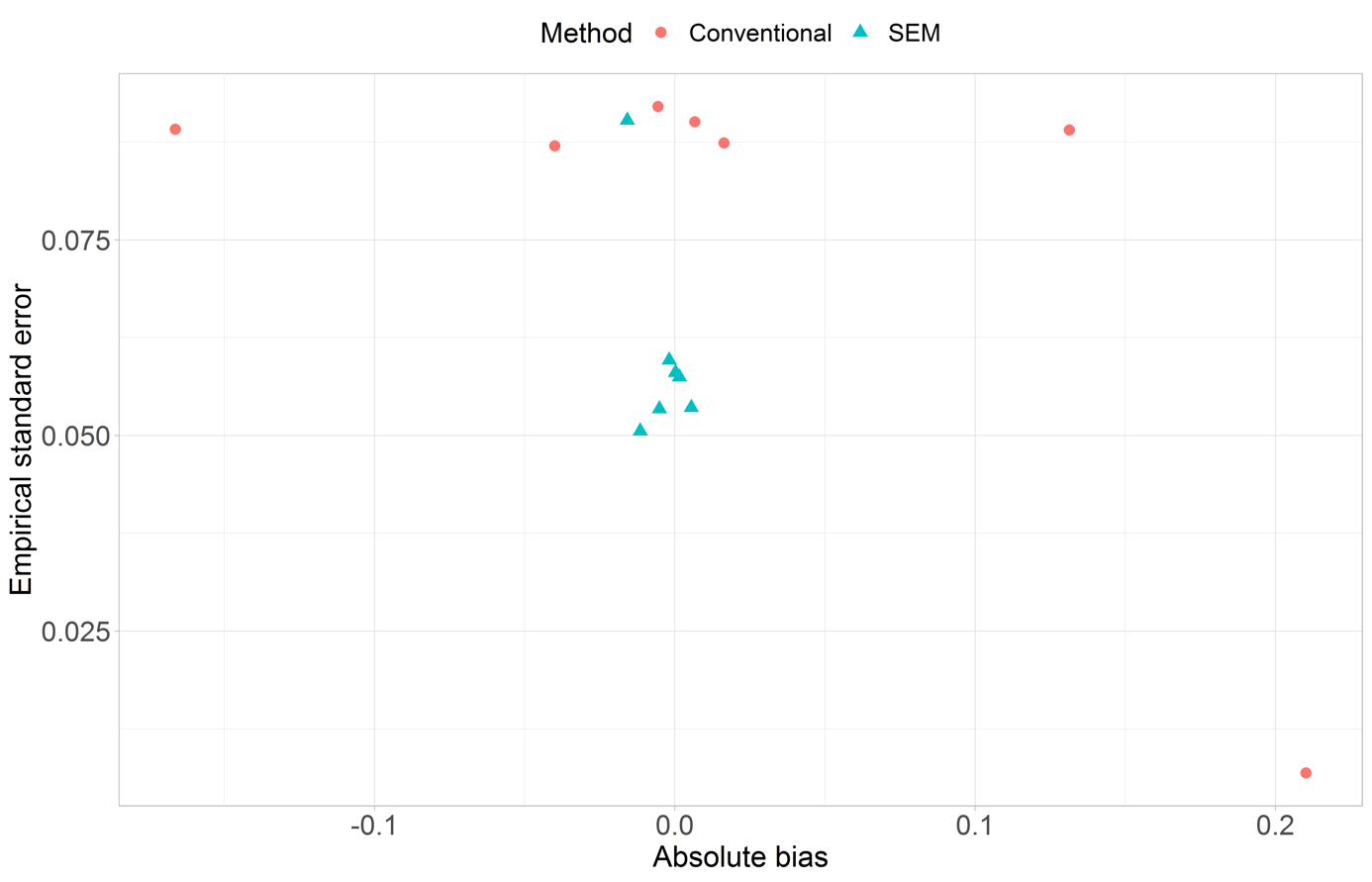
*

*Figure S19: Bias plotted against the empirical standard error for each condition estimate per approach. The SEM approach is clearly more unbiased compared to the conventional approach. In six of the seven variables the estimates are more precise than the estimates from the conventional approach. Only the variable energy store was estimated more precise with the conventional approach, however, this estimate was highly biased.*

*
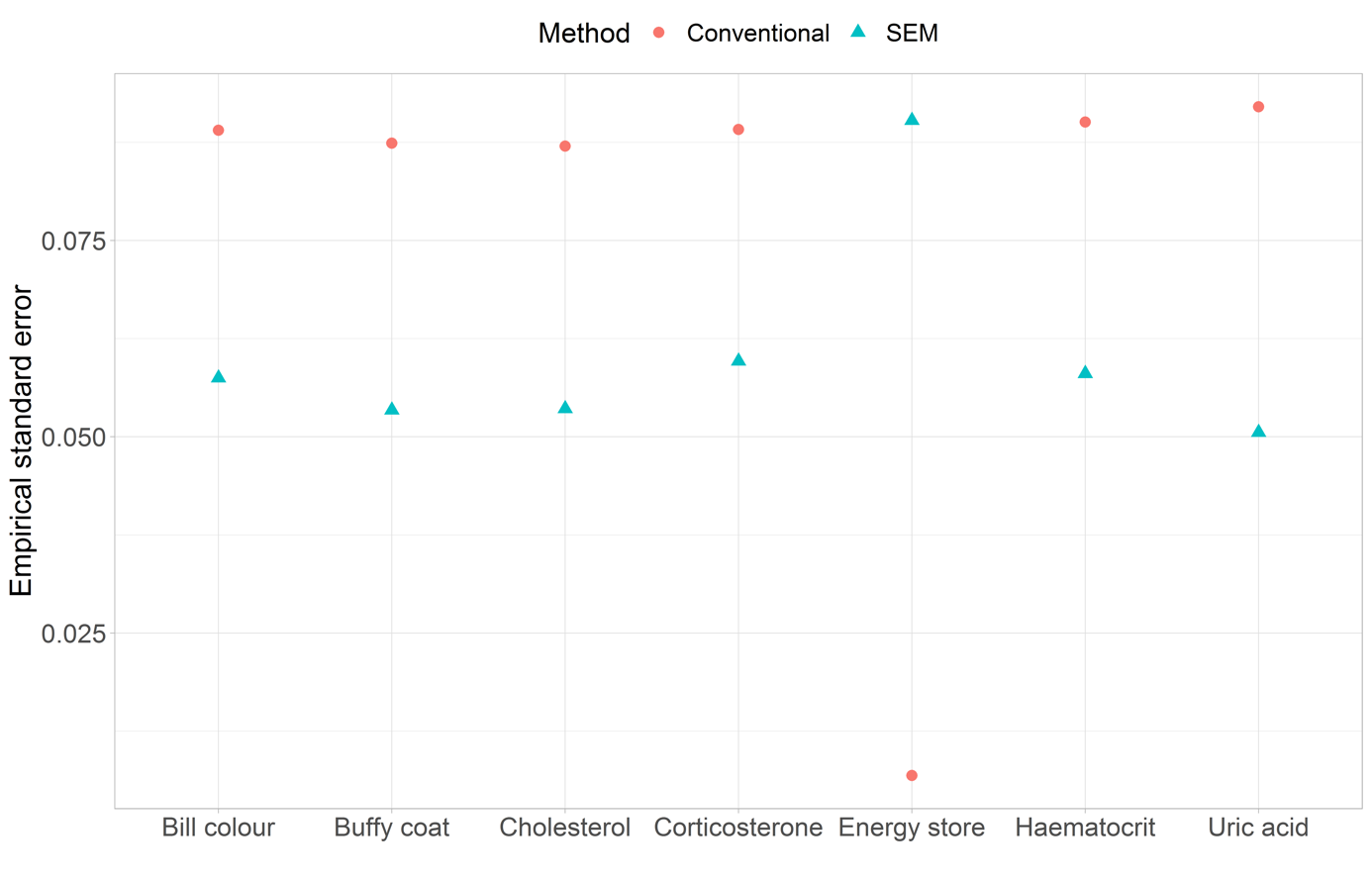
*

*Figure S20: Empirical standard error (precision) per variable and statistical approach.*


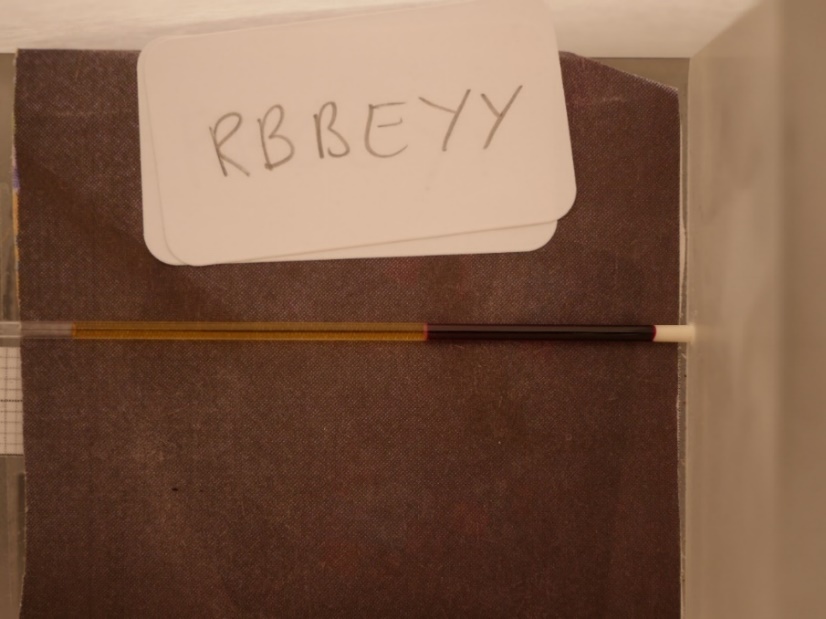


Figure S21: Holder for the capillaries for the blood samples to measure the haematocrit and buffy coat.


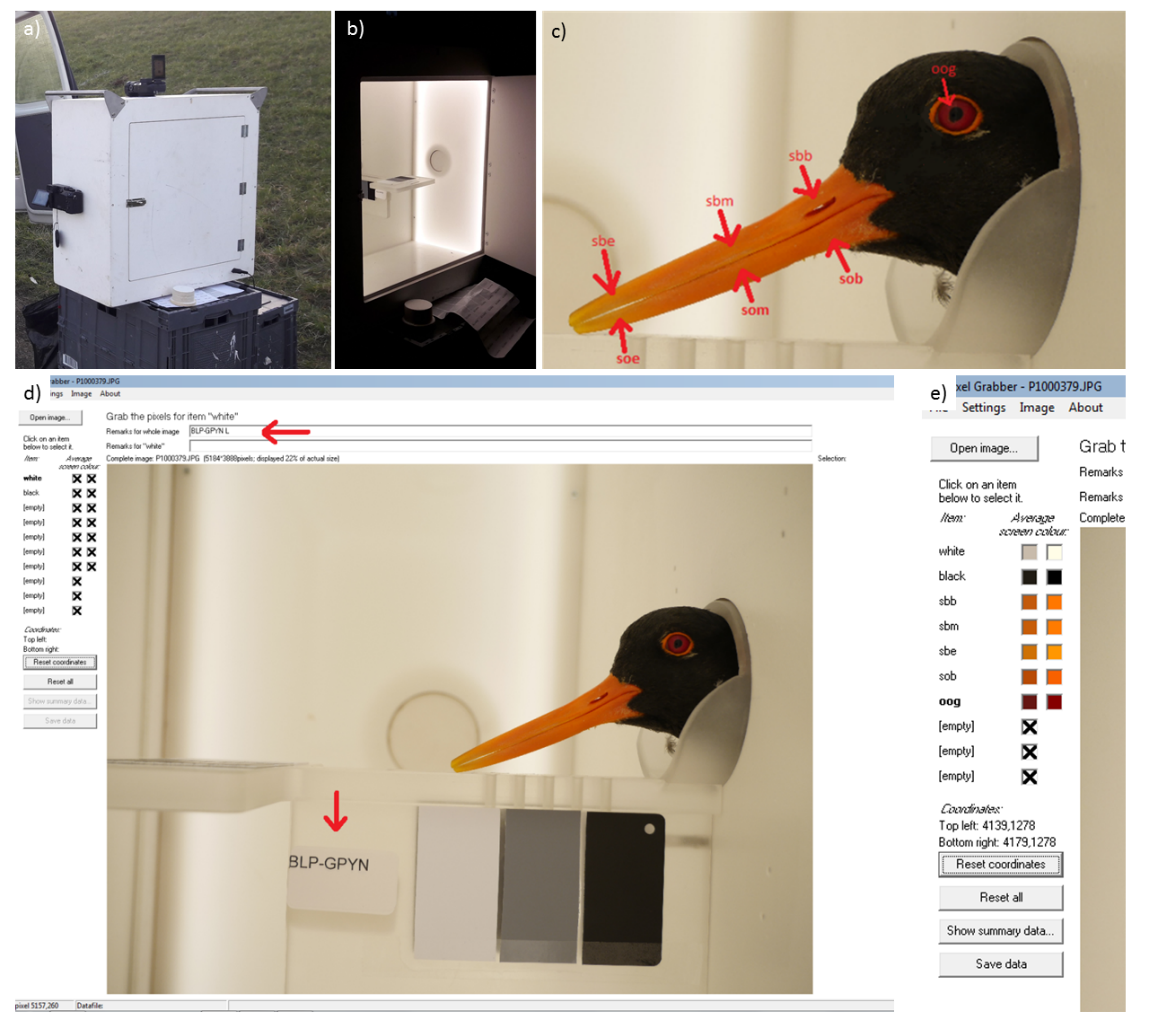


Figure S22: a) showing the box where camera was placed on the side as well as from above to take a picture of the oystercatcher head. b) shows the box from inside with illumination, which ensured standardized light conditions for each picture. c) illustrates the different measuring points of each individual with the program pixelgrabber (d) where each individual is labelled on the picture next to the three reference cards for balancing the colour. e) shows an example of balanced colours on the right side corrected for the “real” white, on the left side the “original” colour.


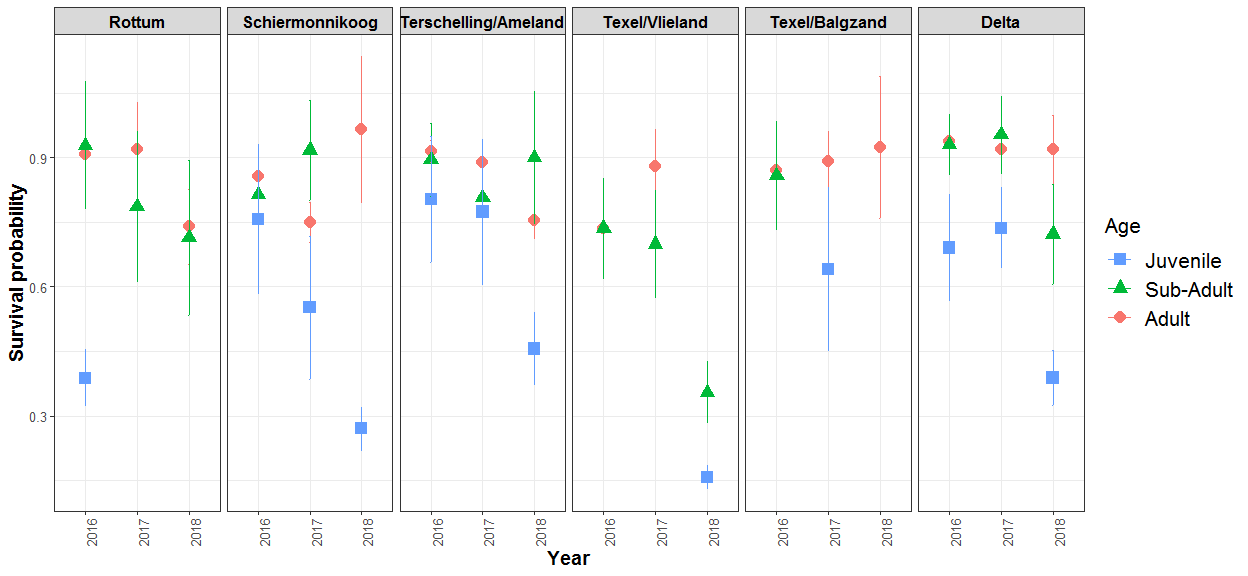


Figure S23: Seasonal survival probability for the different years, catching areas and age classes. Dots with bar indicate the estimate with the standard error. Year 2016 refers to winter 2016/2017


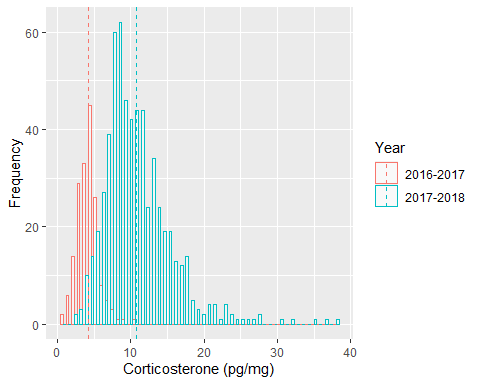


Figure S24: Corticosterone (pg/mg) measurements for the two different years of extraction (2016-2017 & 2017-2018)


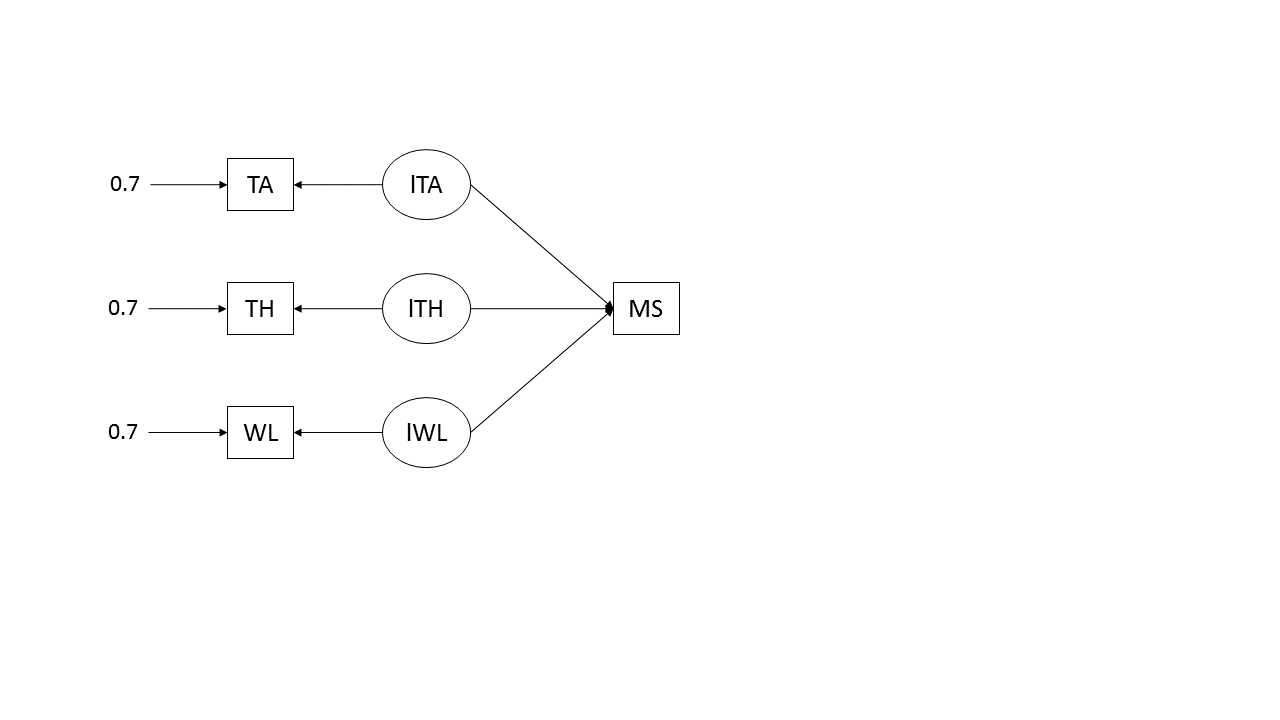


Figure S25: Schematic representation of the model by accounting for measurement errors (0.7 on each variable) by including latent variables (circles). The measured variables (squares) are tarsus length (TA), head length (TH) and wing length (WL) which are regressed on mass (MS) through the latent variables.


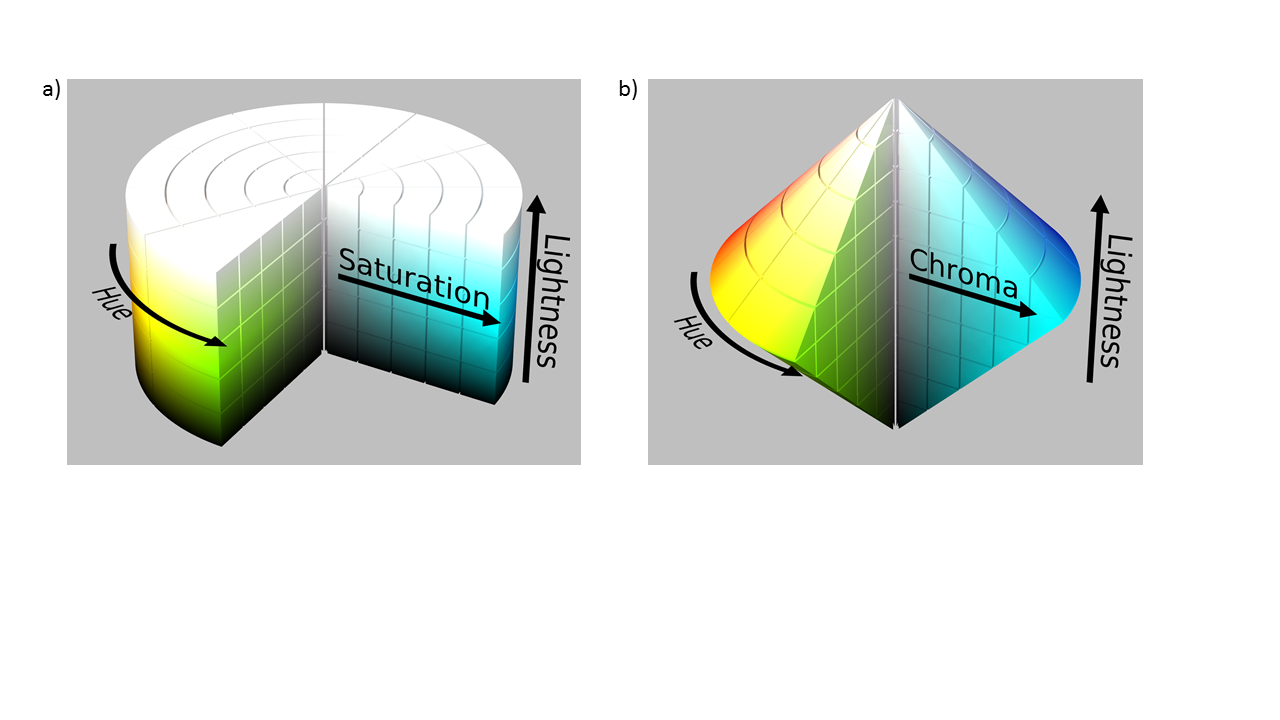


*Figure S26: Relationship of hue, lightness and a) saturation or b) chroma in a 3-dimensional setting. Picture credits: a) SharkD Talk - HSL_color_solid_cylinder.png,* [*https://commons.wikimedia.org/w/index.php?curid=9801661*](https://commons.wikimedia.org/w/index.php?curid=9801661)*; b) SharkD Talk Hcl-hcv_models.svgHSL_color_solid_dblcone.png,* [*https://commons.wikimedia.org/w/index.php?curid=9802536*](https://commons.wikimedia.org/w/index.php?curid=9802536)

# Tables

Table S1: Catching events and their characteristics.

| Date | Location | Longitude | Latitude | Winter | Number of birds | | |
| --- | --- | --- | --- | --- | --- | --- | --- |
|  |  |  |  |  | Juveniles | Sub-Adults | Adults |
| 2-12-2016 | Vlieland, Vliehors | 4.95 | 53.24 | 2016-2017 | 0 | 5 | 1 |
| 3-12-2016 | Vlieland, Vliehors | 4.95 | 53.24 | 2016-2017 | 4 | 0 | 1 |
| 4-12-2016 | Vlieland, Vliehors | 4.95 | 53.24 | 2016-2017 | 2 | 0 | 0 |
| 5-12-2016 | Vlieland, Vliehors | 4.95 | 53.24 | 2016-2017 | 0 | 0 | 1 |
| 18-12-2016 | Vlieland, Vliehors | 4.97 | 53.25 | 2016-2017 | 1 | 2 | 3 |
| 18-12-2016 | Vlieland, Vliehors | 4.97 | 53.25 | 2016-2017 | 6 | 2 | 5 |
| 18-12-2016 | Vlieland, Vliehors | 4.97 | 53.25 | 2016-2017 | 1 | 0 | 0 |
| 19-12-2016 | Vlieland, Vliehors | 4.97 | 53.25 | 2016-2017 | 1 | 0 | 0 |
| 16-1-2017 | Vlieland, Vliehors | 4.95 | 53.24 | 2016-2017 | 0 | 1 | 1 |
| 17-1-2017 | Vlieland, Vliehors | 4.95 | 53.24 | 2016-2017 | 0 | 30 | 45 |
| 18-1-2017 | Vlieland, Vliehors | 4.95 | 53.24 | 2016-2017 | 0 | 5 | 5 |
| 25-1-2017 | Schiermonnikoog 2e slenk | 6.23 | 53.48 | 2016-2017 | 0 | 14 | 34 |
| 20-2-2017 | Balgzand Normerven | 4.94 | 52.91 | 2016-2017 | 0 | 30 | 37 |
| 24-2-2017 | Eemshaven | 6.78 | 53.46 | 2016-2017 | 0 | 4 | 14 |
| 28-2-2017 | Molenkolk, Texel | 4.80 | 53.02 | 2016-2017 | 0 | 8 | 49 |
| 15-12-2017 | Vlieland, Vliehors | 4.96 | 53.25 | 2017-2018 | 1 | 11 | 24 |
| 18-12-2017 | Vlieland, Vliehors | 4.96 | 53.25 | 2017-2018 | 12 | 12 | 16 |
| 19-12-2017 | Vlieland, Vliehors | 4.96 | 53.25 | 2017-2018 | 0 | 5 | 11 |
| 19-12-2017 | Vlieland, Vliehors | 4.92 | 53.23 | 2017-2018 | 0 | 8 | 32 |
| 6-2-2018 | Oosterend, Terschelling | 5.39 | 53.40 | 2017-2018 | 0 | 0 | 3 |
| 26-1-2018 | Balgzand, ’t Kuitje | 4.79 | 52.93 | 2017-2018 | 0 | 6 | 69 |
| 19-1-2018 | Prunjepolder, Oosterschelde | 3.85 | 51.69 | 2017-2018 | 0 | 21 | 108 |
| 21-1-2018 | Ameland, Vogelpolle | 5.67 | 53.42 | 2017-2018 | 0 | 25 | 77 |
| 16-2-2018 | Striep, Terschelling | 5.30 | 53.38 | 2017-2018 | 0 | 1 | 17 |
| 17-2-2018 | Sehael, Terschelling | 5.32 | 53.38 | 2017-2018 | 0 | 5 | 45 |
| 30-1-2018 | Schor Wilhelminapolder, Oosterschelde | 3.90 | 51.54 | 2017-2018 | 0 | 16 | 106 |
| 19-2-2018 | Schiermonnikoog, 2e Slenk | 6.23 | 53.48 | 2017-2018 | 0 | 36 | 48 |
| 20-2-2018 | Schiermonnikoog, 2e Slenk | 6.23 | 53.48 | 2017-2018 | 0 | 12 | 22 |

Table S2: List of variables and their characteristics included in the analyses.

| **Variable name** | **Unit** | **Type** | **Mean** | **SD** | **Min** | **Max** | **Nr. Obs. **** | **Transformation ***** |
| --- | --- | --- | --- | --- | --- | --- | --- | --- |
| Body weight | gr | Continuous | 564 | 50 | 439 | 774 | 772 | - |
| Tarsus length | mm | Continuous | 97 | 3 | 87 | 108 | 774 | - |
| Head/bill length | mm | Continuous | 120 | 6 | 105 | 142 | 773 | - |
| Wing length | mm | Continuous | 265 | 6 | 245 | 286 | 767 | - |
| Cholesterol | mmol/L | Continuous | 13.2 | 2.6 | 6.9 | 26.2 | 408 | - |
| Bill chroma | - | Continuous | 85 | 2 | 83 | 93 | 589 | - |
| Bill luminance | - | Continuous | 69 | 3 | 61 | 79 | 589 | - |
| Bill hue | - | Continuous | 63 | 5 | 47 | 79 | 600 | - |
| Haematocrit | - | Proportion | 0.45 | 0.03 | 0.38 | 0.54 | 549 | Logit |
| Buffy coat | - | Proportion | 0.006 | 0.002 | 0.001 | 0.018 | 551 | Logit |
| Uric acid | mmol/L | Continuous | 1.18 | 0.73 | 0.23 | 3.99 | 386 | - |
| Corticosterone (standardized*) | pg/mg | Continuous | 0.0 | 1.0 | -2.6 | 6.0 | 607 | - |
| Handling time | - | Proportion | 0.14 | 0.06 | 0.02 | 0.30 | 774 | - |
| Bill tip height | mm | Continuous | 4.2 | 0.9 | 1.5 | 6.4 | 768 | - |
| Sex | - | Factor | Used as continuous variable in the model.  Females (F, n=311) coded as 1 (reference group),  males (M, n=463) coded as 2. | | | | 744 | - |
| Density | number/km^2^ | Continuous | 114 | 68 | 14 | 255 | 774 | - |
| Survival | - | Proportion | 0.87 | 0.06 | 0.74 | 0.92 | 774 | Logit |

* Standardized among years. For more information see Fig. S24.

** Note that not all variables were collected during each capture and therefore sample sizes varied among analyses.

*** Transformation before analysis.

*Table S3: Pearson’s correlation between the three colour parameters per age class.*

| **Correlation between** | **Adults** | **Sub-Adults** |
| --- | --- | --- |
| Hue & Luminance | 0.97 | -0.11 |
| Chroma & Hue | -0.83 | -0.41 |
| Luminance & Chroma | -0.70 | 0.94 |

Table S4: Loadings from PC1 and PC2 of the energy-store-PCA. PCA analyses were conducted with the R-package FactoMineR (Le et al., 2008).

|  | PC1 | PC2 |
| --- | --- | --- |
| Residuals | 0.98 | 0.18 |
| Ratio | 0.98 | -0.18 |

Table S5: Loadings from the PC1, PC2 and PC3 of the adult bill colour. PCA analyses were conducted with the R-package FactoMineR (Le et al., 2008).

|  | PC1 | PC2 | PC3 |
| --- | --- | --- | --- |
| Hue | 0.99 | 0.10 | 0.08 |
| Luminance | 0.95 | 0.31 | -0.06 |
| Chroma | -0.90 | 0.44 | 0.02 |

Table S6: Loadings from the PC1, PC2 and PC3 of the sub-adult bill colour. PCA analyses were conducted with the R-package FactoMineR (Le et al., 2008).

|  | PC1 | PC2 | PC3 |
| --- | --- | --- | --- |
| Hue | -0.49 | 0.87 | 0.02 |
| Luminance | 0.92 | 0.39 | -0.05 |
| Chroma | 1.00 | 0.07 | 0.06 |

Table S7: Model fit indices of all models. Statistical significance of the chi-square statistic (using alpha = 0.05) indicates that the fit of the model has to be rejected. With increasing sample sizes, chance of detecting a smaller difference between the hypothesized value and the true value is greater and therefore may lead to rejection of the model (Grace & Keeley, 2006; Jak & Jorgensen, 2017; Shipley, 2016). Since we have relatively high sample sizes (>300), we also document alternative fit indices: the root mean square error of approximation (RMSEA; Kline, 2011), standardized root mean square residual (SRMR; Hu & Bentler, 1999) and the comparative fit index (CFI; Bentler, 1990).

|  | Chi-square | p-value | DF | CFI | SRMR | RMSEA | Lower RMSEA | Upper RMSEA |
| --- | --- | --- | --- | --- | --- | --- | --- | --- |
| Energy store model (Fig. 2a, Code S1) | 0 | NA | 0 | 1 | 0 | 0 | 0 | 0 |
| Bill colour model (Fig. 2b, Code S2) | 1141.24 | <0.001 | 2 | 0.68 | 0.06 | 1.22 | 1.16 | 1.28 |
| Composite model (Fig. 4, Code S3) | 77.83 | <0.001 | 9 | 0.78 | 0.05 | 0.13 | 0.10 | 0.15 |
| Composite model with squared mass (Fig. 4, Code S4) | 77.19 | <0.001 | 12 | 0.65 | 0.07 | 0.11 | 0.09 | 0.13 |
| Latent+composite model (Fig. 5, Code S5) | 363.72 | <0.001 | 68 | 0.92 | 0.08 | 0.10 | 0.09 | 0.11 |
| Latent+composite model with direct and indirect effect of body size (Code S6) | 276.97 | <0.001 | 51 | 0.94 | 0.08 | 0.10 | 0.09 | 0.11 |
| Latent+composite model with only indirect effect of body size (Code S7) | 277.63 | <0.001 | 52 | 0.94 | 0.08 | 0.10 | 0.09 | 0.11 |
| Model used for model performance (Fig. 6; Code S8) | 246.14 | <0.001 | 60 | 0.95 | 0.07 | 0.09 | 0.07 | 0.10 |
| Acceptable threshold levels | Low chi-square relative to degrees of freedom with an insignificant p value (p > 0.05) | | | >0.90 | <=0.08 | <=0.07 | | |
| Literature | Grace, 2006 | | | Hu & Bentler, 1999; Lefcheck, 2019 | Hu & Bentler, 1999 | Steiger, 2007 | | |

*Table S8: Comparison of the two full models (Fig. S12). Model 1 indicating the model without a direct effect of body size, Model 2 indicating the model with a direct effect of body size on survival.*

| Model | DF | AIC | BIC | Chisq | Chisq diff | DF diff | Pr(>Chisq) |
| --- | --- | --- | --- | --- | --- | --- | --- |
| Model 1 (Fig. S13b; Code S6) | 51 | -5278.3 | -5083.6 | 276.97 |  |  |  |
| Model 2 (Fig. S13a, Code S7) | 52 | -5279.7 | -5089.0 | 277.64 | 0.66635 | 1 | 0.4143 |

Table S9: Steps that needs to be taken to analyse the model (Fig. 5) with conventional methods (MR=multiple regression, PCA=principal component analysis). Note that here we show 6 steps instead of 4 steps (as described in Fig. 6). Step 2 and 3 in this table relate to step 2 in Fig. 6 and step 5 and 6 in this table relate to step 4 in Fig. 6. The reason for this is that technically, extracting the residuals and running the PCA are two steps, but for easier visualization we showed it as one step (step 2) in Fig. 6. Explanatory variables (from step 1) were standardized (mean=0, sd=1) before analysis. Abbreviations: MS=mass, TL=tarsus length, WL=wing length, HL=head length, HT=handling time, BH=bill tip height, Cho=cholesterol, U=uric acid, B=buffy coat, Hct=haematocrit, Cort=corticosterone, Age=Age, Sex=Sex.

| **Step 1** | **Step 2** | **Step 3** | **Step 4** | **Step 5** | **Step 6** |
| --- | --- | --- | --- | --- | --- |
| *MR: condition variables as response variable with individual characteristics and confounding variables as independent variables* | *Extract residuals* | *PCA: run PCA and extract PC1* | *MR: regress confounding variables and individual characteristics on PC1* | *Extract residuals from PC1* | *MR: survival as response variable and residuals of all condition variables* |
| MS~TL+WL+HL | Resid(MS) | PCA with Resid(MS) and Ratio | PC1 Energy store~Age+Sex+BH+HT | Resid(PC1 Energy reserve) | Survival ~ resid(PC1 Energy store)+ resid(Hue)+resid(Cho)+resid(U)+resid(BC)+resid(Hct)+resid(Cort) |
| Ratio |  |  |  |  |  |
| Cho~Sex+HT+BH+Age | Resid(Cho) |  |  |  |  |
| U~Sex+HT+BH+Age | Resid(U) |  |  |  |  |
| B~Sex+HT+BH+Age | Resid(B) |  |  |  |  |
| Hct~Sex+HT+BH+Age | Resid(Hct) |  |  |  |  |
| Cort~Sex+BH+Age | Resid(Cort) |  |  |  |  |
| Hue~ Sex+BH+Age | Resid(Hue) |  |  |  |  |

Table S10: Pearson’s correlation of different measuring points of the bill of an individual. Values in parentheses indicate the lower and upper 95% confidence interval. See for abbreviations and locations sbm, som, sbb, sbe Fig. S22.

|  |  | **Hue** | **Chroma** | **Luminance** | **n** |
| --- | --- | --- | --- | --- | --- |
| **Bill** | *Right vs. left side (sbm)* | 0.89 (0.86,0.92) | 0.91 (0.88, 0.94) | 0.89 (0.86,0.92) | 160 |
|  | *Sbm (Fig. S22) vs. som on left side* | 0.88 (0.84, 0.91) | 0.86 (0.81, 0.89) | 0.79 (0.72, 0.84) | 167 |
|  | *Sbm vs. sbb on left side* | 0.76 (0.67, 0.84) | 0.65 (0.52, 0.75) | 0.48 (0.30, 0.62) | 94 |
|  | *Sbm vs. sbe on left side* | 0.35 (0.21, 0.48) | 0.84 (0.78, 0.88) | 0.82 (0.76, 0.86) | 166 |

# Codes

The following codes and explanations aim to illustrate and explain the coding while reading the paper. Reproducing the codes with the data is possible with the available Rmark down file (Analyses.Rmd) and data file (data.csv) on Github and can be accessed via <https://github.com/MagaliFr/QuantifyingBodyConditionWithSEM>.

Abbreviations for the variables in all R-codes:

ratio: ratio of mass divided by tarsus length
MS: mass
TA: tarsus length
TH: head length
WL: wing length
ES: Energy store
BCol: bill colour
LS: bill luminance
CS: bill chroma
HS: bill hue
Comp: composite variable=body condition
LogitSurvWS: Survival
Density: Oystercatcher density (number/km^2^)
SexN: Sex (numeric variable)
nBH: standardized bill tip height (mean=0, sd=1)
nHT: standardized handling time (mean=0, sd=1)
CortStd: Corticosterone
U_perVolBlood: Uric acid
Cho_perVolBlood: Cholesterol
LogitH: Haematocrit
LogitB: Buffy coat

AgeN: Age (1=sub-adults, 2=adults)

Residuals: Residuals of mass (Mass ~ wing length + tarsus length + head length)

All Statistical analyses in this paper were done in R (version 3.5.1) (R Core Team, 2019).

Libraries used for the following coding:

- lavaan version 0.6-5

- caret version 6.0-80

Operators used in lavaan:

=~ latent variable

<~ composite variable

~~ residual covariance

~ regression

All analysis were based on the co-variances between variables.

Code S1: The code shows the code for Fig. 2a. The error variance of ratio turns out to be negative (but really close to 0) so that it is acceptable to fix the error variance of the variable ratio to 0. The R-package used for the analysis is lavaan (Rosseel, 2012).

library(lavaan)

lvmod.2 <-'
ES =~ ratio + Residuals #=~ annotation for defining latent variable
Residuals~~0*Residuals
'

lvmod.1.fit<-cfa(lvmod.1,data=f) #fits the model
summary(lvmod.1.fit, rsq=T, standardized=T) #gives model output

## lavaan 0.6-5 ended normally after 20 iterations
##
## Estimator ML
## Optimization method NLMINB
## Number of free parameters 3
##
## Number of observations 1021
##
## Model Test User Model:
##
## Test statistic 0.000
## Degrees of freedom 0
##
## Parameter Estimates:
##
## Information Expected
## Information saturated (h1) model Structured
## Standard errors Standard
##
## Latent Variables:
## Estimate Std.Err z-value P(>|z|) Std.lv Std.all
## ES =~
## ratio 1.000 0.459 0.932
## Residuals 0.097 0.001 82.169 0.000 0.045 1.000
##
## Variances:
## Estimate Std.Err z-value P(>|z|) Std.lv Std.all
## .Residuals 0.000 0.000 0.000
## .ratio 0.032 0.001 22.594 0.000 0.032 0.131
## ES 0.211 0.011 19.798 0.000 1.000 1.000
##
## R-Square:
## Estimate
## Residuals 1.000
## ratio 0.869

fitMeasures(lvmod.1.fit, c("chisq", "pvalue", "df","cfi", "srmr", "rmsea", "rmsea.ci.lower", "rmsea.ci.upper")) #gives model fit indices

## chisq pvalue df cfi srmr
## 0 NA 0 1 0
## rmsea rmsea.ci.lower rmsea.ci.upper
## 0 0 0

Code S2: SEM for bill colour analysis (Fig. 2a). The error variance of the variable LS (luminance) turns out to be slightly negative (close to 0) so that it is acceptable to fix the error variance of the variable LS to 0. We conducted a multi-group analysis of age class (group=”Age”). Results show the outcome for each age class. The R-package used for the analysis is lavaan (Rosseel, 2012).

f$AgeN<-ifelse(f$Age=="Adult",2,1)

library(lavaan)
lvmod.2 <-'
BCol=~LS+HS+CS #define latent variable BCol=Bill colour
LS~~0*LS #fix error variance of luminance to 0
'
lvmod.2.fit<-cfa(lvmod.2,data=f, group="Age") #fit model
summary(lvmod.2.fit, rsq=T, standardized=T) #model output

## lavaan 0.6-5 ended normally after 66 iterations
##
## Estimator ML
## Optimization method NLMINB
## Number of free parameters 16
##
## Number of observations per group:
## Adult 599
## Sub-Adult 163
##
## Model Test User Model:
##
## Test statistic 1141.244
## Degrees of freedom 2
## P-value (Chi-square) 0.000
## Test statistic for each group:
## Adult 787.835
## Sub-Adult 353.409
##
## Parameter Estimates:
##
## Information Expected
## Information saturated (h1) model Structured
## Standard errors Standard
##
##
## Group 1 [Adult]:
##
## Latent Variables:
## Estimate Std.Err z-value P(>|z|) Std.lv Std.all
## BCol =~
## LS 1.000 3.508 1.000
## HS 1.456 0.015 94.952 0.000 5.107 0.968
## CS -0.387 0.015 -25.357 0.000 -1.357 -0.720
##
## Intercepts:
## Estimate Std.Err z-value P(>|z|) Std.lv Std.all
## .LS 68.892 0.143 480.618 0.000 68.892 19.638
## .HS 63.320 0.215 293.846 0.000 63.320 12.006
## .CS 84.494 0.077 1096.179 0.000 84.494 44.789
## BCol 0.000 0.000 0.000
##
## Variances:
## Estimate Std.Err z-value P(>|z|) Std.lv Std.all
## .LS 0.000 0.000 0.000
## .HS 1.733 0.100 17.306 0.000 1.733 0.062
## .CS 1.716 0.099 17.306 0.000 1.716 0.482
## BCol 12.307 0.711 17.306 0.000 1.000 1.000
##
## R-Square:
## Estimate
## LS 1.000
## HS 0.938
## CS 0.518
##
##
## Group 2 [Sub-Adult]:
##
## Latent Variables:
## Estimate Std.Err z-value P(>|z|) Std.lv Std.all
## BCol =~
## LS 1.000 8.534 1.000
## HS -0.055 0.035 -1.550 0.121 -0.468 -0.121
## CS 1.033 0.029 35.923 0.000 8.820 0.942
##
## Intercepts:
## Estimate Std.Err z-value P(>|z|) Std.lv Std.all
## .LS 53.267 0.668 79.687 0.000 53.267 6.242
## .HS 65.298 0.304 214.763 0.000 65.298 16.822
## .CS 67.158 0.733 91.599 0.000 67.158 7.175
## BCol 0.000 0.000 0.000
##
## Variances:
## Estimate Std.Err z-value P(>|z|) Std.lv Std.all
## .LS 0.000 0.000 0.000
## .HS 14.850 1.645 9.028 0.000 14.850 0.985
## .CS 9.826 1.088 9.028 0.000 9.826 0.112
## BCol 72.835 8.068 9.028 0.000 1.000 1.000
##
## R-Square:
## Estimate
## LS 1.000
## HS 0.015
## CS 0.888

fitMeasures(lvmod.2.fit, c("chisq", "pvalue", "df","cfi", "srmr", "rmsea", "rmsea.ci.lower", "rmsea.ci.upper"))# model fit indices

## chisq pvalue df cfi srmr
## 1141.244 0.000 2.000 0.683 0.061
## rmsea rmsea.ci.lower rmsea.ci.upper
## 1.223 1.164 1.283

*Code S3: Code for the composite variable model (Fig. 5). Note that in this example the model fit index (CFI) indicates poor fit. However, remember that this serves as a simple illustration for running a composite variable in SEM and is not yet biologically correct e.g. missing confounding variables. The first variable (determining the composite variable) should always be multiplied by 1 to set the scale (for other variables). We multiplied the first variable “ratio” by -1 because it has a negative effect on the condition. If we multiply it by 1 we get a negative effect of condition on survival which makes interpretation more difficult. The absolute number of the coefficients stay the same (when multiplying by 1 or -1). The R-package used for the analysis is lavaan (Rosseel, 2012).*

library(lavaan)
# Variable scaling
d1$MSK<-d1$MS/1000
d1$Dens<-d1$Density/1000
d1$TA100<-d1$TA/100
d1$TH100<-d1$TH/100
d1$WL100<-d1$WL/100
d1$Cho10<-d1$Cho_perVolBlood/10

Cmod.1 <- '

# define the composite variable
Comp <~ -1*lMS+CortStd+U_perVolBlood+Cho10+LogitH+LogitB
lMS =~ MSK # create latent variable of mass
lMS~WL100+TA100+TH100 # regress structural sizes on latent mass
LogitSurvWS~Comp # regress the composite on survival
Comp~Dens # regress the density on the composite
'
Cmod.1.fit<-sem(Cmod.1,data=d1) # fit the model
summary(Cmod.1.fit, rsq=T, standardized=T)# get the output of the sem

## lavaan 0.6-5 ended normally after 68 iterations
##
## Estimator ML
## Optimization method NLMINB
## Number of free parameters 12
##
## Number of observations 447
##
## Model Test User Model:
##
## Test statistic 77.832
## Degrees of freedom 9
## P-value (Chi-square) 0.000
##
## Parameter Estimates:
##
## Information Expected
## Information saturated (h1) model Structured
## Standard errors Standard
##
## Latent Variables:
## Estimate Std.Err z-value P(>|z|) Std.lv Std.all
## lMS =~
## MSK 1.000 0.050 1.000
##
## Composites:
## Estimate Std.Err z-value P(>|z|) Std.lv Std.all
## Comp <~
## lMS -1.000 -0.744 -0.744
## CortStd -0.028 0.009 -3.160 0.002 -0.412 -0.373
## U_perVolBlood -0.017 0.011 -1.561 0.119 -0.256 -0.185
## Cho10 -0.028 0.033 -0.833 0.405 -0.412 -0.110
## LogitH 0.044 0.077 0.568 0.570 0.654 0.076
## LogitB 0.028 0.027 1.029 0.304 0.420 0.112
##
## Regressions:
## Estimate Std.Err z-value P(>|z|) Std.lv Std.all
## lMS ~
## WL100 0.230 0.032 7.272 0.000 4.610 0.330
## TA100 0.337 0.071 4.770 0.000 6.762 0.213
## TH100 0.117 0.033 3.528 0.000 2.357 0.153
## LogitSurvWS ~
## Comp 2.887 0.410 7.040 0.000 0.193 0.412
## Comp ~
## Dens -0.553 0.132 -4.183 0.000 -8.246 -0.571
##
## Variances:
## Estimate Std.Err z-value P(>|z|) Std.lv Std.all
## .MSK 0.000 0.000 0.000
## .LogitSurvWS 0.183 0.012 14.950 0.000 0.183 0.830
## .Comp 0.000 0.000 0.000
## .lMS 0.002 0.000 14.950 0.000 0.707 0.707
##
## R-Square:
## Estimate
## MSK 1.000
## LogitSurvWS 0.170
## Comp 1.000
## lMS 0.293

*Code S4: Model from Fig. 5 (Code S3) with an additional variable (squared residuals=ResidualsSc2) to test for a possible non-linear relationship of mass (corrected for size) on condition. Covariance structure (indicated by operator ~~) was defined in the model because for the variable ‘residuals’ and ‘squared residuals’ we expect the error terms to be correlated.*

d1$ResidualsSc<-d1$Residuals/1000 #scaled residuals
d1$ResidualsSc2<-d1$ResidualsSc*d1$ResidualsSc #squared residuals

d1$Cho10<-d1$Cho_perVolBlood/10 #scaled cholesterol

Cmod.2 <- '

# define the composite variable
Comp<~-1*ResidualsSc+ResidualsSc2+CortStd+U_perVolBlood+Cho10+LogitH+LogitB
LogitSurvWS~Comp # regress the composite on survival
Comp~Dens # regress the density on the composite variable
# specify error variance of residuals and scaled residuals

ResidualsSc~~ResidualsSc2
'
Cmod.2.fit<-sem(Cmod.2,data=d1) # fit the model
summary(Cmod.2.fit, rsq=T, standardized=T)# get the output of the sem

## lavaan 0.6-5 ended normally after 145 iterations
##
## Estimator ML
## Optimization method NLMINB
## Number of free parameters 33
##
## Number of observations 447
##
## Model Test User Model:
##
## Test statistic 77.188
## Degrees of freedom 12
## P-value (Chi-square) 0.000
##
## Parameter Estimates:
##
## Information Expected
## Information saturated (h1) model Structured
## Standard errors Standard
##
## Composites:
## Estimate Std.Err z-value P(>|z|) Std.lv Std.all
## Comp <~
## ResidualsSc -1.000 -19.084 -0.799
## ResidualsSc2 3.903 2.147 1.818 0.069 74.489 0.197
## CortStd -0.020 0.007 -2.863 0.004 -0.383 -0.346
## U_perVolBlood -0.012 0.009 -1.375 0.169 -0.230 -0.166
## Cho10 -0.033 0.027 -1.224 0.221 -0.624 -0.166
## LogitH 0.080 0.062 1.285 0.199 1.530 0.178
## LogitB 0.030 0.022 1.347 0.178 0.569 0.152
##
## Regressions:
## Estimate Std.Err z-value P(>|z|) Std.lv Std.all
## LogitSurvWS ~
## Comp 3.587 0.505 7.100 0.000 0.188 0.401
## Comp ~
## Dens -0.368 0.104 -3.549 0.000 -7.014 -0.486
##
## Covariances:
## Estimate Std.Err z-value P(>|z|) Std.lv Std.all
## ResidualsSc ~~
## ResidualsSc2 0.000 0.000 5.693 0.000 0.000 0.280
## CortStd ~~
## U_perVolBlood 0.096 0.031 3.087 0.002 0.096 0.148
## Cho10 0.002 0.011 0.179 0.858 0.002 0.008
## LogitH 0.009 0.005 1.822 0.068 0.009 0.086
## LogitB 0.032 0.012 2.799 0.005 0.032 0.134
## Dens -0.003 0.003 -1.167 0.243 -0.003 -0.055
## U_perVolBlood ~~
## Cho10 0.013 0.009 1.407 0.159 0.013 0.067
## LogitH 0.025 0.004 5.997 0.000 0.025 0.296
## LogitB -0.019 0.009 -2.026 0.043 -0.019 -0.096
## Dens -0.007 0.002 -3.102 0.002 -0.007 -0.148
## Cho10 ~~
## LogitH 0.017 0.002 10.051 0.000 0.017 0.540
## LogitB -0.004 0.003 -1.227 0.220 -0.004 -0.058
## Dens 0.006 0.001 6.739 0.000 0.006 0.336
## LogitH ~~
## LogitB -0.000 0.001 -0.329 0.742 -0.000 -0.016
## Dens 0.002 0.000 4.598 0.000 0.002 0.223
## LogitB ~~
## Dens -0.002 0.001 -2.399 0.016 -0.002 -0.114
##
## Variances:
## Estimate Std.Err z-value P(>|z|) Std.lv Std.all
## .LogitSurvWS 0.185 0.012 14.950 0.000 0.185 0.839
## ResidualsSc 0.002 0.000 14.950 0.000 0.002 1.000
## ResidualsSc2 0.000 0.000 14.950 0.000 0.000 1.000
## .Comp 0.000 0.000 0.000
## CortStd 0.818 0.055 14.950 0.000 0.818 1.000
## U_perVolBlood 0.519 0.035 14.950 0.000 0.519 1.000
## Cho10 0.071 0.005 14.950 0.000 0.071 1.000
## LogitH 0.014 0.001 14.950 0.000 0.014 1.000
## LogitB 0.071 0.005 14.950 0.000 0.071 1.000
## Dens 0.005 0.000 14.950 0.000 0.005 1.000
##
## R-Square:
## Estimate
## LogitSurvWS 0.161
## Comp 1.000

*Code S5: R-code of the final model (Fig. 5) including latent variables (bill colour and energy reserve) and a composite variable (body condition). See Code S3 for explanation of the -1 in front of ES (composite variable). Covariance structure (indicated by operator ~~) was defined in the model because it is expected that the error term cholesterol and haematocrit are correlated. Cholesterol is determined in mmol/l blood which means that is related to the proportion of haematocrit in the blood. The R-package used for the analysis is lavaan* (Rosseel, 2012).

library(lavaan)

#scaling of the variables

f$CortStd10<-f$CortStd/10

f$nBH10<-f$nBH/10

f$MSK<-f$MS/1000

f$LS<-f$LS/100

f$CS<-f$CS/100

f$HS<-f$HS/100

f$Density<-f$Density/100

f$TA<-f$TA/100

f$TH<-f$TH/100

f$WL<-f$WL/100

f$Cho_perVolBlood<-f$Cho_perVolBlood/10

f$nHT10<-f$nHT/10

Cmod.3 <- '
BCol =~ HS *# latent variable bill colour*

ES =~ MSK+ratio *# latent variable energy store*
MSK ~ TA *# tarsus legth regressed on mass*
MSK~~0*MSK *# error variance=0*

#condition variables
lCho =~ Cho_perVolBlood *# latent variable cholesterol*
lH =~ LogitH *# latent variable haematocrit*
lB =~ LogitB *# latent variable buffy coat*
lU =~ U_perVolBlood *# latent variable uric acid*
lCort =~ CortStd10 *# latent variable corticosterone*
lB + lH + lCho + ES + lU ~ AgeN+SexN+nBH10+nHT10 *# regress confounding variables on the latent variable*
lCort + BCol ~ AgeN+SexN+nBH10 *# same here (but other set of confounding variables)*

Comp <~ -1*ES+lCort+BCol+lH+lB+lCho+lU *# composite variable*
LogitSurvWS ~ Comp *# regress composite on survival*
Comp~ Density *# regress density on composite*

#add covariance structure
lCho~~lH
lU~~lH
'
Cmod.3.fit<-sem(Cmod.3,data=f)
summary(Cmod.3.fit, standardized=T, rsq=T)

## lavaan 0.6-5 ended normally after 166 iterations
##
## Estimator ML
## Optimization method NLMINB
## Number of free parameters 47
##
## Number of observations 427
##
## Model Test User Model:
##
## Test statistic 277.638
## Degrees of freedom 52
## P-value (Chi-square) 0.000
##
## Parameter Estimates:
##
## Information Expected
## Information saturated (h1) model Structured
## Standard errors Standard
##
## Latent Variables:
## Estimate Std.Err z-value P(>|z|) Std.lv Std.all
## BCol =~
## HS 1.000 0.049 1.000
## ES =~
## MSK 1.000 0.046 0.928
## ratio 10.317 0.016 645.740 0.000 0.476 0.999
## lCho =~
## Cho_perVolBlod 1.000 0.261 1.000
## lH =~
## LogitH 1.000 0.116 1.000
## lB =~
## LogitB 1.000 0.270 1.000
## lU =~
## U_perVolBlood 1.000 0.714 1.000
## lCort =~
## CortStd10 1.000 0.100 1.000
##
## Composites:
## Estimate Std.Err z-value P(>|z|) Std.lv Std.all
## Comp <~
## ES -1.000 -0.760 -0.760
## lCort -0.229 0.071 -3.233 0.001 -0.376 -0.376
## BCol 0.121 0.135 0.901 0.368 0.097 0.097
## lH 0.050 0.069 0.719 0.472 0.095 0.095
## lB 0.013 0.024 0.544 0.586 0.059 0.059
## lCho -0.027 0.030 -0.922 0.356 -0.118 -0.118
## lU -0.013 0.010 -1.367 0.172 -0.156 -0.156
##
## Regressions:
## Estimate Std.Err z-value P(>|z|) Std.lv Std.all
## MSK ~
## TA 0.576 0.002 252.694 0.000 0.576 0.363
## lB ~
## AgeN -0.182 0.031 -5.907 0.000 -0.673 -0.276
## SexN 0.016 0.031 0.532 0.595 0.061 0.030
## nBH10 0.282 0.150 1.876 0.061 1.043 0.104
## nHT10 -0.219 0.128 -1.712 0.087 -0.811 -0.081
## lH ~
## AgeN -0.051 0.014 -3.764 0.000 -0.441 -0.181
## SexN -0.005 0.014 -0.370 0.712 -0.043 -0.021
## nBH10 -0.089 0.066 -1.343 0.179 -0.768 -0.077
## nHT10 -0.038 0.056 -0.672 0.502 -0.327 -0.033
## lCho ~
## AgeN 0.052 0.031 1.689 0.091 0.198 0.081
## SexN -0.034 0.030 -1.131 0.258 -0.132 -0.065
## nBH10 0.188 0.149 1.265 0.206 0.722 0.072
## nHT10 0.468 0.127 3.690 0.000 1.795 0.179
## ES ~
## AgeN 0.028 0.005 5.313 0.000 0.599 0.245
## SexN -0.014 0.005 -2.752 0.006 -0.310 -0.151
## nBH10 0.009 0.025 0.373 0.709 0.205 0.020
## nHT10 -0.079 0.022 -3.648 0.000 -1.708 -0.171
## lU ~
## AgeN -0.051 0.084 -0.600 0.549 -0.071 -0.029
## SexN -0.119 0.084 -1.420 0.156 -0.167 -0.082
## nBH10 -0.437 0.411 -1.063 0.288 -0.612 -0.061
## nHT10 -0.742 0.350 -2.120 0.034 -1.040 -0.104
## lCort ~
## AgeN -0.049 0.011 -4.276 0.000 -0.493 -0.202
## SexN 0.008 0.011 0.674 0.500 0.077 0.038
## nBH10 0.098 0.056 1.749 0.080 0.981 0.098
## BCol ~
## AgeN -0.026 0.005 -4.728 0.000 -0.530 -0.217
## SexN -0.025 0.005 -4.528 0.000 -0.504 -0.247
## nBH10 0.108 0.027 4.064 0.000 2.218 0.222
## LogitSurvWS ~
## Comp 3.224 0.453 7.120 0.000 0.196 0.415
## Comp ~
## Density -0.046 0.011 -4.099 0.000 -0.756 -0.526
##
## Covariances:
## Estimate Std.Err z-value P(>|z|) Std.lv Std.all
## .lCho ~~
## .lH 0.016 0.002 10.329 0.000 0.561 0.561
## .lH ~~
## .lU 0.019 0.003 5.575 0.000 0.232 0.232
##
## Variances:
## Estimate Std.Err z-value P(>|z|) Std.lv Std.all
## .MSK 0.000 0.000 0.000
## .HS 0.000 0.000 0.000
## .ratio 0.000 0.000 14.612 0.000 0.000 0.001
## .Cho_perVolBlod 0.000 0.000 0.000
## .LogitH 0.000 0.000 0.000
## .LogitB 0.000 0.000 0.000
## .U_perVolBlood 0.000 0.000 0.000
## .CortStd10 0.000 0.000 0.000
## .LogitSurvWS 0.184 0.013 14.612 0.000 0.184 0.828
## .BCol 0.002 0.000 14.612 0.000 0.893 0.893
## .ES 0.002 0.000 14.612 0.000 0.888 0.888
## .lCho 0.065 0.004 14.612 0.000 0.958 0.958
## .lH 0.013 0.001 14.866 0.000 0.961 0.961
## .lB 0.066 0.005 14.612 0.000 0.907 0.907
## .lU 0.496 0.034 14.612 0.000 0.974 0.974
## .lCort 0.009 0.001 14.612 0.000 0.943 0.943
## .Comp 0.000 0.000 0.000
##
## R-Square:
## Estimate
## MSK 1.000
## HS 1.000
## ratio 0.999
## Cho_perVolBlod 1.000
## LogitH 1.000
## LogitB 1.000
## U_perVolBlood 1.000
## CortStd10 1.000
## LogitSurvWS 0.172
## BCol 0.107
## ES 0.112
## lCho 0.042
## lH 0.039
## lB 0.093
## lU 0.026
## lCort 0.057
## Comp 1.000

fitMeasures(lvmod.5b.fit, c("chisq", "pvalue", "df","cfi", "nfi","srmr", "rmsea", "rmsea.ci.lower", "rmsea.ci.upper", "AIC", "rmsea.pvalue"))

## chisq pvalue df cfi nfi
## 277.638 0.000 52.000 0.939 0.927
## srmr rmsea rmsea.ci.lower rmsea.ci.upper aic
## 0.079 0.101 0.089 0.113 -5279.679
## rmsea.pvalue
## 0.000

*Code S6: The full model (Fig. 5) with only one size structure (tarsus length) and an added*

*direct effect of body size on survival. Body size is a latent variable in the model. For model*

*results in a figure see Fig. S13. The R-package used for the analysis is lavaan (Rosseel, 2012).*

library(lavaan)

Cmod.4 <- '
BCol =~ HS # latent variable bill colour

ES =~ MSK+ratio # latent variable energy store
MSK ~ TA # regress tarsus length on mass
MSK~~0*MSK # 0 error variance for mass

#condition variables
lCho =~ Cho_perVolBlood # latent cholesterol
lH =~ LogitH # latent haematocrit
lB =~ LogitB # latent buffy coat
lU =~ U_perVolBlood # latent uric acid
lCort =~ CortStd10 # latent corticosterone
lB + lH + lCho + ES + lU ~ AgeN+SexN+nBH10+nHT10 # regress confounding variables on latent variables
lCort + BCol ~ AgeN+SexN+nBH10 # same but with different set of variables

#composite
Comp <~ -1*ES+lCort+BCol+lH+lB+lCho+lU # composite variable
LogitSurvWS ~ Comp +TA # regress tarsus length and # # composite density on survival
Comp~ Density # regress density on composite

#add covariance structure
lCho~~lH
lU~~lH
'
Cmod.4.fit<-sem(Cmod.4,data=f)
summary(Cmod.4.fit, standardized=T, rsq=T)

## lavaan 0.6-5 ended normally after 170 iterations
##
## Estimator ML
## Optimization method NLMINB
## Number of free parameters 48
##
## Number of observations 427
##
## Model Test User Model:
##
## Test statistic 276.972
## Degrees of freedom 51
## P-value (Chi-square) 0.000
##
## Parameter Estimates:
##
## Information Expected
## Information saturated (h1) model Structured
## Standard errors Standard
##
## Latent Variables:
## Estimate Std.Err z-value P(>|z|) Std.lv Std.all
## BCol =~
## HS 1.000 0.049 1.000
## ES =~
## MSK 1.000 0.046 0.928
## ratio 10.317 0.016 645.740 0.000 0.476 0.999
## lCho =~
## Cho_perVolBlod 1.000 0.261 1.000
## lH =~
## LogitH 1.000 0.116 1.000
## lB =~
## LogitB 1.000 0.270 1.000
## lU =~
## U_perVolBlood 1.000 0.714 1.000
## lCort =~
## CortStd10 1.000 0.100 1.000
##
## Composites:
## Estimate Std.Err z-value P(>|z|) Std.lv Std.all
## Comp <~
## ES -1.000 -0.755 -0.755
## lCort -0.228 0.071 -3.188 0.001 -0.372 -0.372
## BCol 0.130 0.136 0.957 0.339 0.104 0.104
## lH 0.043 0.070 0.620 0.535 0.082 0.082
## lB 0.014 0.024 0.562 0.574 0.061 0.061
## lCho -0.027 0.030 -0.894 0.371 -0.115 -0.115
## lU -0.014 0.010 -1.429 0.153 -0.164 -0.164
##
## Regressions:
## Estimate Std.Err z-value P(>|z|) Std.lv Std.all
## MSK ~
## TA 0.576 0.002 252.687 0.000 0.576 0.363
## lB ~
## AgeN -0.182 0.031 -5.907 0.000 -0.673 -0.276
## SexN 0.016 0.031 0.532 0.595 0.061 0.030
## nBH10 0.282 0.150 1.876 0.061 1.043 0.104
## nHT10 -0.219 0.128 -1.712 0.087 -0.811 -0.081
## lH ~
## AgeN -0.051 0.014 -3.764 0.000 -0.441 -0.181
## SexN -0.005 0.014 -0.370 0.712 -0.043 -0.021
## nBH10 -0.089 0.066 -1.343 0.179 -0.768 -0.077
## nHT10 -0.038 0.056 -0.672 0.502 -0.327 -0.033
## lCho ~
## AgeN 0.052 0.031 1.689 0.091 0.198 0.081
## SexN -0.034 0.030 -1.131 0.258 -0.132 -0.065
## nBH10 0.188 0.149 1.264 0.206 0.722 0.072
## nHT10 0.468 0.127 3.690 0.000 1.795 0.179
## ES ~
## AgeN 0.028 0.005 5.313 0.000 0.599 0.245
## SexN -0.014 0.005 -2.752 0.006 -0.310 -0.151
## nBH10 0.009 0.025 0.373 0.709 0.205 0.020
## nHT10 -0.079 0.022 -3.648 0.000 -1.708 -0.171
## lU ~
## AgeN -0.051 0.084 -0.600 0.549 -0.071 -0.029
## SexN -0.119 0.084 -1.420 0.156 -0.167 -0.082
## nBH10 -0.437 0.411 -1.063 0.288 -0.612 -0.061
## nHT10 -0.742 0.350 -2.120 0.034 -1.040 -0.104
## lCort ~
## AgeN -0.049 0.011 -4.276 0.000 -0.493 -0.202
## SexN 0.008 0.011 0.674 0.500 0.077 0.038
## nBH10 0.098 0.056 1.749 0.080 0.981 0.098
## BCol ~
## AgeN -0.026 0.005 -4.728 0.000 -0.530 -0.217
## SexN -0.025 0.005 -4.528 0.000 -0.504 -0.247
## nBH10 0.108 0.027 4.064 0.000 2.218 0.222
## LogitSurvWS ~
## Comp 3.185 0.452 7.040 0.000 0.194 0.413
## TA -0.552 0.664 -0.832 0.406 -0.552 -0.037
## Comp ~
## Density -0.047 0.011 -4.095 0.000 -0.764 -0.531
##
## Covariances:
## Estimate Std.Err z-value P(>|z|) Std.lv Std.all
## .lCho ~~
## .lH 0.016 0.002 10.329 0.000 0.561 0.561
## .lH ~~
## .lU 0.019 0.003 5.575 0.000 0.232 0.232
##
## Variances:
## Estimate Std.Err z-value P(>|z|) Std.lv Std.all
## .MSK 0.000 0.000 0.000
## .HS 0.000 0.000 0.000
## .ratio 0.000 0.000 14.612 0.000 0.000 0.001
## .Cho_perVolBlod 0.000 0.000 0.000
## .LogitH 0.000 0.000 0.000
## .LogitB 0.000 0.000 0.000
## .U_perVolBlood 0.000 0.000 0.000
## .CortStd10 0.000 0.000 0.000
## .LogitSurvWS 0.183 0.013 14.612 0.000 0.183 0.828
## .BCol 0.002 0.000 14.612 0.000 0.893 0.893
## .ES 0.002 0.000 14.612 0.000 0.888 0.888
## .lCho 0.065 0.004 14.612 0.000 0.958 0.958
## .lH 0.013 0.001 14.866 0.000 0.961 0.961
## .lB 0.066 0.005 14.612 0.000 0.907 0.907
## .lU 0.496 0.034 14.612 0.000 0.974 0.974
## .lCort 0.009 0.001 14.612 0.000 0.943 0.943
## .Comp 0.000 0.000 0.000
##
## R-Square:
## Estimate
## MSK 1.000
## HS 1.000
## ratio 0.999
## Cho_perVolBlod 1.000
## LogitH 1.000
## LogitB 1.000
## U_perVolBlood 1.000
## CortStd10 1.000
## LogitSurvWS 0.172
## BCol 0.107
## ES 0.112
## lCho 0.042
## lH 0.039
## lB 0.093
## lU 0.026
## lCort 0.057
## Comp 1.000

fitMeasures(Cmod.4.fit,c("chisq", "pvalue", "df","cfi", "nfi","srmr", "rmsea", "rmsea.ci.lower","rmsea.ci.upper", "AIC", "rmsea.pvalue"))

## chisq pvalue df cfi nfi
## 276.972 0.000 51.000 0.939 0.927
## srmr rmsea rmsea.ci.lower rmsea.ci.upper aic
## 0.079 0.102 0.090 0.114 -5278.346
## rmsea.pvalue
## 0.000

*Code S7: The full model (Fig. 5) with only one size structure (tarsus length) and only the indirect effect of body size (BS) on survival. Body size is a latent variable in the model. For model results in a figure see Fig. S13. The R-package used for the analysis is lavaan (Rosseel, 2012).*

Cmod.5 <- '
BCol =~ HS

ES =~ lMS+ratio
lMS =~ MSK
BS =~ TA
lMS ~ BS
lMS~~0*lMS

#condition variables
lCho =~ Cho_perVolBlood
lH =~ LogitH
lB =~ LogitB
lU =~ U_perVolBlood
lCort =~ CortStd10
lB + lH + lCho + ES + lU ~ AgeN+SexN+nBH10+nHT10
lCort + BCol ~ AgeN+SexN+nBH10

#composite
Comp <~ -1*ES+lCort+BCol+lH+lB+lCho+lU
LogitSurvWS ~ Comp
Comp ~ Density

#add covariance structure
lCho~~lH
lU~~lH
'
Cmod.5.fit<-sem(Cmod.5,data=f)
summary(Cmod.5.fit, standardized=T, rsq=T)

## lavaan 0.6-5 ended normally after 211 iterations
##
## Estimator ML
## Optimization method NLMINB
## Number of free parameters 48
##
## Number of observations 427
##
## Model Test User Model:
##
## Test statistic 287.991
## Degrees of freedom 57
## P-value (Chi-square) 0.000
##
## Parameter Estimates:
##
## Information Expected
## Information saturated (h1) model Structured
## Standard errors Standard
##
## Latent Variables:
## Estimate Std.Err z-value P(>|z|) Std.lv Std.all
## BCol =~
## HS 1.000 0.049 1.000
## ES =~
## lMS 1.000 0.931 0.931
## ratio 10.317 0.016 645.785 0.000 0.476 0.999
## lMS =~
## MSK 1.000 0.050 1.000
## BS =~
## TA 1.000 0.031 1.000
## lCho =~
## Cho_perVolBlod 1.000 0.261 1.000
## lH =~
## LogitH 1.000 0.116 1.000
## lB =~
## LogitB 1.000 0.270 1.000
## lU =~
## U_perVolBlood 1.000 0.714 1.000
## lCort =~
## CortStd10 1.000 0.100 1.000
##
## Composites:
## Estimate Std.Err z-value P(>|z|) Std.lv Std.all
## Comp <~
## ES -1.000 -0.760 -0.760
## lCort -0.229 0.071 -3.233 0.001 -0.376 -0.376
## BCol 0.121 0.135 0.901 0.368 0.097 0.097
## lH 0.050 0.069 0.719 0.472 0.095 0.095
## lB 0.013 0.024 0.544 0.586 0.059 0.059
## lCho -0.027 0.030 -0.922 0.356 -0.118 -0.118
## lU -0.013 0.010 -1.367 0.172 -0.156 -0.156
##
## Regressions:
## Estimate Std.Err z-value P(>|z|) Std.lv Std.all
## lMS ~
## BS 0.576 0.002 252.715 0.000 0.364 0.364
## lB ~
## AgeN -0.182 0.031 -5.907 0.000 -0.673 -0.276
## SexN 0.016 0.031 0.532 0.595 0.061 0.030
## nBH10 0.282 0.150 1.876 0.061 1.043 0.104
## nHT10 -0.219 0.128 -1.712 0.087 -0.811 -0.081
## lH ~
## AgeN -0.051 0.014 -3.764 0.000 -0.441 -0.181
## SexN -0.005 0.014 -0.370 0.712 -0.043 -0.021
## nBH10 -0.089 0.066 -1.343 0.179 -0.768 -0.077
## nHT10 -0.038 0.056 -0.672 0.502 -0.327 -0.033
## lCho ~
## AgeN 0.052 0.031 1.689 0.091 0.198 0.081
## SexN -0.034 0.030 -1.131 0.258 -0.132 -0.065
## nBH10 0.188 0.149 1.265 0.206 0.722 0.072
## nHT10 0.468 0.127 3.690 0.000 1.795 0.179
## ES ~
## AgeN 0.028 0.005 5.313 0.000 0.599 0.245
## SexN -0.014 0.005 -2.752 0.006 -0.310 -0.151
## nBH10 0.009 0.025 0.373 0.709 0.205 0.020
## nHT10 -0.079 0.022 -3.648 0.000 -1.708 -0.171
## lU ~
## AgeN -0.051 0.084 -0.600 0.549 -0.071 -0.029
## SexN -0.119 0.084 -1.420 0.156 -0.167 -0.082
## nBH10 -0.437 0.411 -1.063 0.288 -0.612 -0.061
## nHT10 -0.742 0.350 -2.120 0.034 -1.040 -0.104
## lCort ~
## AgeN -0.049 0.011 -4.276 0.000 -0.493 -0.202
## SexN 0.008 0.011 0.674 0.500 0.077 0.038
## nBH10 0.098 0.056 1.749 0.080 0.981 0.098
## BCol ~
## AgeN -0.026 0.005 -4.728 0.000 -0.530 -0.217
## SexN -0.025 0.005 -4.528 0.000 -0.504 -0.247
## nBH10 0.108 0.027 4.064 0.000 2.218 0.222
## LogitSurvWS ~
## Comp 3.224 0.453 7.120 0.000 0.196 0.415
## Comp ~
## Density -0.046 0.011 -4.099 0.000 -0.756 -0.526
##
## Covariances:
## Estimate Std.Err z-value P(>|z|) Std.lv Std.all
## .lCho ~~
## .lH 0.016 0.002 10.329 0.000 0.561 0.561
## .lH ~~
## .lU 0.019 0.003 5.575 0.000 0.232 0.232
##
## Variances:
## Estimate Std.Err z-value P(>|z|) Std.lv Std.all
## .lMS 0.000 0.000 0.000
## .HS 0.000 0.000 0.000
## .ratio 0.000 0.000 14.612 0.000 0.000 0.001
## .MSK 0.000 0.000 0.000
## .TA 0.000 0.000 0.000
## .Cho_perVolBlod 0.000 0.000 0.000
## .LogitH 0.000 0.000 0.000
## .LogitB 0.000 0.000 0.000
## .U_perVolBlood 0.000 0.000 0.000
## .CortStd10 0.000 0.000 0.000
## .LogitSurvWS 0.184 0.013 14.612 0.000 0.184 0.828
## .BCol 0.002 0.000 14.612 0.000 0.893 0.893
## .ES 0.002 0.000 14.612 0.000 0.888 0.888
## BS 0.001 0.000 14.612 0.000 1.000 1.000
## .lCho 0.065 0.004 14.612 0.000 0.958 0.958
## .lH 0.013 0.001 14.866 0.000 0.961 0.961
## .lB 0.066 0.005 14.612 0.000 0.907 0.907
## .lU 0.496 0.034 14.612 0.000 0.974 0.974
## .lCort 0.009 0.001 14.612 0.000 0.943 0.943
## .Comp 0.000 0.000 0.000
##
## R-Square:
## Estimate
## lMS 1.000
## HS 1.000
## ratio 0.999
## MSK 1.000
## TA 1.000
## Cho_perVolBlod 1.000
## LogitH 1.000
## LogitB 1.000
## U_perVolBlood 1.000
## CortStd10 1.000
## LogitSurvWS 0.172
## BCol 0.107
## ES 0.112
## lCho 0.042
## lH 0.039
## lB 0.093
## lU 0.026
## lCort 0.057
## Comp 1.000

fitMeasures(Cmod.5.fit,c("chisq", "pvalue", "df","cfi", "nfi","srmr", "rmsea", "rmsea.ci.lower","rmsea.ci.upper", "AIC", "rmsea.pvalue"))

## chisq pvalue df cfi nfi
## 287.991 0.000 57.000 0.938 0.925
## srmr rmsea rmsea.ci.lower rmsea.ci.upper aic
## 0.080 0.097 0.086 0.109 -7024.833
##rmsea.pvalue
## 0.000

*Code S8: R-code and output for the SEM model used to compare the model performance for the SEM and conventional approach.*

library(lavaan)
lvmod.6 <- '
#Bill colour
BCol =~ HS

#Energy store
ES =~ lMS+ratio
lMS =~ MSK
lMS ~ TA + TH +WL
lMS~~0*lMS

#condition variables
lCho =~ Cho_perVolBlood
lH =~ LogitH
lB =~ LogitB
lU =~ U_perVolBlood
lCort =~ CortStd10
lB + lH + lCho + ES + lU ~ AgeN+SexN+nBH10+nHT10
lCort + BCol ~ AgeN+SexN+nBH10

#composite
Comp <~ -1*ES+lCort+BCol+lH+lB+lCho+lU
LogitSurvWS ~ Comp

#add covariance structure
lCho~~lH
lU~~lH
'
lvmod.6.fit<-sem(lvmod.6,data=f)
summary(lvmod.6.fit, rsq=T, standardized=T) #get model output

## lavaan 0.6-5 ended normally after 188 iterations
##
## Estimator ML
## Optimization method NLMINB
## Number of free parameters 48
##
## Number of observations 427
##
## Model Test User Model:
##
## Test statistic 246.143
## Degrees of freedom 60
## P-value (Chi-square) 0.000
##
## Parameter Estimates:
##
## Information Expected
## Information saturated (h1) model Structured
## Standard errors Standard
##
## Latent Variables:
## Estimate Std.Err z-value P(>|z|) Std.lv Std.all
## BCol =~
## HS 1.000 0.049 1.000
## ES =~
## lMS 1.000 0.928 0.928
## ratio 10.313 0.016 637.546 0.000 0.476 0.999
## lMS =~
## MSK 1.000 0.050 1.000
## lCho =~
## Cho_perVolBlod 1.000 0.261 1.000
## lH =~
## LogitH 1.000 0.116 1.000
## lB =~
## LogitB 1.000 0.270 1.000
## lU =~
## U_perVolBlood 1.000 0.714 1.000
## lCort =~
## CortStd10 1.000 0.100 1.000
##
## Composites:
## Estimate Std.Err z-value P(>|z|) Std.lv Std.all
## Comp <~
## ES -1.000 -0.873 -0.873
## lCort -0.219 0.066 -3.299 0.001 -0.413 -0.413
## BCol 0.171 0.128 1.327 0.184 0.157 0.157
## lH 0.012 0.065 0.187 0.852 0.027 0.027
## lB 0.024 0.023 1.028 0.304 0.121 0.121
## lCho -0.053 0.029 -1.842 0.065 -0.261 -0.261
## lU -0.002 0.009 -0.274 0.784 -0.033 -0.033
##
## Regressions:
## Estimate Std.Err z-value P(>|z|) Std.lv Std.all
## lMS ~
## TA 0.577 0.003 225.299 0.000 11.610 0.363
## TH -0.000 0.001 -0.333 0.739 -0.008 -0.001
## WL -0.001 0.001 -0.492 0.623 -0.011 -0.001
## lB ~
## AgeN -0.182 0.031 -5.907 0.000 -0.673 -0.276
## SexN 0.016 0.031 0.532 0.595 0.061 0.030
## nBH10 0.282 0.150 1.876 0.061 1.043 0.104
## nHT10 -0.219 0.128 -1.712 0.087 -0.811 -0.081
## lH ~
## AgeN -0.051 0.014 -3.764 0.000 -0.441 -0.181
## SexN -0.005 0.014 -0.370 0.712 -0.043 -0.021
## nBH10 -0.089 0.066 -1.343 0.179 -0.768 -0.077
## nHT10 -0.038 0.056 -0.672 0.502 -0.327 -0.033
## lCho ~
## AgeN 0.052 0.031 1.689 0.091 0.198 0.081
## SexN -0.034 0.030 -1.131 0.258 -0.132 -0.065
## nBH10 0.188 0.149 1.265 0.206 0.722 0.072
## nHT10 0.468 0.127 3.690 0.000 1.795 0.179
## ES ~
## AgeN 0.028 0.005 5.320 0.000 0.600 0.246
## SexN -0.014 0.005 -2.761 0.006 -0.311 -0.152
## nBH10 0.009 0.025 0.373 0.709 0.205 0.020
## nHT10 -0.079 0.022 -3.647 0.000 -1.708 -0.171
## lU ~
## AgeN -0.051 0.084 -0.600 0.549 -0.071 -0.029
## SexN -0.119 0.084 -1.420 0.156 -0.167 -0.082
## nBH10 -0.437 0.411 -1.063 0.288 -0.612 -0.061
## nHT10 -0.742 0.350 -2.120 0.034 -1.040 -0.104
## lCort ~
## AgeN -0.049 0.011 -4.276 0.000 -0.493 -0.202
## SexN 0.008 0.011 0.674 0.500 0.077 0.038
## nBH10 0.098 0.056 1.749 0.080 0.981 0.098
## BCol ~
## AgeN -0.026 0.005 -4.728 0.000 -0.530 -0.217
## SexN -0.025 0.005 -4.528 0.000 -0.504 -0.247
## nBH10 0.108 0.027 4.064 0.000 2.218 0.222
## LogitSurvWS ~
## Comp 3.489 0.463 7.536 0.000 0.184 0.388
##
## Covariances:
## Estimate Std.Err z-value P(>|z|) Std.lv Std.all
## .lCho ~~
## .lH 0.016 0.002 10.329 0.000 0.561 0.561
## .lH ~~
## .lU 0.019 0.003 5.575 0.000 0.232 0.232
##
## Variances:
## Estimate Std.Err z-value P(>|z|) Std.lv Std.all
## .lMS 0.000 0.000 0.000
## .HS 0.000 0.000 0.000
## .ratio 0.000 0.000 14.612 0.000 0.000 0.001
## .MSK 0.000 0.000 0.000
## .Cho_perVolBlod 0.000 0.000 0.000
## .LogitH 0.000 0.000 0.000
## .LogitB 0.000 0.000 0.000
## .U_perVolBlood 0.000 0.000 0.000
## .CortStd10 0.000 0.000 0.000
## .LogitSurvWS 0.192 0.013 14.612 0.000 0.192 0.850
## .BCol 0.002 0.000 14.612 0.000 0.893 0.893
## .ES 0.002 0.000 14.612 0.000 0.887 0.887
## .lCho 0.065 0.004 14.612 0.000 0.958 0.958
## .lH 0.013 0.001 14.866 0.000 0.961 0.961
## .lB 0.066 0.005 14.612 0.000 0.907 0.907
## .lU 0.496 0.034 14.612 0.000 0.974 0.974
## .lCort 0.009 0.001 14.612 0.000 0.943 0.943
## Comp 0.000 0.000 0.000
##
## R-Square:
## Estimate
## lMS 1.000
## HS 1.000
## ratio 0.999
## MSK 1.000
## Cho_perVolBlod 1.000
## LogitH 1.000
## LogitB 1.000
## U_perVolBlood 1.000
## CortStd10 1.000
## LogitSurvWS 0.150
## BCol 0.107
## ES 0.113
## lCho 0.042
## lH 0.039
## lB 0.093
## lU 0.026
## lCort 0.057

fitMeasures(lvmod.6.fit, c("chisq", "pvalue", "df","cfi", "srmr", "rmsea", "rmsea.ci.lower", "rmsea.ci.upper")) #get model fit indices

## chisq pvalue df cfi srmr
## 246.143 0.000 60.000 0.949 0.066
## rmsea rmsea.ci.lower rmsea.ci.upper
## 0.085 0.074 0.096

*Code S9: R-code and output of the cross validation for the SEM approach (Fig. 7a) and the conventional (regression & PCA) approach (Fig. 7b) conducted with the R-package caret* (Kuhn et al., 2018)*. method=repeatedcv means repeated cross validation, using 10 non-overlapping folds (groups; number=10). For each fold/group the dataset is split in training (p=0.8) and test dataset (being 80% training data and 20% test data). This process is repeated 3 times, meaning that 30 different sets/fits are used to estimate the performance.*

#SEM approach (Fig. 6a)

*library(caret)*
set.seed(121)
train.control <- trainControl(method = "repeatedcv",
 number = 10, repeats = 3, p=.8)
SEMmodel <- train(LogitSurvWS~Comp, data = d, method = "lm",
 trControl = train.control)
print(SEMmodel)

## Linear Regression
##
## 427 samples
## 1 predictor
##
## No pre-processing
## Resampling: Cross-Validated (10 fold, repeated 3 times)
## Summary of sample sizes: 384, 384, 385, 384, 384, 384, ...
## Resampling results:
##
## RMSE Rsquared MAE
## 0.4383024 0.1769444 0.3340294
##
## Tuning parameter 'intercept' was held constant at a value of TRUE

#Conventional approach (PCA&MR; Fig. 6b)
set.seed(125)
train.control <- trainControl(method = "repeatedcv",
 number = 10, repeats = 3, p=.8)
PCAmodel <- train(LogitSurvWS~predict, data = d, method = "lm",
 trControl = train.control) #“predict” is the predicted # value of survival from the MR&PCA approach (Fig. 6b)
print(PCAmodel)

## Linear Regression
##
## 427 samples
## 1 predictor
##
## No pre-processing
## Resampling: Cross-Validated (10 fold, repeated 3 times)
## Summary of sample sizes: 383, 385, 384, 383, 385, 384, ...
## Resampling results:
##
## RMSE Rsquared MAE
## 0.4420557 0.1625398 0.3323978
##
## Tuning parameter 'intercept' was held constant at a value of TRUE

## RMSE of this (conventional approach) is higher than in the SEM and R2 is *##* lower, indicating lower model performance. Also the MAE, the mean *#*# absolute error is lower in SEM, indicating better performance. MAE ## expresses similar to the RMSE the average model prediction error. *##* However, since in RMSE the errors are squared before they are averaged, ## the RMSE gives a relatively high weight to large errors.

*Code S10: Example r-code to account for a measurement error (in a regression analysis; mass as response variable and three structural size measures as independent variables) by using latent variables in SEM. We assume a measurement repeatability (intra-class correlation coefficient) of 0.3 of the structural size measures TA, TH and WL meaning that the measurement error is 0.7. We add three latent variables lTA, lTH, lWL to the model. Instead of regressing the structural size measures directly on mass, we regress the latent variables of the structural size measures on mass (lTA, lWL, lTH). See Fig. S25 for a schematic illustration of the model. By adding the latent variables to the model, we can distinguish between the error variance due to unexplained variation and due to measurement error. In the output of this model, we find the unexplained variation for each structural size variable (without any influence of measurement error). The R-package used for the analysis is lavaan* (Rosseel, 2012). *See chapter 4 in* Grace (2006*) for more information about accounting for measurement errors in SEM.*

library(lavaan)

lvmod.3 <-'
lTA=~TA # define the latent variable lTA
lWL=~WL # define the latent variable lWL
lTH=~TH # define the latent variable lTH
TA~~0.7*TA # add error variance on TA
TH~~0.7*TH # add error variance on TH
WL~~0.7*WL # add error variance on WL
MS~lTA+lTH+lWL # regress the three latent variables on mass
'
lvmod.3.fit<-sem(lvmod.3,data=M2) #fit the model; for reproducing the code # see the Rmark down file (Analysis.Rmd) with the data file (data.csv)
summary(lvmod.3.fit, rsq=T, standardized=T) #get model output

## lavaan 0.6-5 ended normally after 62 iterations
##
## Estimator ML
## Optimization method NLMINB
## Number of free parameters 10
##
## Used Total
## Number of observations 1049 1061
##
## Model Test User Model:
##
## Test statistic 0.000
## Degrees of freedom 0
##
## Parameter Estimates:
##
## Information Expected
## Information saturated (h1) model Structured
## Standard errors Standard
##
## Latent Variables:
## Estimate Std.Err z-value P(>|z|) Std.lv Std.all
## lTA =~
## TA 1.000 2.924 0.961
## lWL =~
## WL 1.000 7.035 0.993
## lTH =~
## TH 1.000 6.402 0.992
##
## Regressions:
## Estimate Std.Err z-value P(>|z|) Std.lv Std.all
## MS ~
## lTA 4.391 0.559 7.852 0.000 12.839 0.248
## lTH 1.349 0.245 5.514 0.000 8.636 0.167
## lWL 1.841 0.217 8.470 0.000 12.949 0.250
## gives the path strength (impact of TA, WL and TH) on mass (MS) ## accounting for imperfect measurements of TH, TA and WL, thus being lower *##* than in previous SEMs (e.g. Code S3).
## Covariances:
## Estimate Std.Err z-value P(>|z|) Std.lv Std.all
## lTA ~~
## lWL 7.027 0.700 10.042 0.000 0.342 0.342
## lTH 7.293 0.647 11.278 0.000 0.390 0.390
## lWL ~~
## lTH 14.386 1.481 9.717 0.000 0.319 0.319
##
## Variances:
## Estimate Std.Err z-value P(>|z|) Std.lv Std.all
## .TA 0.700 0.700 0.076
## .TH 0.700 0.700 0.017
## .WL 0.700 0.700 0.014
## .MS 1999.265 88.045 22.707 0.000 1999.265 0.747
## lTA 8.548 0.404 21.168 0.000 1.000 1.000
## lWL 49.497 2.192 22.583 0.000 1.000 1.000
## lTH 40.982 1.820 22.517 0.000 1.000 1.000
##
## R-Square:
## Estimate
## TA 0.924
## TH 0.983
## WL 0.986
## MS 0.253

## R2 gives unexplained variation without the impact of measurement error.

*Code S11: Example r-code for a model with binomial response variable. In this example we defined a bird that was seen in the subsequent year (after catching) as 1 and otherwise as 0. We show here an example of a simple composite model (similar to Fig. 5) without any confounding variables. The R-package used for the analysis is lavaan (Rosseel, 2012).*

library(lavaan)

d$Seen1<-ordered(d$Seen) #define the numeric binomial response variable "Seen" (1/0) to be ordered

Cmod.5 <- '
Comp <~ 1*MS+Cho_perVolBlood+LogitB+LogitH+U_perVolBlood+Cort

#define the composite variable
MS~TA # regress tarsus length on mass to correct for size effect on mass
Seen1~Comp # use Seen1 as binomial response variable
'

Cmod.5.fit<-sem(Cmod.5,data=d, ordered ="Seen1", estimator="WLSMV")

#estimator WLSMV needs to be used when dealing with binomial response #variables, define ordered="Seen1" to tell the model that the response #variable is binomial
summary(Cmod.5.fit, rsq=T, standardized=T) #get model output

## lavaan 0.6-5 ended normally after 115 iterations
##
## Estimator DWLS
## Optimization method NLMINB
## Number of free parameters 10
##
## Used Total
## Number of observations 110 214
##
## Model Test User Model:
## Standard Robust
## Test Statistic 4.603 4.645
## Degrees of freedom 6 6
## P-value (Chi-square) 0.596 0.590
## Scaling correction factor 1.096
## Shift parameter 0.445
## for the simple second-order correction
##
## Parameter Estimates:
##
## Information Expected
## Information saturated (h1) model Unstructured
## Standard errors Robust.sem
##
## Composites:
## Estimate Std.Err z-value P(>|z|) Std.lv Std.all
## Comp <~
## MS 1.000 0.017 0.607
## Cho_perVolBlod -5.295 8.020 -0.660 0.509 -0.092 -0.228
## LogitB 88.799 76.314 1.164 0.245 1.541 0.416
## LogitH 123.789 207.183 0.597 0.550 2.148 0.219
## U_perVolBlood 0.819 41.349 0.020 0.984 0.014 0.006
## Cort -14.552 9.206 -1.581 0.114 -0.252 -0.778
##
## Regressions:
## Estimate Std.Err z-value P(>|z|) Std.lv Std.all
## MS ~
## TA 4.639 1.075 4.316 0.000 4.639 0.407
## Seen1 ~
## Comp 0.008 0.003 2.387 0.017 0.441 0.414
##
## Intercepts:
## Estimate Std.Err z-value P(>|z|) Std.lv Std.all
## .MS 143.753 134.311 1.070 0.284 143.753 4.111
## .Seen1 0.000 0.000 0.000
## Comp 0.000 0.000 0.000
##
## Thresholds:
## Estimate Std.Err z-value P(>|z|) Std.lv Std.all
## Seen1|t1 2.651 5.553 0.477 0.633 2.651 2.489
##
## Variances:
## Estimate Std.Err z-value P(>|z|) Std.lv Std.all
## .MS 1019.968 144.232 7.072 0.000 1019.968 0.834
## .Seen1 0.940 0.940 0.829
## Comp 0.000 0.000 0.000
##
## Scales y*:
## Estimate Std.Err z-value P(>|z|) Std.lv Std.all
## Seen1 1.000 1.000 1.000
##
## R-Square:
## Estimate
## MS 0.166
## Seen1 0.171

fitmeasures(Cmod.5.fit, c("chisq", "pvalue", "df","cfi", "srmr", "rmsea", "rmsea.ci.lower", "rmsea.ci.upper")) #get model fit indices

## chisq pvalue df cfi srmr
## 4.603 0.596 6.000 1.000 0.018
## rmsea rmsea.ci.lower rmsea.ci.upper
## 0.000 0.000 0.107

# References

Allen, A. M., Ens, B. J., van de Pol, M., van der Jeugd, H., Frauendorf, M., Oosterbeek, K., & Jongejans, E. (2019). Seasonal survival and migratory connectivity of the Eurasian Oystercatcher revealed by citizen science. *Auk*, *136*(1), 1–17. https://doi.org/10.1093/auk/uky001

Alonso-Alvarez, C., Ferrer, M., & Velando, A. (2002). The plasmatic index of body condition in Yellow-legged Gulls *Larus cachinnans*: A food-controlled experiment. *Ibis*, *144*, 147–149. https://doi.org/10.1046/j.0019-1019.2001.00023.x

Bakker, W., Ens, B. J., Dokter, A., van der Kolk, H.-J., Rappoldt, K., van de Pol, M., Troost, K., van der Veer, H. W., Bijleveld, A. I., van der Meer, J., Oosterbeek, K., Jongejans, E., & Allen, A. M. (2021). Connecting foraging and roosting areas reveals how food stocks explain shorebird numbers. *Estuarine, Coastal and Shelf Science*, *259*, 107458. https://doi.org/10.1016/j.ecss.2021.107458

Bentler, P. M. (1990). Comparative fit indexes in structural models. *Psychological Bulletin*, *107*, 238-246. https://doi.org/10.1037/0033-2909.107.2.238

Birkhead, T. R., Pellatt, E. J., Matthews, I. M., Roddis, N. J., Hunter, F. M., McPhie, F., & Castillo-Juarez, H. (2006). Genic capture and the genetic basis of sexually selected traits in the zebra finch. *Evolution*, *60*(11), 2389–2398. https://doi.org/10.1111/j.0014-3820.2006.tb01873.x

Campbell, N. A., Reece, J. B., Urry, L. A., Cain, M. L., Wasserman, S. A., Minorsky, P. V., & Jackson, R. B. (2008). *Biology* (Eight). Pearson Benjamin Cummings.

Cote, J., Arnoux, E., Sorci, G., Gaillard, M., & Faivre, B. (2009). Age-dependent allocation of carotenoids to coloration versus antioxidant defences. *Journal of Experimental Biology*, *213*(2), 271–277. https://doi.org/10.1242/jeb.035188

Cramp, S., & Simmons, K. E. L. (1983). *Birds of the Western Palearctic. Waders to gulls: Vol. III*. Oxford University Press.

Durell, S. E. A. L. V. D., Goss-Custard, J. D., & Caldow, R. W. G. (1993). Sex-related differences in diet and feeding method in the oystercatcher *Haematopus ostralegus*. *Journal of Animal Ecology*, *62*, 205–215. https://doi.org/10.2307/5495

Eraud, C., Devevey, G., Gaillard, M., Prost, J., Sorci, G., & Faivre, B. (2007). Environmental stress affects the expression of a carotenoid-based sexual trait in male zebra finches. *Journal of Experimental Biology*, *210*(20), 3571–3578. https://doi.org/10.1242/jeb.005496

Grace, J. B. (2006). *Structural Equation Modeling and Natural Systems*. Cambridge University Press.

Grace, J. B., & Keeley, J. E. (2006). A structural equation model analysis of postfire plant diversity in california shrublands. *Ecological Applications*, *16*(2), 503–514. https://doi.org/10.1890/1051-0761(2006)016[0503:asemao]2.0.co;2

Griminger, P. (1986). Lipid Metabolism. In P. D. Sturkie (Ed.), *Avian Physiology*. Springer.

Hornman, M., Hustings, F., Koffijberg, K., & Klaassen, O. (2012). *Handleiding Sovon Watervogel- en Slaapplaatstellingen* (p. 28). Sovon Vogelonderzoek Nederland.

Hu, L., & Bentler, P. M. (1999). Cutoff criteria for fit indexes in covariance structure analysis: Conventional criteria versus new alternatives. *Structural Equation Modeling: A Multidisciplinary Journal*, *6*(1), 1–55. https://doi.org/10.1080/10705519909540118

Jak, S., & Jorgensen, T. D. (2017). Relating measurement invariance, cross-level invariance, and multilevel reliability. *Front Psychol*, *8*, 1640. https://doi.org/10.3389/fpsyg.2017.01640

Jimeno, B., Briga, M., Verhulst, S., & Hau, M. (2017). Effects of developmental conditions on glucocorticoid concentrations in adulthood depend on sex and foraging conditions. *Hormones and Behavior*, *93*, 175–183. https://doi.org/doi.org/10.1016/j.yhbeh.2017.05.020

Jimeno, B., Hau, M., & Verhulst, S. (2017). Strong association between corticosterone levels and temperature-dependent metabolic rate in individual zebra finches. *The Journal of Experimental Biology*, *220*(23), 4426–4431. https://doi.org/10.1242/jeb.166124

Jimeno, B., Hau, M., & Verhulst, S. (2018). Corticosterone levels reflect variation in metabolic rate, independent of ‘stress.’ *Scientific Reports*, *8*(1). https://doi.org/10.1038/s41598-018-31258-z

Kassambara, A., & Mundt, F. (2020). *Factoextra: Extract and visualize the results of multivariate data analyses. R package version 1.0.7*.

Kline, R. B. (2011). *Principles and Practice of Structural Equation Modeling* (3rd ed.). The Guilford Press.

Kuhn, M., Contributions from Wing, J., Weston, S., Williams, A., Keefer, C., Engelhardt, A., Cooper, T., Mayer, Z., Kenkel, B., R Core Team, Benesty, M., Lescarbeau, R., Ziem, A., Scrucca, L., Tang, Y., Candan, C., & Hunt, T. (2018). *caret: Classification and regression training*.

Laake, J. L. (2013). *RMark: An R interface for analysis of capture – recapture data with MARK.* AFSC Processed Report.

Le, S., Josse, J., & Husson, F. (2008). FactoMineR: An R Package for Multivariate Analysis. *Journal of Statistical Software*, *25*(1), 1–18. https://doi.org/10.18637/jss.v025.i01

Lefcheck, J. S. (2019). *Structural Equation Modeling in R for Ecology and Evolution*.

Marra, P. P., & Holberton, R. L. (1998). Corticosterone levels as indicators of habitat quality: Effects of habitat segregation in a migratory bird during the non-breeding season. *Oecologia*, *116*, 284–292.

Milenkaya, O., Catlin, D. H., Legge, S., & Walters, J. R. (2015). Body condition indices predict reproductive success but not survival in a sedentary, tropical bird. *PLOS ONE*, *10*(8), e0136582. https://doi.org/10.1371/journal.pone.0136582

Nienhuis, J. (2015). *Pixel Grabber, version 1.4.*

Norte, A. C., Ramos, J. A., Sousa, J. P., & Sheldon, B. C. (2009). Variation of adult Great Tit *Parus major* body condition and blood parameters in relation to sex, age, year and season. *Journal of Ornithology*, *150*(3), 651. https://doi.org/10.1007/s10336-009-0387-1

Perez-Rodriguez, L. (2009). Carotenoids in evolutionary ecology: Re-evaluating the antioxidant role. *Bioessays*, *31*(10), 1116–1126. https://doi.org/10.1002/bies.200900070

Quesada, J., & Senar, J. C. (2006). Comparing plumage colour measurements obtained directly from live birds and from collected feathers: The case of the great tit *Parus major*. *Journal of Avian Biology*, *37*, 609–616. https://doi.org/10.1111/j.0908-8857.2006.03636.x

R Core Team. (2019). *R: A language and environment for statistical computing. R Foundation for Statistical Computing, Vienna, Austria.*

Rosseel, Y. (2012). lavaan: An R Package for Structural Equation Modeling. *Journal of Statistical Software*, *48*(2), 1–36. https://doi.org/10.18637/jss.v048.i02

Shipley, B. (2016). *Cause and Correlation in Biology—A User’s Guide to Path Analysis, Structural Equations and Causal Inference with R* (2nd ed.). Cambridge University Press.

Simons, M. J. P., Briga, M., Koetsier, E., Folkertsma, R., Wubs, M. D., Dijkstra, C., & Verhulst, S. (2012). Bill redness is positively associated with reproduction and survival in male and female zebra finches. *PLoS ONE*, *7*(7), e40721. https://doi.org/10.1371/journal.pone.0040721

Simons, M. J. P., Cohen, A. A., & Verhulst, S. (2012). What does carotenoid-dependent coloration tell? Plasma carotenoid level signals immunocompetence and oxidative stress state in birds-A meta-analysis. *PLoS ONE*, *7*(8), e43088. https://doi.org/10.1371/journal.pone.0043088

Steiger, J. H. (2007). Understanding the limitations of global fit assessment in structural equation modeling. *Personality and Individual Differences*, *42*(5), 893–898.

Stevens, M., Partridge, J., Párraga, C. A., Troscianko, T. S., & Innes, C. (2007). Using digital photography to study animal coloration. *Biological Journal of the Linnean Society*, *90*, 211–237.

Tsahar, E., Arad, Z., Izhaki, I., & Guglielmo, C. G. (2006). The relationship between uric acid and its oxidative product allantoin: A potential indicator for the evaluation of oxidative stress in birds. *Journal of Comparative Physiology B*, *176*(7), 653–661. https://doi.org/10.1007/s00360-006-0088-5

Van de Pol, M., Ens, B. J., Oosterbeek, K., Brouwer, L., Verhulst, S., Tinbergen, J. M., Rutten, A. L., & De Jong, M. (2009). Oystercatchers’ bill shapes as a proxy for diet specialization: More differentiation than meets the eye. *Ardea*, *97*(3), 335–347. https://doi.org/10.5253/078.097.0309

Verhulst, S., Oosterbeek, K., Rutten, A. L., & Ens, B. J. (2004). Shellfish fishery severely reduces condition and survival of oystercatchers despite creation of large marine protected areas. *Ecology and Society*, *9*(1), 17. https://doi.org/10.5751/es-00636-090117

Villafuerte, R., & Negro, J. J. (1998). Digital imaging for colour measurement in ecological research. *Ecology Letters*, *1*, 151–154. https://doi.org/10.1046/j.1461-0248.1998.00034.x

Von Schantz, T., Bensch, S., Grahn, M., Hasselquist, D., & Wittzell, H. (1999). Good genes, oxidative stress and condition-dependent sexual signals. *Proceedings of the Royal Society B-Biological Sciences*, *266*(1414), 1–12. https://doi.org/10.1098/rspb.1999.0597

Wolak, M. E., Fairbairn, D. J., & Paulsen, Y. R. (2012). Guidelines for estimating repeatability. *Methods in Ecology and Evolution*, *3*(1), 129–137. https://doi.org/10.1111/j.2041-210X.2011.00125.x
